# SpaCEy links spatial tissue patterns to clinical outcomes using explainable graph neural networks

**DOI:** 10.1038/s41467-026-77924-z

**Published:** 2026-09-26

**Authors:** Ahmet Sureyya Rifaioglu, Egle Helene Ervin, Ahmet Sarigun, Deniz Germen, Bernd Bodenmiller, Jovan Tanevski, Julio Saez-Rodriguez

**Affiliations:** 1 Institute for Computational Biomedicine, Heidelberg University and Heidelberg University Hospital, Heidelberg, Germany; 2 Department of Quantitative Biomedicine, University of Zurich, Zurich, Switzerland; 3 Bioinformatics and Omics Data Science Platform, Max Delbruck Center for Molecular Medicine - Berlin Institute for Molecular Systems Biology, Berlin, Germany; 4 Department of Computer Engineering, Middle East Technical University, Ankara, Türkiye; 5 Translational Spatial Profiling Center, Heidelberg University Hospital, Heidelberg, Germany; 6 European Molecular Biology Laboratory, European Bioinformatics Institute (EMBL-EBI), Hinxton, UK

**Keywords:** Machine learning, Cancer microenvironment, Computational models, Computer science, Translational research

## Abstract

Tissues are complex ecosystems organised in space, and alterations in this organisation underpin multiple diseases. Spatial omics enables molecular profiling of tissue organisation, but linking these patterns to clinical outcomes remains challenging. We present SpaCEy (**Spa**tial **C**linical **E**xplainabilit**y**), an explainable graph neural network that identifies tissue patterns predictive of clinical outcomes in spatial proteomics datasets. SpaCEy models tissues as spatial graphs from molecular marker expression, without using predefined cell-type labels or anatomical regions as model inputs. Its embeddings capture intercellular relationships and molecular dependencies for predicting overall survival and disease progression. An integrated explainer identifies recurring spatial patterns and coordinated marker expression relevant to model predictions. Applied to a spatial proteomic lung cancer cohort, SpaCEy identifies spatial and protein-expression patterns associated with disease progression. Across multiple breast cancer proteomic datasets, it stratifies patients by overall survival, both across and within established clinical subtypes, and highlights protein markers underlying this stratification.

## Abstract (web summary)

SpaCEy uses explainable graph neural networks to link spatial tissue organisation with clinical outcomes, identifying spatial and molecular patterns associated with cancer progression and patient survival.

## Introduction

Cells often organize into specific arrangements in space within tissues and the relationship between the spatial organisation of tissues and function, both in health and disease, is widely acknowledged^1,2^. The amount of spatially resolved molecular data at the single-cell level has been rapidly increasing with technological improvements^3^. In particular, advancements in multiplex imaging enable precise mapping of cell populations and their diverse states within their tissue environment at the single-cell level, exploration of the organisation and interaction of cells in the tumour microenvironment, communication patterns, and novel disease mechanisms^3,4^. Spatial omics also offers new translational opportunities by revealing similarities and differences in tissue organisation and function among patients assigned to the same treatment group^1,3,5–7^. This tissue organisation frequently shows characteristic changes in spatial arrangements of cells which are shared across patients in diseases like cancer and fibrosis. Identifying these changes is critical because they are often structured as spatially important regions, which are localised structural patterns of cells and molecules within the tissue^8^. The high resolution offered by spatial omics technologies now makes it possible to identify these spatial motifs, providing an opportunity to link this spatial variation directly to clinical outcomes.

Analysing complex, high-dimensional omics data requires advanced machine learning methods capable of integrating diverse datasets, modelling relationships within and across tissues, and extracting meaningful biological insights to support the prediction of clinical outcomes. Subsequently, such models allow for studying the link between the changes in these relationships in response to perturbations, such as disease or drug treatment. Some of these methods do not incorporate spatial information for clinical outcome prediction. For example, Fast Survival SVM applies a max-margin framework for time-to-event prediction for survivability prediction^9^, Random Survival Forests use ensemble tree models to capture nonlinear relationships between omics data and survival outcomes^10^, and Gradient Boosting Survival Analysis builds an ensemble of decision-tree models in a stage-wise manner to capture nonlinear predictors of time-to-event outcomes^11^. In contrast, spatially informed approaches have been developed to model tissue organisation directly, enabling the integration of spatial coordinates to solve complex tasks such as clinical outcome prediction and characterisation of tissue microenvironments. For instance, Wu et al. developed a graph neural network (GNN) to model tumour microenvironments using spatial protein profiles from tissue specimens^12^. This method leverages multiplexed immunofluorescence imaging to capture the complex interactions within the tumour microenvironment and predict patient outcomes based on local cell type compositions. Similarly, STELLAR combines spatial and molecular information to enhance cell-type annotation and discovery in spatially resolved single-cell datasets. Using geometric deep learning, it assigns cells to known types, identifies novel cell states, and captures higher-order tissue structures^13^. SPACE-GM models tumour microenvironments as cellular graphs with the aim of predicting cancer recurrence and patient survival based on cell-type compositions of neighbourhoods^12^. A recent study by Ali et al. applied GNNs to spatial proteomics data, showing their models capture meaningful spatial features by analysing the embeddings of the samples^14^. Graph-based neural approaches have also been developed extensively for spatial transcriptomics, including methods for spatial-domain detection, representation learning, integration, and deconvolution such as SpaGCN^15^, STAGATE^16^, GraphST^17^, DeepST^18^, SEDR^19^ and SpaceFlow^20^. However, these methods have largely focused on characterising tissue organisation, while important limitations remain in connecting spatial patterns to clinical outcomes and translating model predictions into biologically interpretable tissue-level insights. Overall, spatially aware methods highlight the value of incorporating tissue architecture into single-cell analysis, enabling graph neural networks to uncover deeper insights into cellular function and tissue organisation.

Despite recent developments of spatially informed methods for analysing tissue organisation, major limitations remain in translating predictive power into biological or clinical insight. One of the key limitations, when applied to spatial tissue graphs, existing methods often rely on predefined cell-type labels or local clustering, which prevents the discovery of emergent organisational patterns directly from continuous molecular measurements. At the same time, the aforementioned machine learning models, despite their strong predictive performance, fall short in identifying contiguous, spatially coherent regions of importance and in effectively incorporating spatial information into their modelling frameworks^21^. They typically limit interpretability to analyses of model-derived latent representations or cell-level importance scores, thereby limiting their ability to uncover spatial motifs that are critical to clinical outcomes. Consequently, current methods lack the capacity to simultaneously revealing the spatially important regions and providing a concise set of molecular markers that collectively drive the observed clinical outcomes. Addressing these limitations is essential for improving confidence in model decisions, reliability, and the potential of obtaining novel biological insights^22^, and to ultimately deploy machine learning models in clinical practice.

We present SpaCEy, an explainable method for discovering spatially important regions on the tissues associated with clinical outcomes using GNNs. Unlike approaches that require predefined cell-type labels or clustering as model inputs, SpaCEy identifies shared contiguous spatial patterns directly from measured cell-specific marker abundances. By representing each tissue sample as a spatial graph and learning predictive embeddings, SpaCEy bridges the gap between tissue architecture and clinical stratification. To overcome limitations of current approaches, which often produce predictive embeddings without revealing the structural or molecular features driving their predictions, SpaCEy integrates an explainer model that highlights connected sets of spatially adjacent nodes and edges responsible for its outputs by identifying compact subgraphs^23^. This enables interpretable insights into tissue organisation and molecular determinants of disease. Furthermore, SpaCEy identifies a concise set of marker proteins that are most influential for predicting the corresponding clinical outcomes, providing molecular-level interpretability alongside spatial insights. This workflow is annotation-agnostic during model input, training, prediction, and explanation generation. Annotations are only used to assist post-hoc analysis to contextualise the interpretation to particular groups of samples of interest. By capturing emergent patterns across patients, SpaCEy links complex spatial structures to clinical outcomes. By offering a scalable, explainable and data-driven framework for analysing tissue organisation, SpaCEy paves the way for insights into the spatial determinants of disease and their impact on patient outcomes.

## Results

### Model Architecture and Analysis Pipeline

**Fig. 1:**
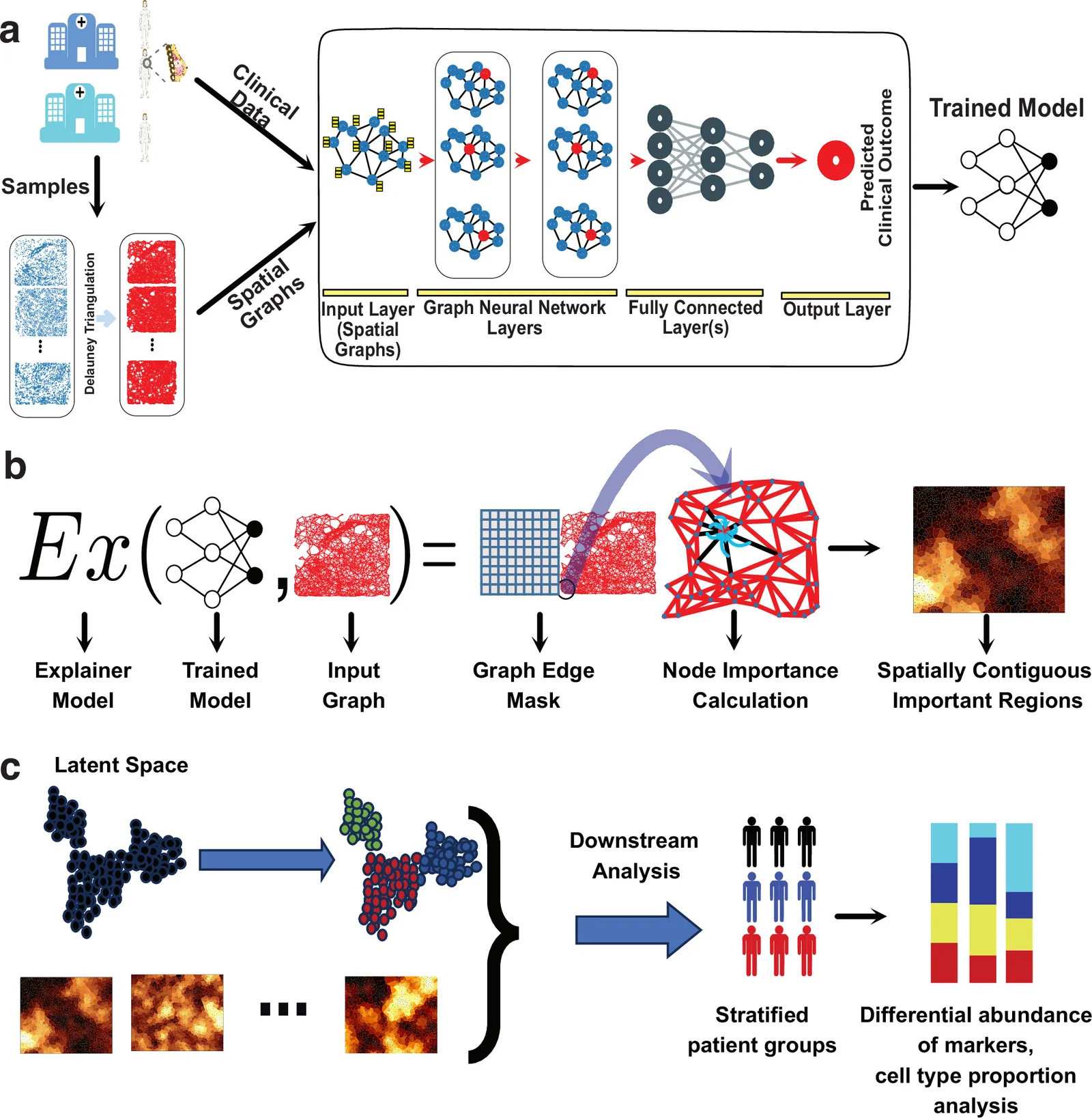
SpaCEy model architecture and analysis workflow. **a** Samples from multiple cohorts were collected as the source of high-dimensional spatial data for this study. These samples were converted into spatial graphs using the Delaunay triangulation method, and a graph neural network (GNN) was trained on these graphs, integrating spatial information and clinical data to predict relevant clinical outcomes. **b** The trained GNN model is then analysed using the explainer model, which outputs learned edge masks. Node importance values are computed by aggregating edge-mask values within the k-hop neighbourhood of each node, enabling the identification of compact connected subgraphs that define important spatial regions in the tissue. **c** Latent representations of samples and identified important regions were then used for visualisation, differential analysis, and biological interpretation based on node importance values, ultimately enabling patient stratification and downstream analysis. The female icon in panel **a** was obtained from Servier Medical Art (https://smart.servier.com/) and is licensed under CC BY 4.0. The breast icon in panel **a** was obtained from Bioicons (https://bioicons.com/?query=breast) and is licensed under CC BY 3.0 Unported. Source data are provided as a Source Data file.

SpaCEy is an explainable method that detects spatial patterns in tissue samples which are linked to clinical outcomes. The model input consists of protein-abundance measurements at single-cell level represented as spatial graphs; clinical outcomes are used as supervised labels, while clinical annotations are only retained for descriptive and post hoc interpretation (Methods, Data preprocessing and Training settings) (Fig. 1a). Cell-type and compartment annotations are not used as SpaCEy node features, but are retained as metadata for descriptive and post hoc biological interpretation. The spatially resolved samples are transformed into a spatial graph by Delaunay triangulation method^24^. This approach ensures that each cell is connected to its spatially nearest neighbours, preserving the tissue architecture in the graph structure. In these spatial graphs, each node represents a cell, and the features of each node represent the abundance of each measured protein marker in the corresponding spatial location. A GNN model is then trained on this spatial graph representation, leveraging the connectivity and spatial relationships between cells to learn meaningful latent representations of the input samples (Methods, Model architecture) (Fig. 1a). To interpret the learned model, an explainer model generates feature and edge masks that highlight the most relevant components contributing to the GNN’s predictions for the clinical outcome of interest (Methods, Explainer model) (Fig. 1b)^23^. The input to the explainer model consists of the trained model and constructed spatial graph, and its output is the learned edge masks (i.e., edge importance values) for the input graph. A key novelty of SpaCEy lies in its ability to generate interpretable spatially contiguous regions (i.e., connected subgraphs) that uncover spatially localised molecular drivers of clinical outcomes. Node importance values are computed by aggregating edge-mask values from k-hop neighbours of each node, enabling the identification of key spatially contiguous, compact subgraphs that mark important regions in the tissue (Methods, Node importance and important regions). Here, node and edge importance scores produced by the explainer quantify unsigned relevance magnitudes. Thus, they identify the graph components that are most influential for the model prediction, but they do not by themselves encode the direction of effect, i.e., whether a given node, edge, or spatial motif shifts the prediction toward or away from a clinical outcome of interest. Using these importance values together with the latent representations from the trained model, we perform unsupervised analysis of the latent space representations of samples, and perform differential analysis to extract biologically relevant insights (Fig. 1c). Based on the identified molecular and spatial features, SpaCEy provides a data-driven framework for understanding the heterogeneity of structure and function in the tumour microenvironment and enables an improved patient stratification (Fig. 1c).

### SpaCEy identifies spatially organised protein and cellular patterns predictive of tumour progression

**Fig. 2:**
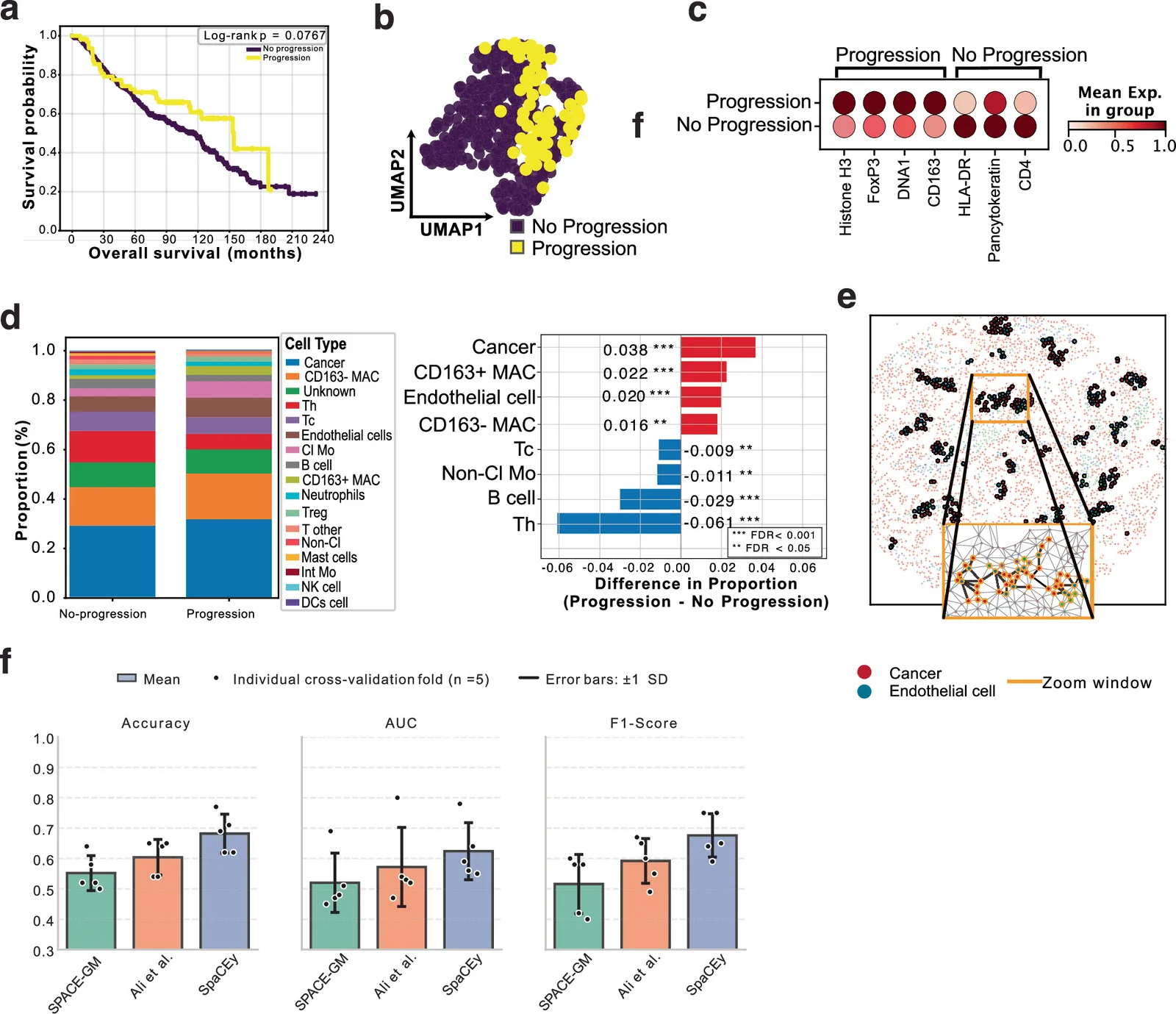
Progression-associated spatial and cellular patterns in lung adenocarcinoma. **a** Kaplan--Meier estimates of overall survival for patients stratified by progression status, with censoring marks shown on each curve. Survival distributions were compared using a non-directional omnibus log-rank test. No adjustment for multiple comparisons was applied because a single global comparison was performed. **b** A two-dimensional UMAP projection of sample-level embeddings generated from spatial proteomic features, showing the organisation of samples in a low-dimensional manifold based on the clinical outcome of patients **c**. Differential abundance analysis identifying distinct protein markers between progressor and non-progressor patients. **d** Cell type proportions of important nodes in the LUAD dataset (top panel) and significant proportional differences between progression and no-progression groups (bottom panel). Positive values indicate enrichment in the progression group, while negative values indicate enrichment in the no-progression group. **e** Representative progressor LUAD sample showing important cancer and endothelial cell nodes. The zoomed region highlights direct edges in the input spatial graph between important cancer and endothelial cells **f**. Comparison of SpaCEy with the state-of-the-art methods. Individual points represent results from each cross-validation fold (n = 5 folds per method). Bars represent the arithmetic mean. Error bars are centred on the arithmetic mean and extend  ± 1 sample standard deviation across folds. The same fixed, progression-stratified folds were used for all methods. Source data are provided as a Source Data file.

We first applied SpaCEy to a recently published lung adenocarcinoma (LUAD) imaging mass cytometry data by Sorin et al.^25^ comprising samples from 416 patients. In the LUAD cohort, we modelled progression as a supervised binary classification task using the progression annotations provided with the dataset. Summary statistics and cohort characteristics of LUAD dataset is provided in Supplementary Table 1. Fig. 2a shows Kaplan–Meier estimates of overall survival stratified by progression status, with censoring marks indicated on each curve, revealing no significant difference in overall survival between the two progression groups (log-rank *p* = 0.0767). To investigate the relationship between the clinical outcomes and the latent representations generated by our model, we visualised the embeddings across all nodes for each sample in UMAP space (Fig. 2b), with each point representing a sample and coloured by progression status. Together, these visualisations demonstrate that the learned embedding is associated with clinical progression.

Analysis of marker abundance in the important nodes revealed distinct immune microenvironments and tumour-associated profiles between patients with and without progression Fig. 2c. The T-cell labels follow the source annotations from Sorin et al.^25^: Th cells refers to CD4-enriched helper T cells, Tc refers to CD8a-enriched cytotoxic T cells, Treg refers to FOXP3-enriched regulatory T cells, and T other denotes T cells not assigned to these specific subsets. Here, important nodes refer to a group of cells selected by the explanation module as part of model-informative spatial regions. In contrast, non-progressing tumours exhibited areas with elevated numbers of Th cells, Tc cells, and B cells. Specifically, FOXP3, CD163, Histone H3, and DNA1 were enriched in the important nodes of progression group. FOXP3 is a canonical regulatory T-cell marker, and CD163^+^ macrophages form part of an immunosuppressive axis previously associated with aggressive LUAD architecture and poor outcomes^25^. In contrast, non-progressing patients exhibited areas with higher expression of HLA-DR and CD4, and a slight upregulation of epithelial cancer cell marker pan-cytokeratin. HLA-DR marks primarily antigen-presenting cells that are part of an active anti-tumour immune response^26,27^. In the original Sorin et al. study, Th and Tc populations showed stronger cancer-cell interactions in lower-grade tumours than in high-grade solid LUAD^25^. Therefore, SpaCEy specifically highlights these regions of cancer-immune cell interactions that are characteristic of tumours with better prognosis. Analysis of cell-type proportions revealed differences between progression and non-progression groups Fig. 2d which shows cell-type enrichment among important nodes. Tumours from patients who progressed showed areas with significantly increased frequencies of macrophage populations, including CD163^+^ macrophages, as well as higher cancer and endothelial cell content. This is consistent with the original publication demonstrating that CD163^+^ tumour-associated macrophages, typically linked to immunosupression and tumour progression, are enriched in high-grade tumours and strongly co-occur with FOXP3^+^ regulatory T cells^25^. The increased co-localisation and interaction between cancer and endothelial cells observed in higher-grade histological patterns reflects their aggressive nature and high metastatic potential^25^. To provide an image-level example, we selected a representative progressor LUAD sample and visualised direct graph contacts between explainer-important cancer and endothelial cell nodes, highlighted with bold edges (Fig. 2e). This example illustrates local cancer–endothelial adjacency within an explainer-highlighted tissue region. Interestingly, SpaCEy also identifies a previously not described enrichment of CD163^−^ macrophages in regions associated with progression. In contrast, non-progressing tumours exhibited areas with elevated numbers of Th cells, Tc cells and B cells. To assess whether this immune-cell signal was located in direct proximity to tumour cells, we defined tumour adjacency as a direct graph edge between an important immune cell and at least one cancer cell. Using this definition, 49.9% of pooled important Tc cells (3,019 of 6,053 cells) and 40.9% of pooled important Th cells (4,416 of 10,803 cells) in the no-progression group were cancer-adjacent, indicating that this no-progression-associated T-cell signal is partly tumour-localised. For comparison with the entire Tc and Th cell populations in the same no-progression group, 46.8% of all Tc cells (38,074 of 81,330 cells) and 38.6% of all Th cells (48,467 of 125,557 cells) in the no-progression group were cancer-adjacent. Thus, important Tc and Th cells were only modestly more tumour-adjacent than the corresponding global Tc/Th populations. CD4, CD8a, and FOXP3 are measured marker channels, whereas Th, Tc, Treg, and T other are source cell-type annotation labels shown in Fig. 2d. Tc and Th cells play key roles in anti-tumour immunity, whereas B cells have previously been associated with improved overall survival in LUAD^25^. Overall, these patterns indicate that SpaCEy identifies clinically relevant regions predictive of tumour progression and also shows potential to reveal emergent spatial trends.

We evaluated SpaCEy against two state-of-the-art baselines, Ali et al.^14^ and SPACE-GM^12^, using five-fold cross-validation across all performance metrics (accuracy, F1-score, and AUC) Fig. 2f. We performed a random split stratified by disease status and fixed the splits to ensure a fair comparison and an equal distribution of disease status across folds. SpaCEy achieved the highest overall performance and the most stable behaviour across folds. In terms of accuracy, SpaCEy reached a mean of 0.68, outperforming both Ali et al. (0.61) and SPACE-GM (0.55). Similar trends were observed for F1-score, where SpaCEy got 0.68 on average, whereas the method from Ali et al. and SPACE-GM achieved 0.59 and 0.52, respectively. SpaCEy also demonstrated higher discriminative capability, yielding the highest mean AUC (0.62), compared with 0.57 for Ali et al. and 0.52 for SPACE-GM. Notably, SpaCEy consistently performed strongly across folds, including peak values of 0.77 accuracy, 0.75 F1-score, and 0.78 AUC.

### SpaCEy stratifies breast cancer patients by subtype-agnostic localised spatial patterns

**Fig. 3:**
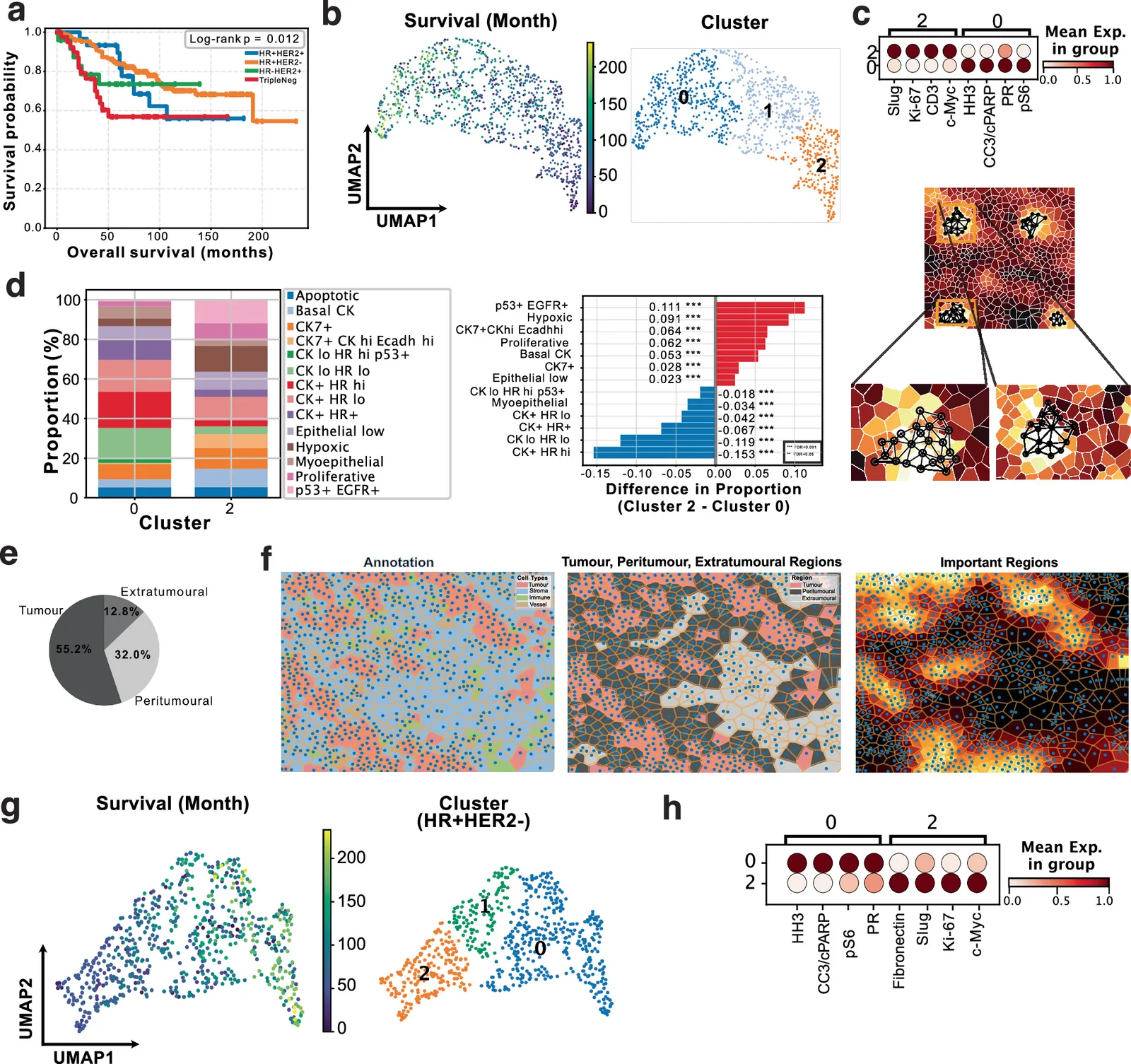
Survival-associated spatial patterns in the JacksonFischer breast cancer cohort. **a** Kaplan-Meier estimates of overall survival across clinical subtypes in the JacksonFischer cohort, with censoring marks shown on each curve and a global log-rank test **b** UMAP visualisation of sample embeddings derived from the trained model, showing overall survivability distributions across clusters and highlighting potential cluster-specific survivability trends. **c** Differential abundance analysis identifying distinct protein markers between clusters, with Cluster 0 representing high-survival patients and Cluster 2 representing low-survival patients. A representative lower-survival patient is shown alongside the analysis, illustrating overlap between regions identified as important by the model and local abundance of Slug and c-Myc proteins. **d** Cell type proportions of important nodes in the JacksonFischer dataset and proportional differences between Cluster 2 and Cluster 0. Positive values indicate enrichment in Cluster 2, while negative values indicate enrichment in Cluster 0, with enrichment scores shown for each cell type. **e** Distribution of important regions identified by the model. **f** Cell type annotations one representative sample: first column shows overall annotation, second highlights tumour core, peritumoural, and extratumoural regions, and third indicates important regions. **g** UMAP visualisation of latent representations for HR+HER2- patients, showing clustering structure and overall survivability distributions per cluster. **h** Differential expression analysis identifying distinct protein markers between clusters, with Cluster 0 corresponding to high-survival and Cluster 2 to low-survival patients. Source data are provided as a Source Data file.

The second dataset used to train SpaCEy was an imaging mass cytometry dataset comprising 720 samples from two patient cohorts at the University Hospital of Basel and the University Hospital of Zurich, which includes breast cancer samples across a range of clinical subtypes and cancer stages^28^. We refer to this dataset as the JacksonFischer dataset throughout the remainder of the text. Here, our objective was to model overall survival (hereafter referred to as survival) in a censoring-aware time-to-event setting. Summary statistics and cohort characteristics are provided in Supplementary Table 2. For the JacksonFischer dataset, Kaplan–Meier curves (Fig. 3a) stratified by clinical subtype (HR+HER2+, HR+HER2-, HR-HER2+, and TripleNeg) showed significant differences among clinical subtypes in overall survival (log-rank *p* = 0.012), where patients with triple-negative breast cancer (TNBC) exhibit the lowest survival rates due to the aggressive nature of the subtype, lack of targeted therapies, and higher likelihood of early metastasis^29,30^. The corresponding UMAP of the embeddings learned by the SpaCEy is shown in (Fig. 3b). The embeddings revealed a structured distribution of samples, with similarity grouping associated primarily with survival.

We then performed post hoc clustering after model training by applying the Leiden algorithm to the learned sample-level embeddings^31^. Based on the resulting clusters, patients were stratified into three subtype-agnostic groups exhibiting distinct survival distributions (Fig. 3b). Patient groups obtained by clustering of sample embeddings showed significant global survival separation in JacksonFischer dataset (global log-rank *p* = 0.00253; Supplementary Fig. 5). Clinical subtype composition was summarised only post hoc to contextualise these embedding-derived groups: Cluster 0 was enriched for high-survival patients with high variance, showing the highest proportion of HR+HER2+ samples (45.7%) and the lowest proportion of TNBC samples (22.7%) (Fig. 3b, Supplementary Fig. 1). In contrast, Cluster 2 was enriched for low-survival patients, showing the highest proportion of TNBC samples (41%) and the lowest proportion of HR+HER2+ samples (12.4%). Next, we isolated the important regions and examined differentially abundant proteins between Cluster 2, enriched for low-survival samples, and Cluster 0, enriched for high-survival samples. These regions are not restricted to a single biological compartment, but may include tumour, stromal, immune, and vascular cells. Next, we identified spatially contiguous protein expression patterns that distinguished high- and low-survival clusters in the overall dataset (Fig. 3c). In particular, proteins associated with high-survival patients (Cluster 0) differed from those in low-survival clusters (Clusters 2). The identification of localised expression of Slug, Ki-67, CD3, and c-Myc in lower survival patients from the overall dataset suggests their association with breast cancer progression. For example, Slug is a transcription factor involved in epithelial-to-mesenchymal transition (EMT), which enhances tumour invasiveness and metastasis^32^. Ki-67, a widely used proliferation marker, indicates aggressive tumour growth, and elevated Ki-67 levels are generally associated with worse survival in breast cancer^33,34^. Finally, c-Myc, an oncogene involved in cell cycle regulation and proliferation, is frequently overexpressed in aggressive breast tumours and is associated with poor prognosis^35^. The combination of these markers indicates a tumour biology characterised by high proliferation (Ki-67, c-Myc), EMT-driven invasion (Slug). To illustrate these marker level findings at the tissue level, we visualised one representative lower-survival sample from Cluster 2. SpaCEy highlighted spatially localised important regions within the tissue section, and zoomed views of these regions showed locally elevated Slug and c-Myc abundance within the model-selected subgraphs (Fig. 3c, bottom) which indicates that the associated marker signals are not only detected at the aggregate level, but are also spatially organised within important regions of individual tissue samples. Notably, detecting CD3 in lower survival patients in the overall dataset suggests heterogeneity in immune cell infiltration, with interpretation depending on whether CD3-positive cells are located in tumour or stromal important regions. To determine whether survival-associated marker signals were localised to specific tissue compartments rather than reflecting global tissue-wide changes, important nodes were first separated into broad cellular compartments, including tumour, stroma, immune, and vessel. Marker abundance was then summarised as the sample-level mean within each compartment (Supplementary Table 6). For example, a higher CD3 signal in stromal-context important nodes means that the ROI-level mean CD3 abundance among important nodes assigned to the stromal context is higher in lower-survival ROIs. This suggests the presence of stromal regions with a high abundance of CD3-enriched cells in patients with poorer survival, whereas such regions are largely absent in the better-survival group. Given the immune-excluded phenotype, therapeutic strategies aimed at stromal remodelling or reprogramming, such as combination therapy with anti-TGF-*β* agents, or at normalisation of the tumour vasculature may promote T-cell infiltration into the tumour and potentially improve survival in patients with poor prognosis^36^. In the JacksonFischer cohort, Ki-67 had its strongest lower survival shift in tumour-context important nodes, whereas CD3, Slug, and c-Myc had their strongest lower survival shifts in stromal-context important nodes. For each marker, the reported compartment corresponds to the context with the largest positive standardised mean difference between lower- and higher-survival samples. We therefore interpret these markers as compartment-resolved signals within important regions rather than as global tissue-wide marker changes or direct evidence of cell-intrinsic mechanisms.

To further dissect cellular composition within these key clusters, we performed cell type proportion analysis and identified the highest proportional differences based on the nodes identified as important in the JacksonFischer dataset (Fig. 3d. Specifically, Cluster 0 exhibited important nodes with a higher abundance of hormone receptor-positive cells known to respond to endocrine therapy and, which may contribute to favourable survival^37^. In contrast, Cluster 2 was enriched with p53+ EGFR-positive, hypoxic, basal, and highly proliferative cancer cell phenotypes. Breast tumours with alteration in TP53 have a worse prognosis^38^, and EGFR overactivation drives proliferation and survival signalling^39^, and has been associated with poor clinical outcomes^40^. Both hypoxia and high proliferation indicate rapid tumour growth and aggressive clinical behaviour^41,42^. Basal cells, also known as myoepithelial cells, express markers of EMT and contribute to invasive behaviour^43^. Collectively, areas with a higher proportion of cells exhibiting upregulation of oncogenic proteins, signatures and processes linked to rapid tumour growth, metastasis and therapy resistance is expected in the poor survival group. Similar associations between phenotypic composition and patient outcomes have also been reported in previous studies^28,44^. We further characterised the localisation of important regions within the tissue (Fig. 3e). We found that 55.2% of these regions were in tumour areas suggesting that the abundance values in the tumour areas are the most informative for the survival prediction. The second most important regions are the peritumoural areas suggesting that tumour microenvironment plays a critical role in disease progression and patient outcomes. For example, hypoxia in the tumour microenvironment can lead to genetic instability and more aggressive tumour phenotypes. In breast cancer specifically, hypoxic regions are linked to metastasis, treatment resistance, and poor prognosis and these factors may contribute to the observed spatial distribution of important regions and their association with patient survival^45,46^. An example case is given in Fig. 3f where these important regions predominantly align with the tumour core and peritumoural areas. Finally, we assessed the composition of important nodes across the JacksonFischer dataset and found that they were distributed across multiple tissue compartments, with mean proportions of 55.2% tumour, 29.3% stroma, 13.5% immune, and 2.0% vascular compartments. This indicates that identified regions by the explainer are not confined to a single compartment, but instead capture outcome-associated local tissue structures spanning mainly tumour and stromal contexts, with additional immune and vascular contributions. Given this mixed composition, we focused on the stromal compartment by quantifying the cellular composition of important nodes at the sample level. To directly assess whether stromal-associated marker signals were driven by a higher stromal fraction among important nodes, we compared proportions of important nodes per compartment between lower- and higher-survival groups. In JacksonFischer, lower-survival samples showed a lower stromal fraction of important nodes than higher-survival samples (27.7% vs. 30.9%; Mann–Whitney *p* = 0.018), indicating that stromal marker enrichment should not be interpreted as evidence that a larger stromal area alone explains the lower-survival phenotype. Rather, these markers represent marker-level signals within heterogeneous important regions, which we further interpret using context-stratified marker summaries where annotations are available.

### SpaCEy explains the heterogeneity of the survival of patients within a clinical subtype

Hormone receptor-positive, HER2-negative (HR+HER2-) breast cancer represents the most prevalent molecular subtype, accounting for the majority of breast cancer diagnoses. Although patients with HR+HER2- tumours generally exhibit more favourable prognoses compared to more aggressive subtypes such as TNBC, substantial heterogeneity remains in disease progression, therapeutic response, and overall clinical outcomes^47^. This variability presents a significant challenge for precision oncology, as current classification systems may not adequately capture the biologically relevant distinctions within this subgroup. To address this gap, we investigated the latent molecular architecture of HR+HER2- tumours using spatially resolved proteomic data. As shown in the UMAP embedding, patient samples with similar survival tend to cluster together, specifically, reduced survival in cluster 2 and higher survival values in cluster 0 in Fig. 3g. Notably, samples within HR+HER2- subtype show varying survival, underscoring the biological heterogeneity that exists within this molecular subtype and can be group into three clusters. Kaplan-Meier curves for the clusters in HR+HER2- clinical subtype (global log-rank *p* = 0.03132) are shown in Supplementary Fig. 7.

Beyond patient re-stratification, SpaCEy allows for improved insights into previously well-established clinical subtypes and heterogeneity of outcome. When comparing explainer features between the entire dataset and the HR+HER2-subgroup (Fig. 3h), we observed a significant overlap in the survival-associated proteins. To test whether the overlap between HR+HER2- and the full cohort was driven by the prevalence of HR+HER2- samples, we performed a targeted subtype analysis restricted to the identified markers associated with lower survival patients: Slug, c-Myc, CD3, and Fibronectin. For each clinical subtype and for the combined non-HR+HER2- group, patients were split by the group-specific median overall survival. Marker abundance was then summarised as the patient-level mean abundance among hard-mask SpaCEy-important nodes (Supplementary Table 7). All four reported markers showed positive lower-survival-associated shifts in both the full cohort and the HR+HER2- subgroup. Importantly, the same lower-survival-associated trend was also observed in the combined non-HR+HER2- group (Supplementary Data 3). In this group, Slug, c-Myc, and CD3 were FDR-supported, while Fibronectin showed the same direction of change but was not statistically significant. These results indicate that the marker overlap is not solely explained by HR+HER2- dominance. Rather, SpaCEy captures a shared lower-survival-associated component across receptor-defined subtypes while retaining subtype-specific variation. The high expression of Ki-67, Slug, and c-Myc in the lower-survival samples of both the whole data set and HR+HER2- subset highlights the central role of these proteins in tumour proliferation and progression in breast cancer in general. The elevated fibronectin signal in important regions from lower-survival HR+HER2- patients is consistent with prior METABRIC-based evidence linking high fibronectin expression in primary breast tumours to decreased patient survival, while also pointing to context-dependent variation in epithelial-mesenchymal state and metastatic potential^48^. More broadly, we observed that high expression of apoptotic markers, such as cleaved caspase and PARP, was enriched in the high survival group. The spatial expression patterns of these proteins enhance our comprehension of the biological diversity within HR+HER2- breast cancer, and can be potentially used as a guide to personalised treatment strategies.

### SpaCEy generates generalisable insights across cohorts

**Fig. 4:**
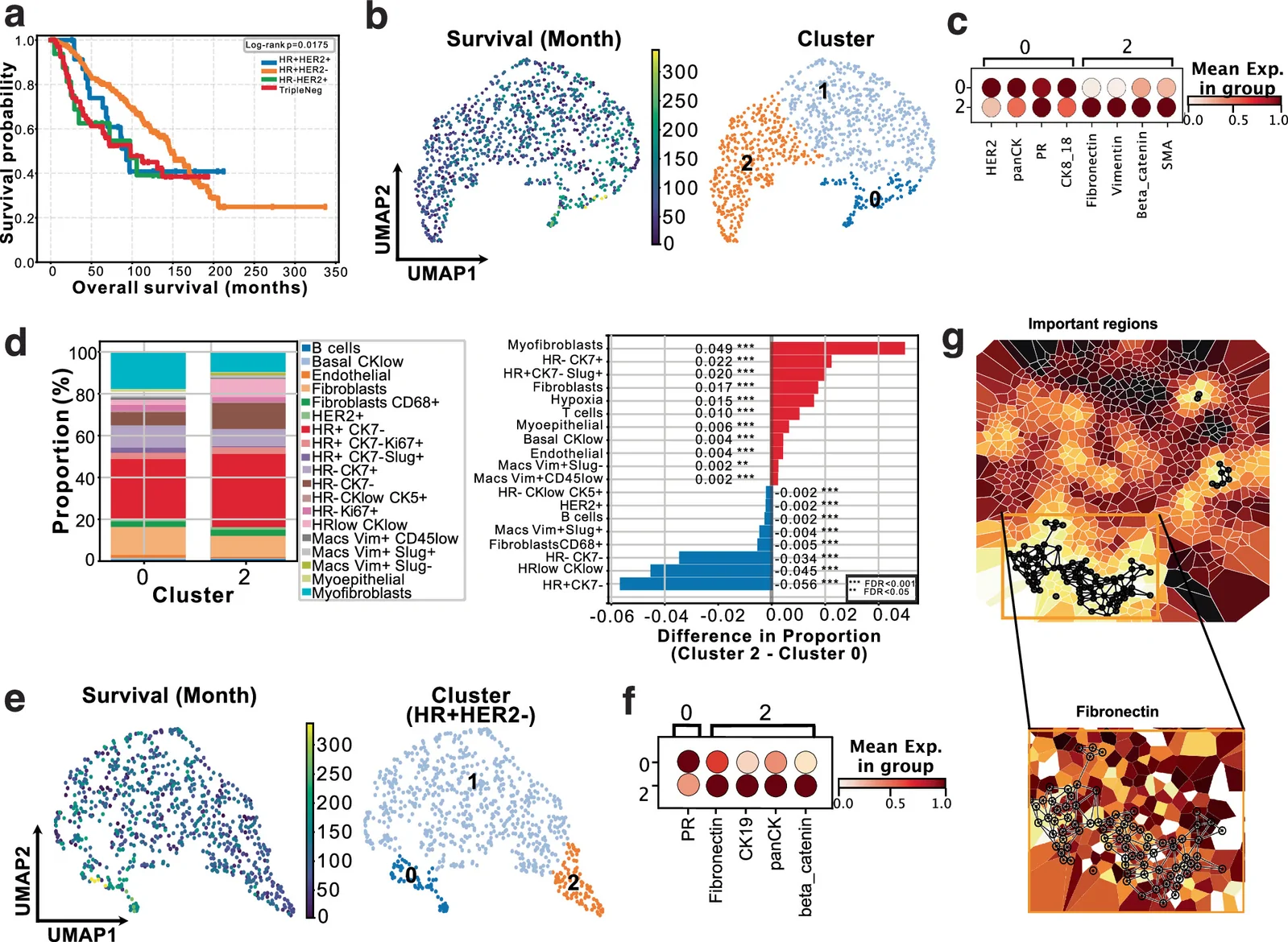
Survival-associated spatial patterns in the METABRIC imaging cohort. **a** Kaplan--Meier estimates of overall survival across clinical subtypes in the METABRIC imaging cohort, with censoring marks shown on each curve and a global log-rank test. **b** UMAP visualisation of patient samples coloured by survival values and clustering results along with the distribution of survival values across different clusters. **c** Marker expression profiles across lower- and higher survival clusters. **d** Cell type proportions of important nodes in the METABRIC dataset and cell type proportion differences between Cluster 2 and Cluster 0. **e** UMAP visualisation of HR+HER2- samples coloured by survival values and clusters and distribution of survival values across different clusters in HR+HER- samples **f** Differential expression analysis identifies distinct protein markers between clusters, with Cluster 0 representing high-survival patients and Cluster 2 representing low-survival patients in the dataset. **g** Representative METABRIC sample showing important regions and the corresponding spatial distribution of Fibronectin. The upper panel shows node importance across the sample, with explainer-highlighted nodes and their spatial graph connections overlaid. The boxed region is enlarged in the lower panel, showing Fibronectin abundance. Source data are provided as a Source Data file.

We used the METABRIC dataset as a second independent breast cancer cohort to train a separate model^49^. This allowed us to further investigate the relationship between patient survival and spatially resolved proteomic features. The METABRIC dataset comprises 460 imaging mass cytometry samples from 405 patients, capturing diverse clinical features. The preprocessing steps are described in the Data and Preprocessing subsection, and summary statistics of the METABRIC dataset are presented in Supplementary Table 3. For the METABRIC imaging cohort, Kaplan–Meier curves stratified by clinical subtype (HR+HER2+, HR+HER2-, HR-HER2+, and TripleNeg) also showed significant differences among clinical subtypes in overall survival (log-rank *p* = 0.0175). Similar to our previous observations, UMAP embeddings of the METABRIC dataset (Fig. 4b) demonstrated relevant patterns associated with overall survivability, with Cluster 0 being predominantly associated with high survival and Cluster 2 with lower survival. Patient groups obtained by clustering of the sample embeddings showed significant global survival separation in METABRIC dataset (global log-rank *p* = 0.00371; Supplementary Fig. 5). Differential protein abundance analysis identified distinct molecular markers distinguishing these survival-associated clusters (Fig. 4c). In Cluster 0 (higher survival), we observed enrichment of HER2 and CK8/18. Conversely, Cluster 2 (lower survival) was characterised by increased expression of fibronectin, vimentin, beta-catenin, and SMA. Because METABRIC contains a higher fraction of HR+HER2- patients than JacksonFischer, we performed a descriptive subtype-composition analysis. HR+HER2- patients accounted for 71% of METABRIC patients versus 63% of JacksonFischer patients. Within METABRIC, fibronectin abundance in important nodes was not enriched in HR+HER2- patients relative to other subtypes (mean 5.62 vs. 6.34; Mann–Whitney U test, *p* = 0.84). We therefore interpret fibronectin as an important feature associated with the lower-survival group in METABRIC dataset, rather than as a standalone prognostic biomarker uniformly conserved across cohorts. This observation is consistent with the clinical characteristics of patients within this cluster^50^. Notably, PR and PanCK were highly expressed across the two clusters, with PR showing relatively higher abundance in the higher survival group, suggesting a shared molecular signature among patient subgroups. The occurrence of these patterns across datasets indicates that the SpaCEy captures underlying biological signals.

To further dissect the cellular composition underlying these clusters, we compared the proportions of cell types across clusters and calculated significant cell type proportional differences between clusters (Fig. 4d). As in JacksonFischer dataset, we observed areas with a higher proportion of hormone receptor-positive cells in Cluster 0, while hypoxic and myoepithelial/basal were more abundant in Cluster 2, which is associated with worse survival outcomes. The METABRIC dataset also allowed analysis of tumour microenvironment and revealed areas with higher proportion of myofibroblasts and fibroblasts in patients with lower survival, demonstrating the role of fibrotic stroma and cancer-associated fibroblasts in promoting malignant growth and tumour invasion^51^. Across METABRIC samples, important nodes were also compositionally mixed, with mean important-node proportions of 58.0% tumour, 30.4% stroma, 9.0% immune, 1.6% vessel, and 1.0% unclassified regions. Similarly, in METABRIC, stromal important-node fractions were comparable between lower- and higher-survival ROIs (30.0% vs 30.8%; *p* = 0.263). Focusing on the HR+HER2- clinical subtype, the visualisation of the learned latent representations in UMAP space (Fig. 4e) reveals clustering of samples according to patients’ overall survival profiles. Kaplan-Meier curves for the clusters in HR+HER2- clinical subtype (global log-rank *p* = 0.01498) are shown in Supplementary Fig. 7. The distribution of overall survival across these clusters is consistent with our previous observations, with Cluster 2 enriched for patients with shorter survival and Cluster 0 comprising those with longer survival times. Next, we explored differential protein abundance across HR+HER2- patient clusters (Fig. 4f). In Cluster 0 (higher survival), we observed higher localised expression of PR while Cluster 2 (lower survival) showed an enrichment of fibronectin, *β*-catenin and CK19. Higher abundance of fibronectin in the lower survival group is consistent with the overall analysis above. Increased *β*-catenin expression in breast cancer is significantly associated with poor prognosis, as demonstrated by a study where patients exhibiting nuclear and/or cytoplasmic *β*-catenin expression had reduced disease-specific survival rates^52^. The association between PR (progesterone receptor) expression and favourable prognosis in hormone receptor-positive breast cancer is widely acknowledged. A meta-analysis demonstrated that patients with high PR expression had significantly better survival compared to those with low PR expression^53^. These results highlight partially overlapping spatial and molecular patterns in the METABRIC and JacksonFischer datasets, reinforcing the association between protein abundance, tumour cellular composition and patient survival outcomes, and demonstrating SpaCEy’s ability to consistently identify prognostic patterns.

### SpaCEy improves prediction performance using spatially resolved data

**Fig. 5:**
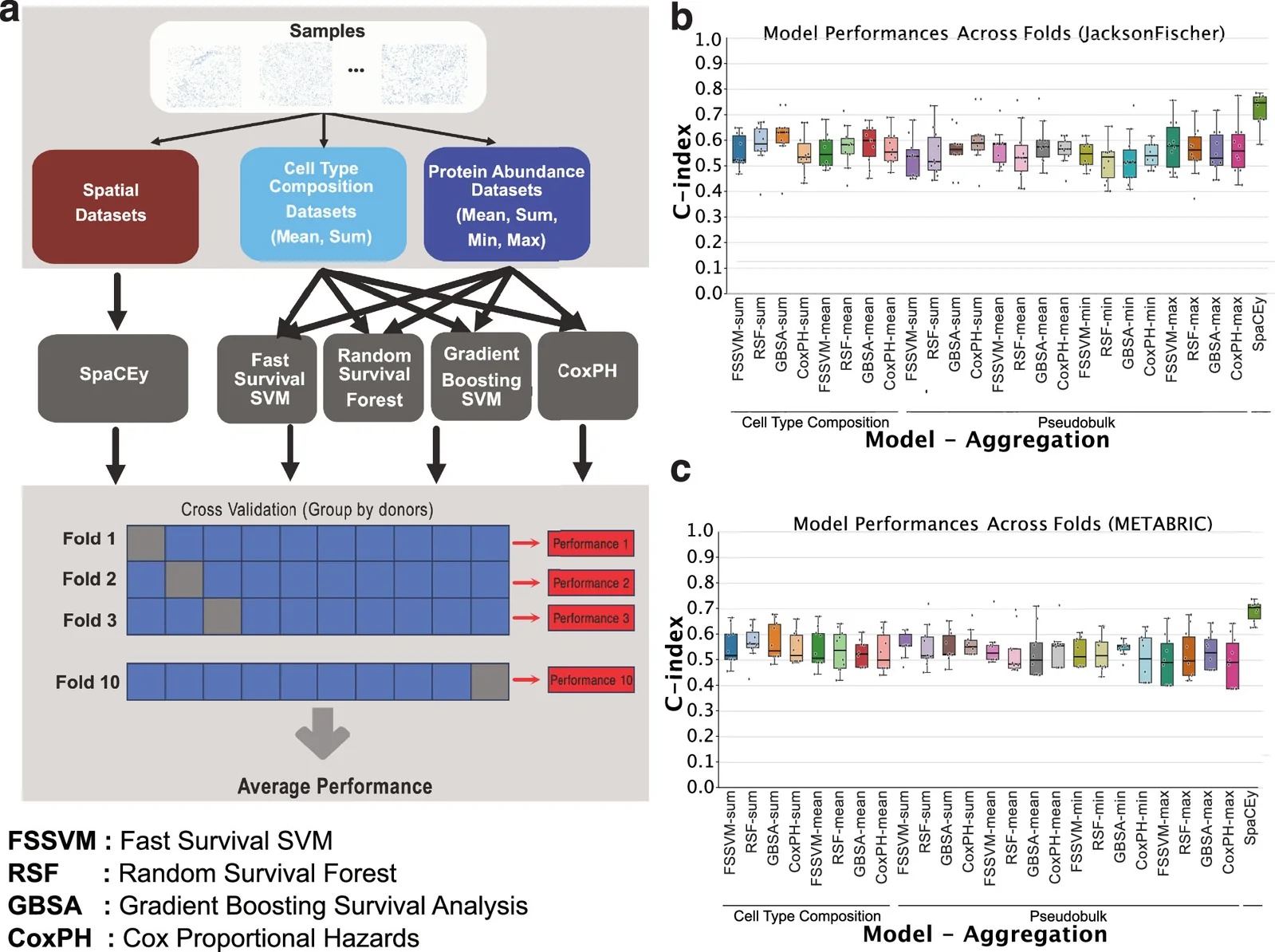
Comparison with non-spatial baseline methods. **a** Baseline comparison pipeline. **b** Performance comparison on JacksonFischer Dataset. Comparison of test-set concordance indices for SpaCEy and non-spatial survival baselines using pseudobulk marker summaries and cell-type-composition features. Each box summarises *n* = 10 fixed patient-separated train/test splits. The centre line indicates the median; box limits indicate the 25th and 75th percentiles; whiskers extend to the smallest and largest observations within 1.5 times the interquartile range; and values outside the whiskers are shown as individual points. **c** Similar performance comparison on METABRIC Dataset. Source data are provided as a Source Data file.

We also benchmarked SpaCEy against other machine-learning methods that do not incorporate spatial information, using the JacksonFischer and METABRIC datasets. This comparison enabled a direct assessment of whether spatial context improves predictive performance. To this end, we compared our results with four related methods: Fast Survival SVM^9^, Random Survival Forest^10^, Gradient Boosting Survival Analysis^11^, and a Cox proportional hazards (CoxPH) model^54^. To perform this comparison, we generated pseudobulk profiles of the samples based on protein abundance values and cell type annotations. For protein abundance, we applied four aggregation functions (minimum, maximum, mean, and sum) resulting in four distinct training datasets. For cell type-based aggregation, we created two additional training datasets using mean and sum aggregators (Fig. 5a).

We performed a comprehensive hyperparameter optimisation for each method-aggregation combination (Supplementary Data 1), optimising a total of 24 models. To ensure methodological rigor and a fair comparison across models, we employed consistent cross-validation splits in which folds were stratified at the patient level to prevent data leakage. The results for the JacksonFischer and METABRIC datasets are presented in Fig. 5b, c, respectively. Model performance was evaluated using the concordance index (C-index) as the primary metric. The choice of aggregation functions was motivated by their ability to capture different aspects of the protein abundance values, while mean aggregation smooths out individual variations, maximum and minimum values preserve extreme signals that might be biologically relevant. Sum aggregation, on the other hand, retains total protein abundance levels, which could be important for capturing global trends in tumour heterogeneity.

JacksonFischer dataset, SpaCEy achieved a mean performance of 0.720 ± 0.061, outperforming all 24 non-spatial baseline configurations evaluated through the same fixed 10-fold protocol. The best-performing baseline method was Cell Type Composition with Gradient Boosting SurvivalAnalysis using sum aggregation, which achieved 0.617 ± 0.092. For the Cox proportional hazards (CoxPH) baseline, the best-performing variant used pseudobulk marker summaries with sum aggregation and achieved a mean test C-index of 0.600 ± 0.095. SpaCEy demonstrated a substantial improvement of 0.102, representing a 16.5% relative improvement over this best baseline method.

On the METABRIC dataset, SpaCEy achieved a mean performance of 0.688 ± 0.038, again significantly outperforming all baseline methods. The best-performing baseline method was Cell Type Composition with Gradient Boosting Survival Analysis using sum aggregation, which achieved 0.570 ± 0.071. For the CoxPH baseline, the best-performing variant again used pseudobulk marker summaries with sum aggregation and achieved a mean test C-index of 0.562 ± 0.048. SpaCEy demonstrated an even larger improvement of 0.118, representing a 20.7% relative improvement over this best baseline method. The lower standard deviation of SpaCEy’s performance compared to baseline methods indicates more stable and reliable predictive capability across both datasets.

Importantly, our proposed model demonstrated the highest and most robust performance, with a clear improvement in median C-index compared to all alternative methods in both datasets. This gain was particularly evident in the METABRIC cohort, where traditional models showed more variable performance. These results indicate that our model generalises well across cohorts and outperforms existing approaches by leveraging aggregated cellular features more effectively.

To compare SpaCEy with a supervised non-spatial representation in terms of embedding-level stratification, we trained a non-spatial multilayer perceptron (MLP) baseline using pseudobulk marker features from the same samples and the same censoring-aware Cox loss used for SpaCEy. We extracted sample-level MLP embeddings, clustered them into three groups, and evaluated patient-level survival using Kaplan–Meier curves and a global log-rank test across all clusters. The non-spatial MLP sample-embedding baseline did not show significant global separation in JacksonFischer (global log-rank *p* = 0.23210) or METABRIC (global log-rank *p* = 0.12239; Supplementary Fig. 6). We therefore use these cluster-level survival analyses as descriptive summaries of supervised representations, not as independent validation of predictive performance.

### SpaCEy learns predictive spatial representations from CODEX data and retains signal across batches

To evaluate SpaCEy representations under batch variation, we performed a batch-focused analysis using a colorectal cancer cohort, measured with CODEX, comprising 140 tissue regions from 35 patients^55^. This dataset provides a same-panel setting in which cross-batch transfer can be assessed directly, avoiding the marker-panel incompatibility that limits comparisons across some spatial proteomics datasets. We used this dataset for two related purposes: first, to test whether SpaCEy can learn predictive spatial representations from CODEX data, and second, to evaluate its sensitivity to batch effects under a controlled same-panel transfer setting. We selected the CODEX colorectal cohort because matched batch samples share the same marker panel, enabling a controlled same-panel cross-batch test. A direct JacksonFischer-to-METABRIC breast cancer transfer was not used for this analysis because marker-panel mismatch (Supplementary Data 4).

The prediction task was formulated as a supervised binary classification problem using immune phenotype annotations, distinguishing Crohn’s-like reaction (CLR) from diffuse inflammatory infiltration (DII). We used a patient-stratified 80/10/10 train/validation/test split, ensuring that no patient appeared in more than one split. After hyperparameter optimisation, we repeated the selected model configuration across five random seeds while keeping the patient-level split fixed. Across these five runs, SpaCEy achieved a held-out test AUPRC of 0.846  ±0.026. These results indicate that SpaCEy can be trained on an independent CODEX multiplex imaging cohort while preserving patient-level separation between training, validation, and test sets.

We next assessed batch sensitivity using the matched tissue microarray batches A and B from the same CODEX cohort. Because each patient was represented in both batches, a naive batch holdout would leak patient information across splits. We therefore separated train/validation and test partitions at the patient level. Specifically, the model was trained and validated on batch A and evaluated on held-out samples from different patients in batch B. This A-to-B transfer setting is more stringent than the randomly patient-stratified analysis above, because the model is exposed during training only to the source batch and must generalise to samples acquired in a different batch.

SpaCEy retained predictive performance on held-out batch B in this cross-batch setting, achieving an AUPRC of 0.801 ± 0.016 across random seeds. As expected, performance on held-out batch B was slightly lower than in the randomly patient-stratified analysis, consistent with the additional challenge imposed by batch transfer. To provide direct visual evidence for this A-to-B transfer setting, we visualised first fully connected layer sample-level embeddings from the cross-batch model using UMAP, with the same samples coloured by technical batch and by CLR/DII immune phenotype (Supplementary Fig. 4). The embeddings did not show strong separation by technical batch alone, with a technical-batch silhouette score near zero for A-to-B (0.0009), supporting the conclusion that the representation was not dominated solely by batch identity.

### Scalability analysis results

**Fig. 6:**
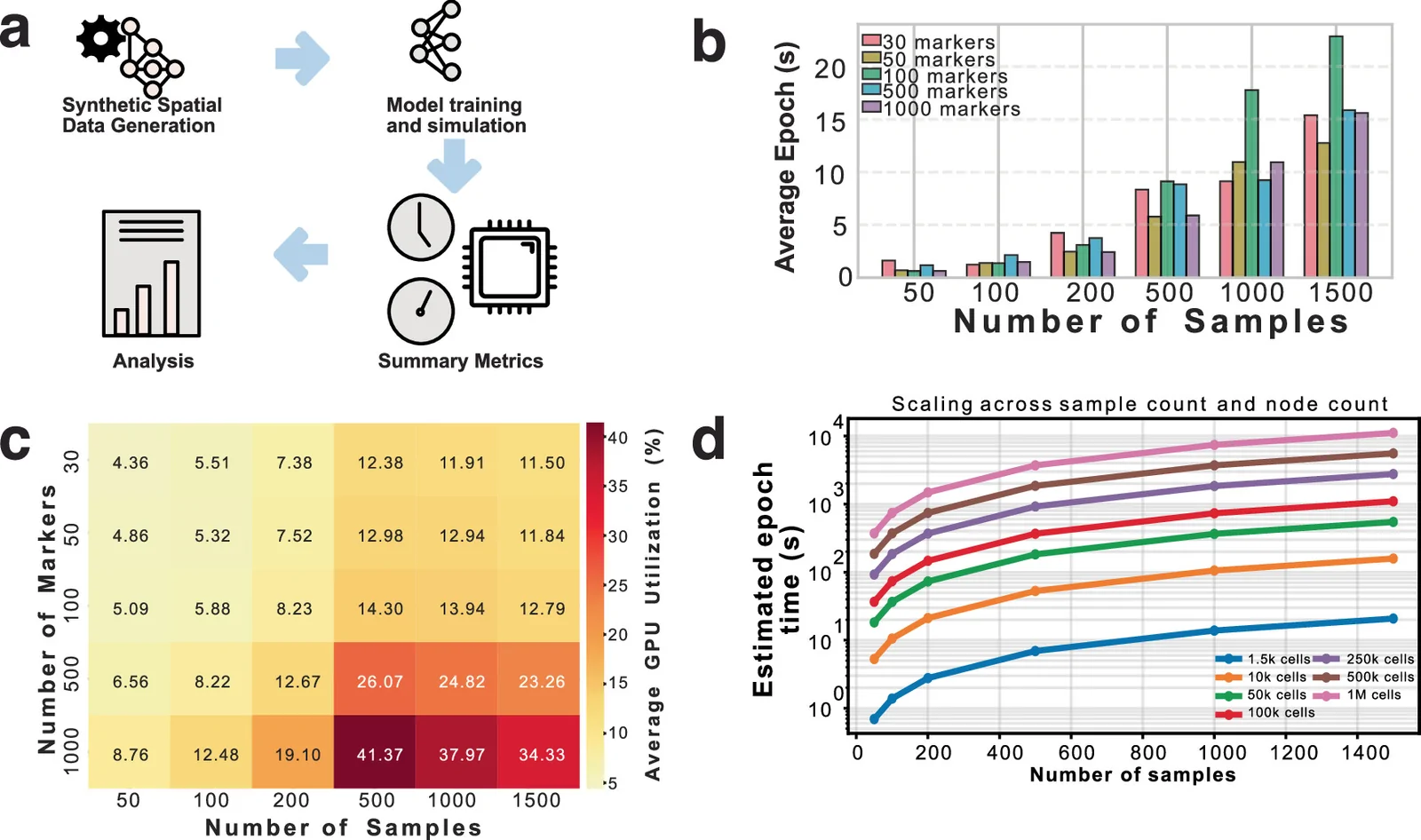
Computational performance and scalability analysis. **a** Overview of the computational pipeline comprising synthetic spatial data generation, model training and simulation, computation of summary metrics, and downstream analysis. **b** Average epoch time (in seconds) as a function of the number of samples and markers, showing the scaling behaviour of training time with increasing dataset size. **c** Heatmap of average GPU memory utilisation (%) across varying numbers of samples and markers, highlighting resource demands for large-scale simulations. **d** Scaling of average epoch time as a function of sample number for different graph sizes, illustrating the expected increase in runtime with both the number of samples and the number of cells per sample. Source data are provided as a Source Data file.

To ensure our model remains computationally practical for large-scale spatial datasets, we assessed the computational scalability of the model across 30 configurations varying in both marker dimensionality and sample size (Fig. 6). The benchmarking workflow comprised four main stages: synthetic spatial data generation, model training and simulation, computation of performance metrics, and downstream analysis (Fig. 6a). This systematic analysis enabled quantitative evaluation of runtime and GPU memory usage under diverse experimental conditions. The complete results of scalibility analysis are given in Supplementary Data 2.

Model optimisation was performed over multiple epochs, where one epoch corresponds to a complete pass of the model through the entire training dataset. Each epoch therefore represents a single iteration of parameter updates across all samples. Average epoch time ranged from 0.63 to 22.86 s (mean = 6.89 ± 6.12 s), highlighting favourable performance across all tested conditions (Fig. 6b). For small datasets (50 samples), all marker configurations exhibited comparable performance (0.95  ± 0.43 s per epoch), enabling rapid model prototyping. Epoch time increased approximately linearly with dataset size, reaching 7.59 ± 1.63 s at 500 samples, 11.60 ± 3.56 s at 1000 samples, and 16.50 ± 3.77 s at 1500 samples. Interestingly, a non-monotonic trend was observed between marker count and training time, with intermediate dimensionality (100 markers) resulting in slower performance at large sample sizes (20.32 s vs. 12.48 s for other configurations with ≥1000 samples), potentially due to suboptimal GPU memory access patterns. Overall, the model exhibited sub-linear scaling behaviour, with per-sample processing time decreasing at larger batch sizes, suggesting improved computational efficiency for large-scale, population-level analyses.

GPU memory utilisation remained modest across all configurations, ranging from 4.36 to 41.37% (mean = 14.14 ± 9.90%), with a maximum absolute memory usage of only 235.18 MB for the largest configuration (1000 markers, 500 samples) (Fig. 6c). Memory consumption increased primarily with marker dimensionality (30 markers: 8.84%; 1000 markers: 25.67%) rather than sample size, reflecting the feature-centric architecture of the model. Notably, memory efficiency improved by 9.7-fold at larger batch sizes (0.072 MB/sample at 1500 samples vs. 0.701 MB/sample at 50 samples), confirming that the model scales favourably in both compute time and memory usage.

To address whole-slide-scale settings directly, we updated the scalability benchmark to vary both the number of cells per sample and the number of samples. One synthetic sample was represented as a spatial point cloud (cells) with 1500, 10,000, 50,000, 100,000, 250,000, 500,000, or 1,000,000 cells and 50 marker channels (Fig. 6d, e). Samples exceeding a specified cell-count threshold were partitioned into cell tiles and processed; the learned features from these tiles were then aggregated to generate the final sample-level representation. The calculated per-sample processing time increased from 0.014 s for 1500-cell samples to 7.42 s for 1,000,000-cell samples. Scaling these measured per-sample times to epochs containing 50, 100, 200, 500, 1000, or 1500 samples gave estimated epoch times from 0.69 s for 50 samples with 1500 cells each to approximately 3 h for 1500 samples with 1,000,000 cells each. The largest setting, therefore, corresponds to 1.5 billion cells per epoch. Peak allocated GPU memory remained bounded by the tile size, reaching approximately 1.06 GB, while peak resident CPU memory was approximately 1.45 GB.

### Ablation and sensitivity analyses

To assess the contribution of individual SpaCEy components and evaluate the sensitivity and robustness of the model, we performed a series of ablation studies focusing on key architectural and input-design choices. To assess the sensitivity of SpaCEy to partial field of view, we performed an inference-only crop-sensitivity analysis on the JacksonFischer dataset using the trained survival model. Each full region of interests (ROIs) was compared against five fixed 50% crops defined from the ROI bounding box (centre, upper-left, upper-right, lower-left, and lower-right). Across full ROIs and 1578 valid crop comparisons, crop-based predictions remained highly concordant with full-ROI predictions (mean Spearman *ρ* = 0.943), while risk-tertile agreement remained 0.823 on average. Here, risk-tertile agreement means that the crop and its parent full ROI were assigned to the same low/mid/high risk tertile using cutpoints defined from the full-ROI risk-score distribution. Crop embeddings also remained highly similar to the corresponding full-ROI embeddings (mean cosine similarity 0.920). We further assessed whether crop-derived embeddings preserved the latent-space cluster assignment of the corresponding full ROI. In this analysis, full-ROI embeddings were clustered globally with Leiden, and each crop embedding was assigned to the nearest full-ROI cluster centroid by cosine similarity with the aim of measuring whether the crop remains in the same full-ROI embedding cluster as its parent ROI. On a stratified explanation subset of full ROIs and valid crop explanations, the cell-type composition of important nodes was stable (mean Pearson correlation 0.83–0.97). The cell-type composition correlation was calculated by restricting the full-ROI explanation to cells present in the crop, converting the important nodes from the restricted full ROI and crop explanation into normalised cell-type proportion vectors and computing the Pearson correlation between the two vectors. These results indicate that SpaCEy is moderately robust to partial observation, but ROI restriction still remains an important limitation for both prediction and interpretation. The crop-level prediction, embedding, and explanation-stability summaries are shown in Supplementary Table 5.

In a second ablation study, we asked whether explicit removal of identified important regions alters SpaCEy predictions and survival-ranking performance more strongly than removal of matched non-important regions. In the JacksonFischer cohort, we progressively removed 1%, 5%, 10%, 20%, 50%, and 80% of important nodes, together with their incident edges, and compared the performance of the trained model. Across all ablation levels, removal of important nodes produced consistently larger decreases in model performance than removal of non-important nodes and led to a stronger degradation in concordance index, indicating that explainer-identified regions had a greater effect on both individual risk estimates and survival-ranking performance. Specifically, mean decreases in model output were 0.0094 versus 0.0071 at 1%, 0.0509 versus 0.0179 at 5%, 0.0818 versus 0.0281 at 10%, 0.1034 versus 0.0455 at 20%, 0.1553 versus 0.0913 at 50%, and 0.2175 versus 0.1289 at 80% for important-node versus matched non-important-node removal, respectively. This consistent monotonic ablation pattern indicates that explainer-identified regions are more important for SpaCEy predictions than matched non-important regions, supporting the functional relevance of the highlighted nodes.

The aim of the third ablation study was to validate the explainer model in a setting with a known ground-truth spatial signal. For this, we generated a synthetic, standalone toy tumour-stroma dataset in which positive samples contained high intratumoural CD8 abundance, whereas negative samples had matched CD8 abundance shifted to the stromal compartment. The model received only synthetic marker-channel features and spatial graphs as input; the synthetic marker channels represented tumour, CD8, stromal, immune-activation, proliferation, fibroblast/SMA-like, and noise-control signals. Simulated cell-type labels (tumour cells, CD8 T cells, fibroblasts, and macrophage-like cells) and compartment labels were used only for ground-truth evaluation and post hoc interpretation, not for model prediction. The final synthetic dataset comprised 300 graphs with varying numbers of cells per graph, and a model was trained to predict the corresponding graph-level labels. When explainer model was applied to held-out test graphs, intra-tumoral CD8 nodes received higher mean importance than stromal CD8 nodes in explained graphs, with mean group importance 0.0467 for intra-tumoral CD8 versus 0.0162 for stromal CD8 (paired one-sided Wilcoxon signed-rank *p* = 8.29 × 10^−14^), supporting that the explainer selectively highlights the intended intra-tumoral CD8 spatial motif rather than extra-tumoral CD8 cells. Representative synthetic samples further showed that high-importance regions localised to intra-tumoral CD8 cells in positive graphs, while stromal CD8 cells outside the tumour compartment received lower importance scores (Supplementary Fig. 3).

## Discussion

SpaCEy introduces an explainable graph neural network method for spatial omics analysis that identifies tissue regions that are linked to clinical outcomes. Although graph neural networks have shown considerable success in biomedical prediction tasks, most existing methods largely lack mechanisms to capture spatially organised tissue patterns or to isolate a concise set of informative markers. SpaCEy addresses this gap through an integrated explainer module that highlights the contiguous spatial regions and molecular features most responsible for predictions. The resulting explanations provide interpretable representations of the tumour microenvironment, revealing patterns that distinguish patients with different outcomes. This capability moves beyond pure prediction, offering a way to generate hypotheses about how tissue structure and cellular interactions influence disease progression and patient survivability. In summary, SpaCEy enables patient stratification through analysis of learned representations while simultaneously translating these insights into spatially resolved marker profiles by capturing organisational spatial patterns of a focused set of protein markers.

We evaluated SpaCEy across three datasets and two different predictive settings. First, we applied it to a lung adenocarcinoma (LUAD) imaging mass cytometry dataset in a classification setting to predict tumour progression. Second, we trained our method on two breast cancer datasets, where SpaCEy identified survival-associated motifs while also capturing dataset-specific differences. These results indicate that it can detect both generalisable biological signals and context-dependent variations. Although demonstrated here on lung and breast cancer cohorts, the design of SpaCEy is broadly applicable and can be extended to other disease contexts. Importantly, its ability to link structural tissue motifs to clinical outcomes highlights the potential of SpaCEy as a discovery tool across a wide range of diseases.

We compared our method with two state-of-the-art spatially aware methods, and we showed that it achieved better performance on different metrics while identifying spatially important regions and markers^12,14^. We also compared SpaCEy with well-established non-spatial methods to assess its relative performance and to demonstrate that explicitly incorporating spatial information is essential for performance improvement and for capturing biologically meaningful spatial structure^9–11^. We demonstrated a clear performance improvement over the non-spatial methods, which often rely on aggregated features that obscure spatial context, limiting their ability to capture cell-cell dependencies. At the same time, unlike many graph-based methods that prioritise predictive accuracy at the expense of transparency, SpaCEy balances performance with explainability. This combination is particularly valuable in biomedical research, where the interpretability of machine learning models is critical for facilitating translation into clinical settings.

This study also has limitations. The current study focuses on multiplex imaging-based single-cell spatial proteomics, in which node features are protein-marker abundance measurements, and therefore provides only a partial view of cellular states and interactions. As broader multimodal datasets such as transcriptomic or metabolomic measurements become more widely accessible, and spatial technologies scale to larger tissue sections and higher molecular resolution, computational efficiency will become increasingly important. Future developments of SpaCEy will therefore incorporate strategies for scalable graph training to better accommodate the rapidly growing size and increasing complexity of multi-modal spatial datasets. Additionally, the quality of explanations is tied to the performance of the predictive models; therefore, explanations based on modest-performing models should be interpreted conservatively. Our sensitivity analysis showed that SpaCEy predictions, embeddings, and cell type compositions were largely stable under partial field-of-view sampling, but also confirmed that ROI restriction remains a relevant limitation because local crops may not fully capture the tissue context available in the complete ROI. Therefore, with the increasing availability of whole-slide spatial datasets linked to clinical annotations, this limitation can be assessed more comprehensively in future work. To assess whether this direction is computationally feasible, we also performed a scalability analysis of SpaCEy. This analysis showed that SpaCEy scales favourably with increasing sample size and marker dimensionality, supporting its potential application to larger spatial datasets, including whole-slide imaging-derived graphs. Moreover, similar to most feature-importance based interpretability methods, SpaCEy identifies features and spatial motifs that are important for prediction, not necessarily causal determinants of an outcome. Recognising these limitations is critical for avoiding overinterpretation of explanatory outputs and for guiding future work toward more rigorous causal interpretation frameworks.

In conclusion, SpaCEy represents a step toward explainable machine learning for spatial omics. By combining predictive accuracy with transparent explanation maps, it provides a framework that not only models clinical outcomes but also generates biologically interpretable insights into tissue structure. Its flexibility, scalability, and focus on explainability make SpaCEy a promising approach to advance spatial biology and precision oncology. As spatial omics technologies continue to evolve, SpaCEy can serve as a foundation for bridging high-dimensional molecular data with clinically actionable understanding of disease.

## Methods

### Hardware environment

All computations were performed on a workstation running Ubuntu 24.04.3 LTS with Linux kernel 6.8.0-79-generic. The system was equipped with an AMD Ryzen Threadripper PRO 5995WX processor (128 cores, 2.7 GHz base frequency) and NVIDIA RTX A6000 GPU.

### Data and preprocessing of the data

Transforming input data into a structured format is a key first step in developing graph-based algorithms for predictive modelling. For our datasets, we performed a transformation of the input data into spatial graphs using Delauney triangulation method, where each node represents a cell and the features correspond to protein abundance values^24^. These spatial graphs were then used as input for machine learning methods to address predictive problems.

We used three datasets in this study. The first dataset comprises samples from 416 patients with lung adenocarcinoma^25^. In this dataset, a 35-plex antibody panel was optimised to identify cancer cells, stromal cells, and innate and adaptive immune lineages with diverse functional substates. The second is the JacksonFischer dataset, comprising 720 imaging mass cytometry samples from 352 breast cancer patients across two cohorts: the University Hospital of Basel and the University Hospital of Zurich. This dataset also includes clinical features such as tumour size, tumour grade, clinical subtype, age, and overall survival^28^. The third dataset is the METABRIC dataset, which consists of 460 images from 405 unique patients^49^. Both datasets include clinical features of patients such as tumour size, tumour grade, clinical subtype, age, and overall survival. The basic statistics about these datasets are available in Supplementary Tables 1, 2 and 3, respectively.

The preprocessing pipeline transforms spatial proteomics data into graph neural network-ready format through a four-step process. First, data quality filtering removes images with fewer than 100 cells to ensure sufficient spatial density for meaningful analysis. Next, an adaptive edge length distribution calculation is performed for each sample. To reduce spurious long-range connections introduced by the graph construction process, edges longer than the 99th percentile of all edge lengths were removed. The graph construction phase then generates spatial graphs by applying Delaunay triangulation and retaining only edges shorter than the adaptive threshold, effectively filtering out long-distance connections that may not represent biologically relevant edges. As a data augmentation step, we implemented a patch-based approach to enhance computational efficiency and increase the number of training samples. Specifically, images with cell counts exceeding the median were divided into four spatial quadrants (upper-left, upper-right, lower-left, and lower-right) while maintaining their original spatial structure. Finally, the data preprocessing pipeline extracts and serialises node features (protein abundance values), edges, and clinical metadata (grade, tumour size, treatment, progression, survival data). Additional metadata, including cell-type and compartment annotations when available, were retained only for downstream post hoc interpretation and visualisation, and were not used for graph construction, model training, prediction, or explanation generation.

### Model architecture

We developed a model architecture, illustrated in Fig. 1, consisting of graph convolutional layers followed by fully connected (feed-forward) layers. The output layer consists of a single neuron that predicts a continuous value. After each layer, batch normalisation is applied preceding the ReLU activation function^56^. We used three different GNN layers to create predictive models, which are Graph Convolutional Neural Networks (GCN)^57^, Graph Attention Networks^58^, and Principal Neighbourhood Aggregation^59^. We created a generic framework that allows flexible training using a choice of GNN architecture. The hyperparameters used for model training, along with their corresponding value ranges, are provided in the Supplementary Table 4.

To project cell features and their relational structure into a new embedded space, the node features ${h}_{j}^{(l)}$ from layer *l* are first passed through a non-linear transformation function *ψ*, which in this case is the sigmoid function. The aggregation of neighbouring information is determined by the graph’s topology. This topology is represented by a normalised adjacency matrix *c*_*ij*_, which encodes the spectrally normalised edge weights between nodes *i* and *j*.

Following this transformation, an aggregation operation is applied across neighbouring nodes. Equation (1) summarises one of the message-passing mechanism employed in our models and provides the expression of a graph convolutional network (GCN) layer. The aggregated features from neighbouring nodes within the *l*-hop neighbourhood are then concatenated with the current layer representation of node *i*, and the result is modulated by a learnable global weight parameter. This process produces the representation of node *i* at the next layer, *l* + 1. The resulting layer can be formally expressed as shown in Eq. (1).

1 $${h}_{i}^{(l+1)}=\phi \left({h}_{i}^{(l)}\,{\bigoplus}_{j\in N(i)}\,{c}_{ij}\,\psi \left({h}_{j}^{(l)}\right)\right)$$

Here, ${h}_{i}^{(l+1)}$ represents the updated hidden representation of node *i* at layer *l* + 1, while ${h}_{i}^{(l)}$ denotes its representation at the previous layer *l*. The symbol *ϕ* refers to a learnable global weight parameter, and *ψ* is a non-linear activation function. The term ${h}_{j}^{(l)}$ corresponds to the hidden features of neighbouring nodes *j*, and *c*_*ij*_ indicates the spectrally normalised adjacency matrix element that modulates the influence of node *j* on node *i*. Finally, ⨁ denotes the aggregation operator (e.g., max, mean, or sum) used to combine neighbourhood information.

To obtain a fixed-length sample-level representation from graphs with variable numbers of cells, SpaCEy applies global mean pooling to the final-layer node embeddings. For a graph *G* = (*V*, *E*), the graph-level embedding is computed as

2 $${h}_{G}=\frac{1}{| V| } {\sum}_{i\in V}{h}_{i}^{(L)},$$

where ${h}_{i}^{(L)}$ denotes the final message-passing-layer embedding of node *i*. This pooled graph embedding is then passed to the fully connected layers for survival or classification prediction and is also used as the sample-level representation for UMAP visualisation and Leiden clustering. The model-training hyperparameter ranges are listed in Supplementary Table 6.

The loss function for the survival prediction used in this study is adapted from the DeepSurv model^60^, which applies a regularised negative log partial likelihood to handle survival data consisting of three components: a patient’s baseline features *x*, the event or censoring time *T*, and an event indicator *E* that specifies whether the event occurred. The objective of the model is to minimise (3):

3 $$\ell (\theta ):=-\frac{1}{N} {\sum}_{i:{E}_{i}=1}\left({\hat{h}}_{\theta }({x}_{i})-\log {\sum}_{j\in {{{\mathcal{R}}}}({T}_{i})}{e}^{{\hat{h}}_{\theta }({x}_{j})}\right)+\lambda \,\cdot \parallel \theta {\parallel }_{2}^{2}$$

where *N* denotes the number of individuals in the cohort, ${\hat{h}}_{\theta }({x}_{i})$ is the predicted log-risk for an individual *i*, ${{{\mathcal{R}}}}({T}_{i})$ denotes the risk set comprising all individuals still at risk at time *T*_*i*_, *E*_*i*_ ∈ {0, 1} indicates whether the event was observed, and *λ* is the coefficient of the *L*_2_ regularisation term.

For the classification tasks, we used binary cross-entropy as the loss function which measures the discrepancy between the predicted probability of the positive class and the true binary label. It is defined as:

4 $${{{{\mathcal{L}}}}}_{BCE}=-\frac{1}{N} {\sum}_{i=1}^{N}\left[{y}_{i}\log ({\hat{y}}_{i})+(1-{y}_{i})\log (1-{\hat{y}}_{i})\right],$$

where *y*_*i*_ ∈ {0, 1} is the true label for sample *i*, and ${\hat{y}}_{i}$ is the predicted probability of the positive class.

### Training, validation and test settings

To ensure robust model evaluation, we performed cross-validation while accounting for the presence of multiple samples from the same patients. To prevent data leakage and avoid overestimating model performance, samples originating from the same patient were grouped within the same fold. This ensured that the training and test sets remained independent with respect to patient identity. The same splits were used across all models to enable a fair comparison. We optimised hyperparameters separately for each method-aggregation combination using an internal cross-validation procedure within the training set. For classification tasks, we used the clinical outcome labels available for each dataset, such as binary progression status, whereas survival tasks used time-to-event outcomes together with censoring status. Other clinical metadata were retained for descriptive analyses and downstream interpretation but were not used as node features or direct model inputs.

### Survival analysis

For descriptive survival comparisons across clinical groups, we performed patient-level Kaplan–Meier analysis using the observed overall survival time and the event indicator provided in the clinical metadata. In datasets with repeated images or cores per patient, records were collapsed to a single patient-level entry before survival estimation to avoid duplicate patient contributions. Survival curves were estimated separately for each clinical group, and differences among groups were assessed using an omnibus log–rank test. This test compares, at each observed event time, the number of events observed in each group with the number expected under the null hypothesis that all groups share the same survival distribution; these deviations are then aggregated across event times to obtain a global test statistic and corresponding p-value. Censoring times were indicated directly on the Kaplan–Meier curves.

### Explainer model

Here, we adapted the GNNExplainer method, a perturbation-based approach designed to quantify the sensitivity of model predictions to input perturbations, thereby facilitating the interpretation of graph neural network decisions. This method learns two types of masks: a discrete feature mask *M*_*F*_ over node features and a continuous edge mask *M*_*E*_, both of which identify the most influential components in the graph through an optimisation process (Supplementary Fig. 2).

The masks are initialised randomly and optimised using a loss function based on mutual information, defined in Eq. (5):

5 $${{\max }_{{A}^{{\prime} }}}{{{\rm{MI}}}}\left(Y,({M}_{E},{M}_{F})\right)=H(Y)-H\left(Y| {A}^{{\prime} },{X}^{{\prime} }\right)$$

where MI denotes mutual information, *Y* represents the model predictions, *H*( ⋅ ) is the entropy function, ${A}^{{\prime} }$ denotes the masked adjacency matrix, and ${X}^{{\prime} }$ denotes the masked node features. The mutual information is computed using the *p*_norm_ distance between two row vectors of equal dimension, as shown in Eq. (6):

6 $$\parallel x{\parallel }_{p}={\left({\sum}_{i=1}^{n}| {x}_{i}{| }^{p}\right)}^{\frac{1}{p}}$$

The masked features and adjacency matrix are generated as follows:

7 $${X}^{{\prime} }=X\odot {M}_{F}^{{{{\rm{ext}}}}}$$

8 $${A}^{{\prime} }=A\odot \sigma ({M}_{E})$$

where  ⊙ denotes the element-wise product and *σ* represents the sigmoid function that maps the edge mask to [0, 1]^*n*×*n*^.

During the explanation step, the trained SpaCEy GNN parameters are kept fixed and only the mask parameters are optimised. Since *H*(*Y*) is fixed in Eq. (5), maximising the mutual information between the model output *Y* and the masked subgraph is equivalent to minimising the prediction-preservation loss under the masked graph, together with sparsity and entropy regularisation on the learned masks. Let *f*_*c*_(*X*, *A*) denote the *c*-th output of the frozen trained model on the original graph, and let ${\tilde{f}}_{c}(X\odot {M}_{F},A\odot \sigma ({M}_{E}))$ denote the corresponding output of the same frozen model when the node features and adjacency matrix are masked. Here, *M*_*F*_ denotes the feature mask after extension or broadcasting to match the node-feature matrix. Conceptually, the explainer objective is defined in Eq. (9):

9 $${{\min }_{M=[{M}_{F},\,{M}_{E}]}} {\sum}_{c=1}^{C}{\left\Vert {f}_{c}(X,A)-{\tilde{f}}_{c}\left(X\odot {M}_{F},A\odot \sigma ({M}_{E})\right)\right\Vert }_{p}+{\lambda }_{1}\parallel \sigma ({M}_{E}){\parallel }_{1}+{\lambda }_{2}H\left(\sigma ({M}_{E})\right)+{\lambda }_{3}H({M}_{F})$$

The first term penalises changes between the original trained-model prediction and the prediction obtained from the masked graph, with *C* denoting the number of output dimensions. The *ℓ*_1_ penalty on *σ*(*M*_*E*_) encourages sparse edge masks and prevents the explainer from retaining all graph edges, while the entropy penalties *H*(*σ*(*M*_*E*_)) and *H*(*M*_*F*_) encourage edge and feature mask values to become decisive, with values close to 0 or 1 rather than ambiguous intermediate values. As a result of this optimisation, the explainer model outputs the edge mask *M*_*E*_, which is subsequently used to compute node importance scores while the trained GNN weights remain unchanged.

### Determining the node importance values and important regions

After training the explainer model, node importance values were computed based on the learned edge weights. This process comprised the following steps:

*(i) Extracting k-hop subgraphs:* For each node *v*_*i*_, we defined its *k*-hop neighbourhood ${{{{\mathcal{N}}}}}_{k}({v}_{i})$, which includes all nodes and edges within *k* hops from *v*_*i*_.

*(ii) Aggregating edge importance values:* Given edge importance scores *w*_*jl*_ assigned by the explainer model, we computed the node importance ${{{\mathcal{I}}}}({v}_{i})$ by aggregating the edge weights of all edges within the corresponding subgraph ${{{{\mathcal{E}}}}}_{k}({v}_{i})$. Formally, the importance of node *v*_*i*_ is defined as in Eq. (10):

10 $${{{\mathcal{I}}}}({v}_{i})=\frac{1}{| {{{{\mathcal{E}}}}}_{k}({v}_{i})|} {\sum}_{({v}_{j},{v}_{l})\in {{{{\mathcal{E}}}}}_{k}({v}_{i})}{w}_{jl}$$

where ${{{{\mathcal{E}}}}}_{k}({v}_{i})$ denotes the set of edges in the *k*-hop subgraph centred on node *v*_*i*_, and *w*_*jl*_ represents the importance weight assigned to the edge connecting nodes *v*_*j*_ and *v*_*l*_. The resulting node importance values were subsequently used to identify biologically meaningful regions within the tissue that contributed most strongly to the model predictions.

### Evaluation metrics

We assessed model performance using a comprehensive set of classification and regression metrics. For the classification task, accuracy, area under the receiver operating characteristic curve (AUC), and F1-score were used to quantify discriminative performance. Accuracy measures the proportion of correctly classified samples and is defined as:

$${{\mathrm{Accuracy}}} \,=\frac{{{\mathrm{TP}}}+{{\mathrm{TN}}}}{{{\mathrm{TP}}}+{{\mathrm{TN}}}+{{\mathrm{FP}}}+{{\mathrm{FN}}}},$$

where TP, TN, FP, and FN denote true positives, true negatives, false positives, and false negatives, respectively. Area Under the ROC Curve (AUC) quantifies the model’s ability to discriminate between classes by integrating the true positive rate (TPR) as a function of the false positive rate (FPR):

$${{\rm{AUC}}}=\int_{0}^{1}{{\rm{TPR}}}({{\rm{FPR}}}) \, d(\,{{\rm{FPR}}}).$$

F1-score provides a harmonic mean of precision and recall and is given by:

$${{\rm{F1-score}}}=2\cdot \frac{{{\rm{Precision}}}\cdot {{\rm{Recall}}}}{{{\rm{Precision}}}+{{\rm{Recall}}}},$$

where

$${{\mathrm{Precision}}}\,=\frac{{{\mathrm{TP}}}}{{{\mathrm{TP}}}+{{\mathrm{FP}}}},\qquad \,{{\mathrm{Recall}}}\,=\frac{{{\mathrm{TP}}}}{{{\mathrm{TP}}}+{{\mathrm{FN}}}}.$$

Area under the Precision–Recall Curve (AUPRC) quantifies the trade-off between precision and recall across different classification thresholds. It is defined as:

$${{\rm{AUPRC}}}=\int_{0}^{1}{{\rm{Precision}}}({{\rm{Recall}}})\,d(\,{{\rm{Recall}}}).$$

To assess the performance of our survival predictive models, we employed concordance index (C-index). C-index is a metric used in survival analysis to evaluate the model’s ability to correctly rank survival times. It is defined as:

$${{\rm{C-index}}}\,=\frac{1}{| P|} {\sum}_{(i,j)\in P}{\mathbb{1}}({\hat{y}}_{i} < {\hat{y}}_{j})$$

where *P* is the set of all comparable pairs, ${\hat{y}}_{i}$ is the predicted risk score or survival time, and ${\mathbb{1}}(\cdot )$ is the indicator function. A C-index of 1.0 denotes perfect concordance, while 0.5 indicates random ordering.

### Synthetic dataset generation

To ensure controlled experimental conditions, we generated synthetic datasets designed to simulate the structure of real clinical tissue imaging data. Node features were represented as random vectors with dimensionality equal to the number of markers. These features were sampled from a standard normal distribution, $x \sim {{{\mathcal{N}}}}(0,1)$, and additional noise was incorporated, $\epsilon \sim {{{\mathcal{N}}}}(0,0.1)$, to introduce variability.

For each simulation, the samples follow a normal distribution with mean 1300 and standard deviation 400, clipped between 100 and 2500. Sparse connectivity was introduced by assigning edges with probability *p* = 0.1, while ensuring overall graph connectivity. In addition, each node was assigned two-dimensional spatial coordinates to capture positional information within the simulated tissue microenvironment.

Labels were generated to provide meaningful regression targets that combined both node-level and structural information. Node importance weights were sampled from a normal distribution, $w \sim {{{\mathcal{N}}}}(0,1)$. These were used to define a graph-level feature, *f*_graph_ = mean(*x*) ⋅ *w*, while a structural feature was derived from edge density, *f*_struct_ = ∣*E*∣/(∣*V*∣ × (∣*V*∣ − 1)). The final regression label was obtained as a combination of these components with additional noise, *y* = *f*_graph_ + *f*_struct_ + *ϵ*, where $\epsilon \sim {{{\mathcal{N}}}}(0,0.05)$.

As an explainer-validation experiment separate from the scalability simulation, we generated 300 synthetic tumour-stroma graphs with 220–320 cells per graph, multiplex-like node features. Each graph contained similar number of intra-tumoral CD8 cells and stromal CD8 cells, and the label depended on whether intra-tumoral CD8 cells formed a compact motif rather than a dispersed intra-tumoral pattern. This yielded a controlled benchmark in which the ground-truth nodes were known a priori, allowing direct evaluation of whether explainer model assigned higher importance to intra-tumoral CD8 nodes than to stromal CD8 nodes.

### Scalability analysis

To assess computational performance, we conducted a systematic scalability analysis using synthetic spatial proteomics-like datasets. The objective was to quantify how training time, GPU memory consumption, and utilisation rates scale with increasing dataset dimensionality and sample size.

A factorial experimental design was implemented, varying the number of molecular markers across five levels (30, 100, 300, 500, and 1000) and the number of samples across six levels (50, 100, 200, 500, 1000, and 1500), resulting in 30 distinct configurations. For each configuration, we recorded different statistics such as average epoch training time, average GPU utilisation percentage during training and memory usage. Each experiment was repeated three times to ensure consistency, and all runs were performed under identical hardware conditions. This benchmarking workflow enabled quantitative assessment of the computational scalability of our model.

### Statistics and reproducibility

No statistical method was used to predetermine sample size. Sample sizes were determined by the availability of samples in the published datasets used in this study. Images containing fewer than 100 cells were excluded during preprocessing to ensure sufficient spatial density for graph construction; no other data were excluded from the analyses. The Investigators were not blinded to allocation during experiments and outcome assessment. Model performance was evaluated using patient-level cross-validation, with samples from the same patient assigned to the same fold to prevent data leakage. The same data splits were used across compared models. Statistical tests, sample sizes, and cross-validation procedures are specified in the corresponding Methods sections and figure legends.

### Reporting summary

Further information on research design is available in the Nature Portfolio Reporting Summary linked to this article.

## Supplementary information

Supplementary Information

Description of Additional Supplementary Files

Supplementary Data 1-4

Reporting Summary

Transparent Peer Review file

## Source data

Source Data

## Data Availability

The datasets used in this study were obtained from previously published studies and are publicly available from their original publications. The lung tumour immune microenvironment dataset used in this study was obtained from Sorin et al.^25^ and is available at https://doi.org/10.1038/s41586-022-05672-3. The breast cancer single-cell pathology (JacksonFischer) dataset was obtained from Jackson et al.^28^ and is available at https://doi.org/10.1038/s41586-019-1876-x. The METABRIC breast cancer imaging dataset was obtained from Ali et al.^49^ and is available at https://doi.org/10.1038/s43018-020-0026-6. The colorectal cancer dataset was obtained from its original publication and is available at https://doi.org/10.1016/j.cell.2020.10.021. The preprocessed datasets used in this study have been deposited in Zenodo and are publicly available at https://doi.org/10.5281/zenodo.17710588. Any additional requests for information can be directed to, and will be fulfilled by, the corresponding authors. Source data are provided with this paper.

## Code availability

SpaCEy was implemented within the NVIDIA PyTorch Geometric (PyG) container (nvcr.io/nvidia/pyg:24.01-py3). The framework builds on PyTorch for deep learning and PyTorch Geometric for graph-based deep learning modelling. For downstream analysis and preprocessing, we used Scanpy (v1.9.8). In addition, Leidenalg (v0.10.2) was employed to perform Leiden clustering for community detection. All code used for data preprocessing, model training, and downstream analyses is available at https://github.com/saezlab/SpaCEy. The version of the code used in this study has been archived on Zenodo at https://doi.org/10.5281/zenodo.22073069.
