# Supplementary material for: SpaCEy links spatial tissue patterns to clinical outcomes using explainable graph neural networks
Source: Nat Commun. 2026 Sep 26;17:10232. doi: 10.1038/s41467-026-77924-z (PMC13615965; doi:10.1038/s41467-026-77924-z)
Supplement: Supplementary file 1 [file 41467_2026_77924_MOESM1_ESM.pdf]

Supplementary Material

Supplementary Table 1. *LUAD Dataset Statistics*

Description	Value
Standard deviation of cells per patient	1136.03
Average cells per sample	3996
Median cells per sample	4057
Minimum cells per sample	610
Maximum cells per image	8869
Total patients in clinical data	416
Patients with survival data	416
Patients with death information	416
Patients with progression information	404
Patients with stage information	415
Patients with histological pattern information	416

Supplementary Table 2. *JacksonFischer Dataset Statistics*

Description	Value
Total number of patients	357
Total number of images	735
Average cells per image	1730.29
Standard deviation of cells per image	1296.07
Maximum cells per image	6908
Minimum cells per sample	63

Maximum cells per sample	13291
Minimum images per patient	1
Maximum images per patient	7

Supplementary Table 3. *METABRIC Dataset Statistics*

Metric	Value
Total number of images	460
Total number of patients	405
Mean Images per Patient	1.135802469
Max Images per Patient	3
Std Images per Patient	0.383866675
Total number of patients	405
Total number of images	460
Average cells per image	884.1
Standard deviation of cells per image	503.6097397
Minimum cells per image	7
Maximum cells per image	3085
Minimum cells per sample	7
Maximum cells per sample	3438
Minimum images per patient	1
Maximum images per patient	3
Median Images per Patient	1
Std Images per Patient	0.383866675

Supplementary Table 4. *Hyperparameter values for model training This table lists the key hyperparameters and their respective search ranges explored during model optimization. Each model was tuned using grid or randomized search strategies within these parameter spaces to identify optimal configurations for predictive performance and generalization. The hyperparameter ranges include structural, regularization, and training parameters relevant to both classical machine learning and survival analysis models.*

**Category**	**Hyperparameter**	**Values**	**Description**
**Model Architecture**	Number of GCN Layers	2	Number of graph convolutional layers
**Model Architecture**	Number of Feed-Forward Layers	1, 2	Number of fully connected layers
**Model Architecture**	GCN Hidden Dimension	16, 32, 64, 128	Hidden dimension size for graph convolutional layers
**Model Architecture**	Fully Connected Layer Dimension	64, 128, 256	Hidden dimension size for fully connected layers
**Model Architecture**	Dropout Rate	0.2, 0.3	Dropout probability for regularization
**Training Parameters**	Learning Rate	0.1, 0.01, 0.001, 0.0001	Initial learning rate for optimization
**Training Parameters**	Batch Size	16, 32, 64	Number of samples per training batch
**Training Parameters**	Epochs	200	Maximum number of training epochs
**Training Parameters**	Weight Decay	0.1, 0.001, 0.0001, 1e-5	L2 regularization coefficient
**Learning Rate Scheduler**	Factor	0.5, 0.8, 0.2	Factor by which learning rate is reduced
**Learning Rate Scheduler**	Patience	5, 10	Number of epochs with no improvement before reducing LR
**Learning Rate Scheduler**	Minimum Learning Rate	0.00002, 0.0001	Lower bound for learning rate
**Model-Specific**	GAT Heads	1, 3, 5	Number of attention heads (GAT model only)
**Model-Specific**	Aggregators	min, max, sum, mean, sum max	Aggregation functions
**Model-Specific**	PNA Scalers	identity, amplification	Scaling functions (PNA model only)

Supplementary Table 5. *Crop-sensitivity summary for the JacksonFischer dataset. Risk-tertile agreement denotes the fraction of crops assigned to the same low/mid/high risk tertile as their parent ROI. Embedding-cluster agreement denotes the fraction of crops assigned to the same full-ROI Leiden embedding cluster as their parent ROI.*

Crop Type	Number of valid samples	Spearman correlation	Risk-tertile agreement	Mean cosine similarity (embeddings)	Cluster Agreement	Mean cell-type composition correlation
Center	319	0.945	0.818	0.974	0.812	0.830
Upper-left	316	0.944	0.826	0.928	0.848	0.870
Upper-right	311	0.935	0.817	0.918	0.83	0.880
Lower-left	315	0.898	0.798	0.875	0.765	0.812
Lower-right	317	0.943	0.817	0,903	0.833	0.922

Supplementary Table 6. *Context-stratified marker shifts in JacksonFischer SpaCEy-important nodes. Cells were first grouped by broad cellular context, and marker abundance was summarized as the ROI-level mean among important nodes within each context. Positive lower-minus-higher survival differences indicate higher abundance in lower-survival ROIs. The strongest lower-survival-associated context was defined as the context with the largest positive standardized mean difference (SMD) for each marker. A context-specific marker shift should be interpreted as marker abundance within important nodes assigned to that broad context.*

**Marker**	**Strongest context**	**SMD**
**Ki-67**	**Tumor**	**0.325**
**CD3**	**Stroma**	**0.421**
**Slug**	**Stroma**	**0.462**
**c-Myc**	**Stroma**	**0.507**

Supplementary Table 7. *Summary of targeted subtype analysis for reported lower-survival markers. Marker abundance was summarized as patient-level mean abundance among hard-mask SpaCEy-important nodes. Lower- and higher-survival groups were defined using group-specific median overall survival.* SMD = standardized mean difference for lower- versus higher-survival patient groups, averaged across the four reported markers.

Analysis group	Markers higher in lower-survival group	FDR-supported markers	Mean SMD
Full cohort	4/4: Slug; c-Myc; CD3; Fibronectin	Slug; c-Myc; CD3; Fibronectin	0.39
HR+HER2-	4/4: Slug; c-Myc; CD3; Fibronectin	Fibronectin	0.29
Combined non-HR+HER2-	4/4: Slug; c-Myc; CD3; Fibronectin	Slug; c-Myc; CD3	0.47
TripleNeg	4/4: Slug; c-Myc; CD3; Fibronectin	None	0.39
HR+HER2+	3/4: Slug; CD3; Fibronectin	None	0.13
HR-HER2+	3/4: Slug; c-Myc; Fibronectin	None	0.24

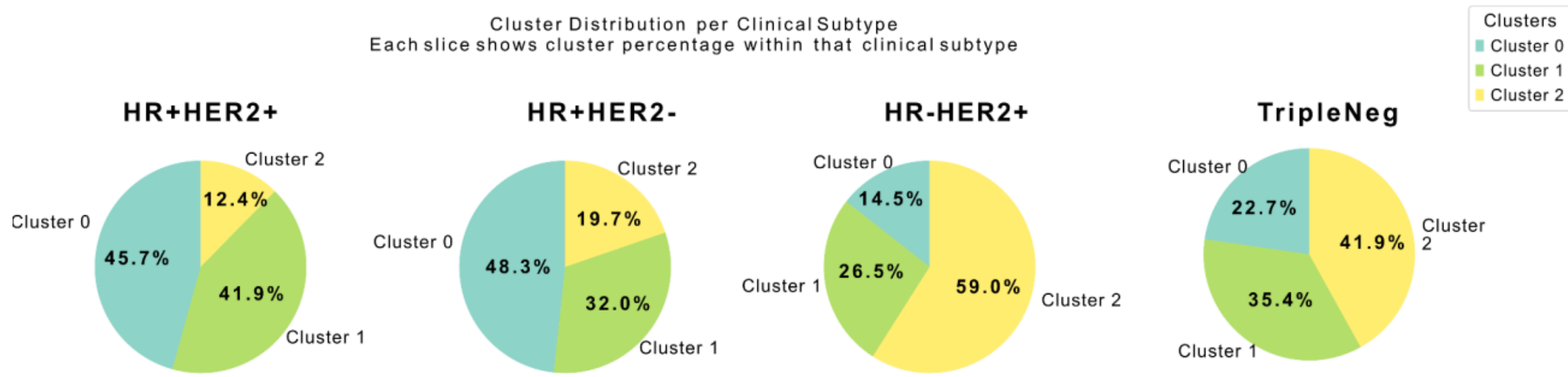

Supplementary Figure 1. Distribution of clinical subtypes across clusters in the JacksonFischer dataset.

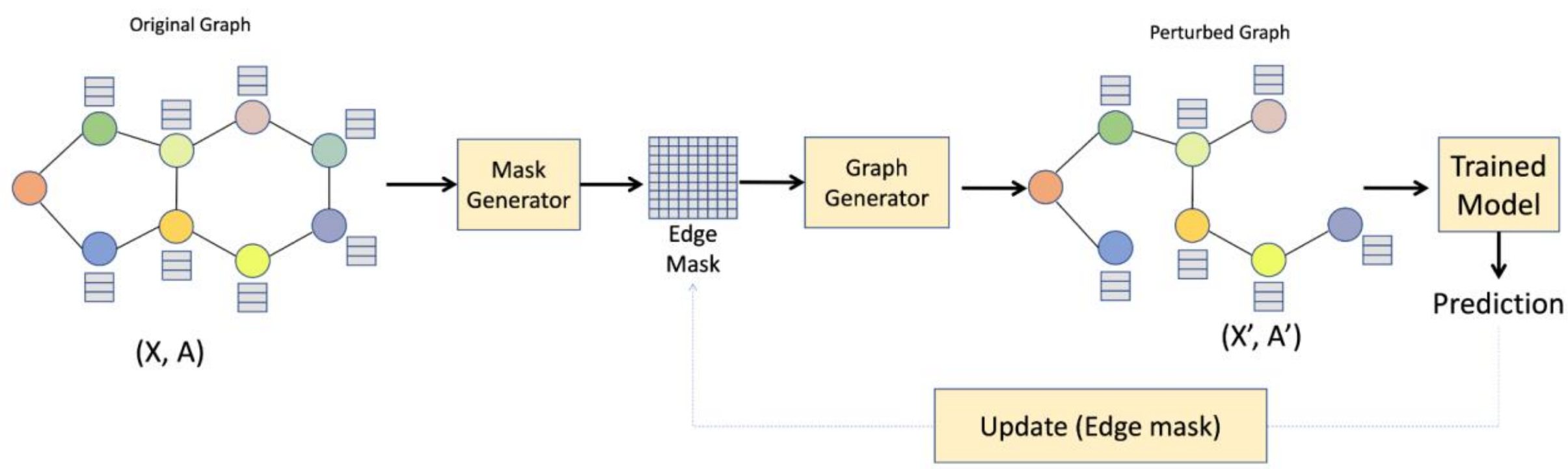

Supplementary Figure 2. GNNExplainer model training The general framework of GNNExplainer considers a graph G=(X,A), where the input graph is first passed through a Mask Generator. The masked representations are then processed by a Graph Generator to project them back into the original graph space, yielding(X′,A′). These modified inputs are fed into the trained GNN model and the resulting prediction f(X′,A′) is used in a mutual information–based loss function to optimize the Mask Generator. The optimization process ultimately produces refined masks that highlight the most relevant nodes, and edges for the model's prediction.

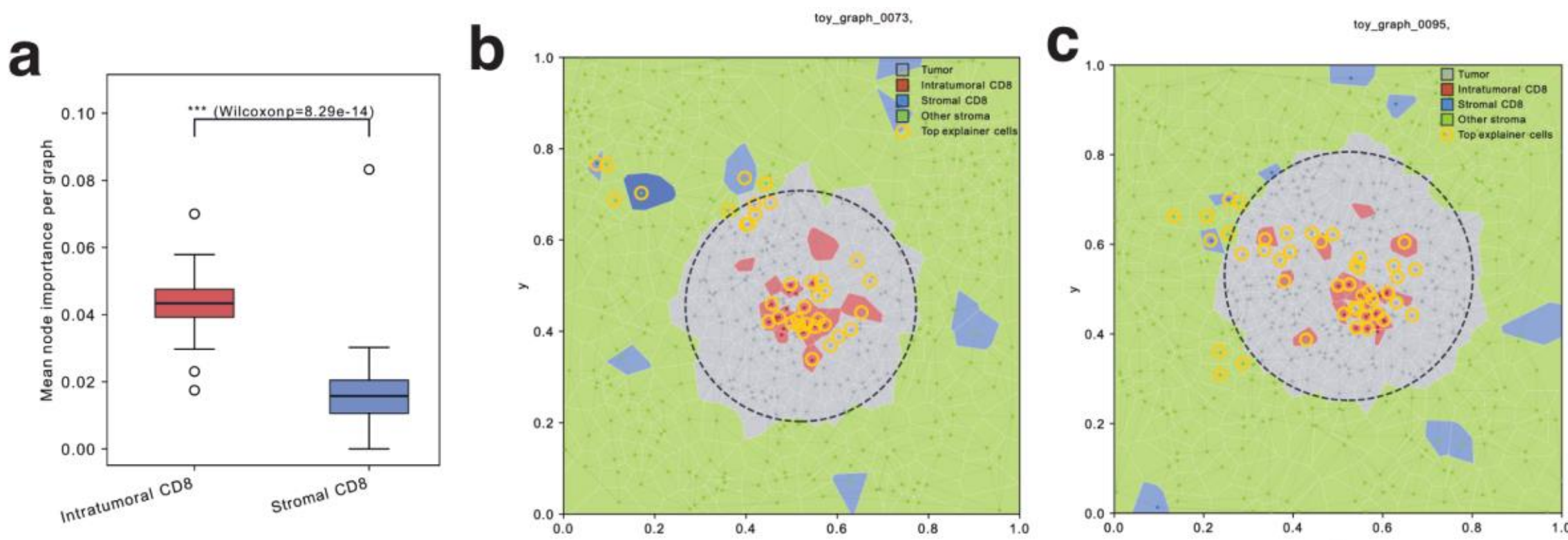

Supplementary Figure 3. SpaCEy explainer importance visualisation on synthetic dataset. **a)** Node-importance scores assigned by the explainer were compared across simulated node groups in held-out positive synthetic tumour-stroma graphs. Intratumoral CD8 nodes received higher importance scores (top explainer nodes) than stromal CD8 nodes, supporting that the explainer preferentially recovers the known ground-truth intratumoral CD8 signal rather than highlighting CD8 cells outside the tumour compartment. Simulated cell-type and compartment labels were used only for ground-truth evaluation and post hoc interpretation, not as model inputs. **b)** A representative synthetic sample demonstrating the selected important regions along with the annotations. **c)** Second representative synthetic sample demonstrating the selected important regions along with the annotations

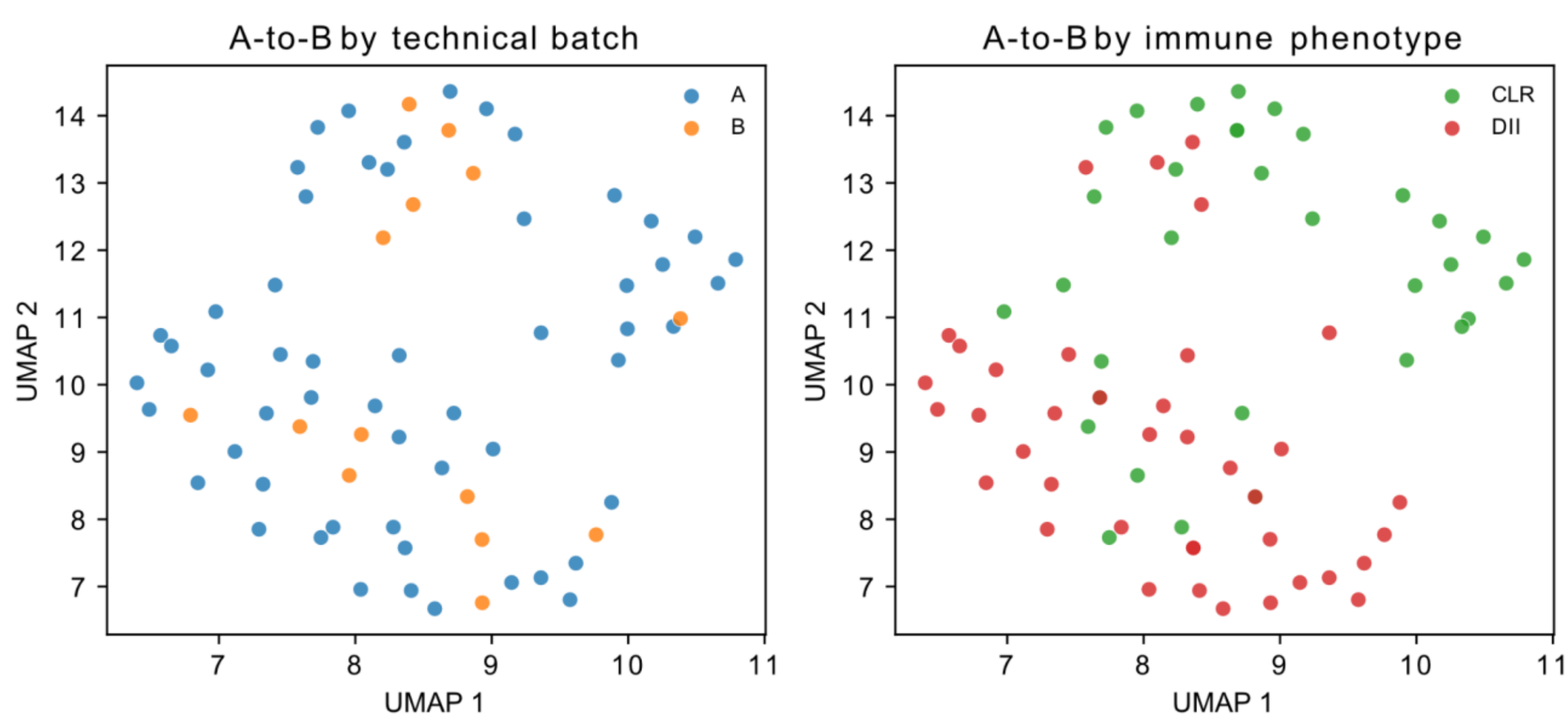

Supplementary Figure 4. CODEX cross-batch sample embedding visualisation for the A-to-B transfer model. UMAP projection of sample-level embeddings extracted from the trained of SpaCEy model in the A-to-B cross-batch setting. The same embeddings are shown coloured by technical batch identity (left) and by immune phenotype, CLR versus DII (right). Samples do not

separate strongly by technical batch, supporting that the learned representation is not dominated by batch identity in this transfer direction.

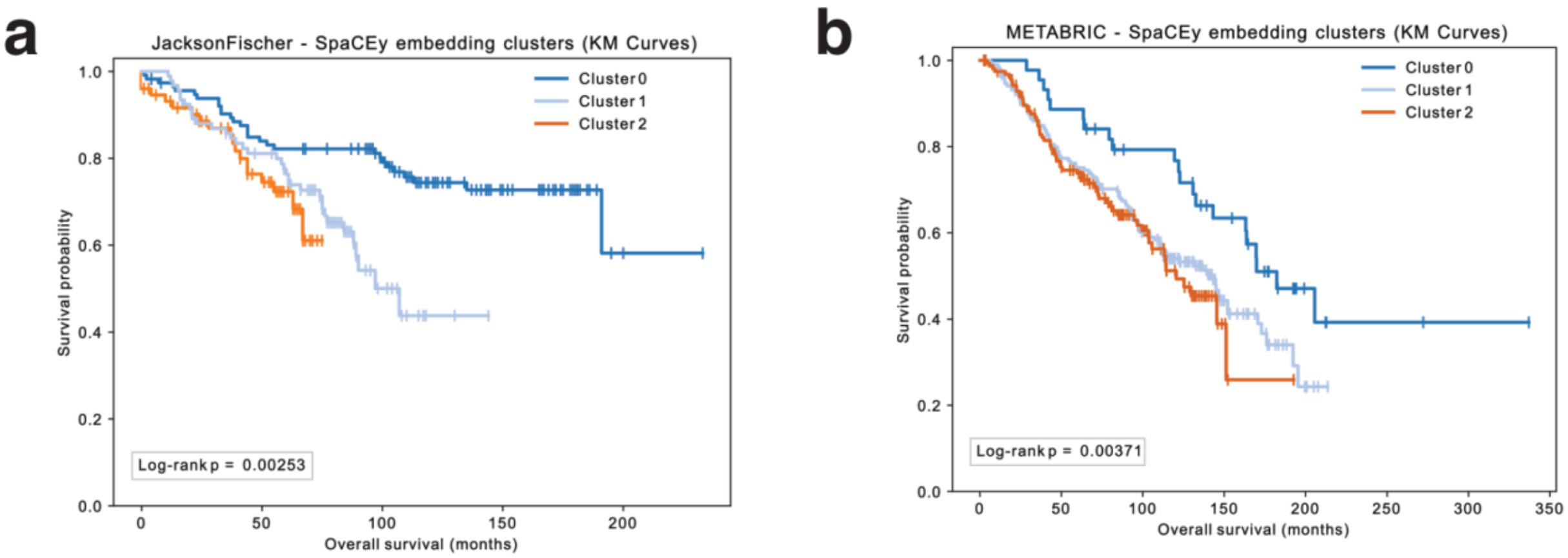

Supplementary Figure 5. SpaCEy embedding-cluster Kaplan-Meier analysis. Kaplan-Meier curves showing patient-level survival across outcome-aligned **a)** SpaCEy embedding clusters for the JacksonFischer dataset. Global log-rank tests across SpaCEy clusters showed significant survival separation in JacksonFischer (p = 0.00253) **b)** SpaCEy embedding clusters for the METABRIC dataset (p = 0.00371).

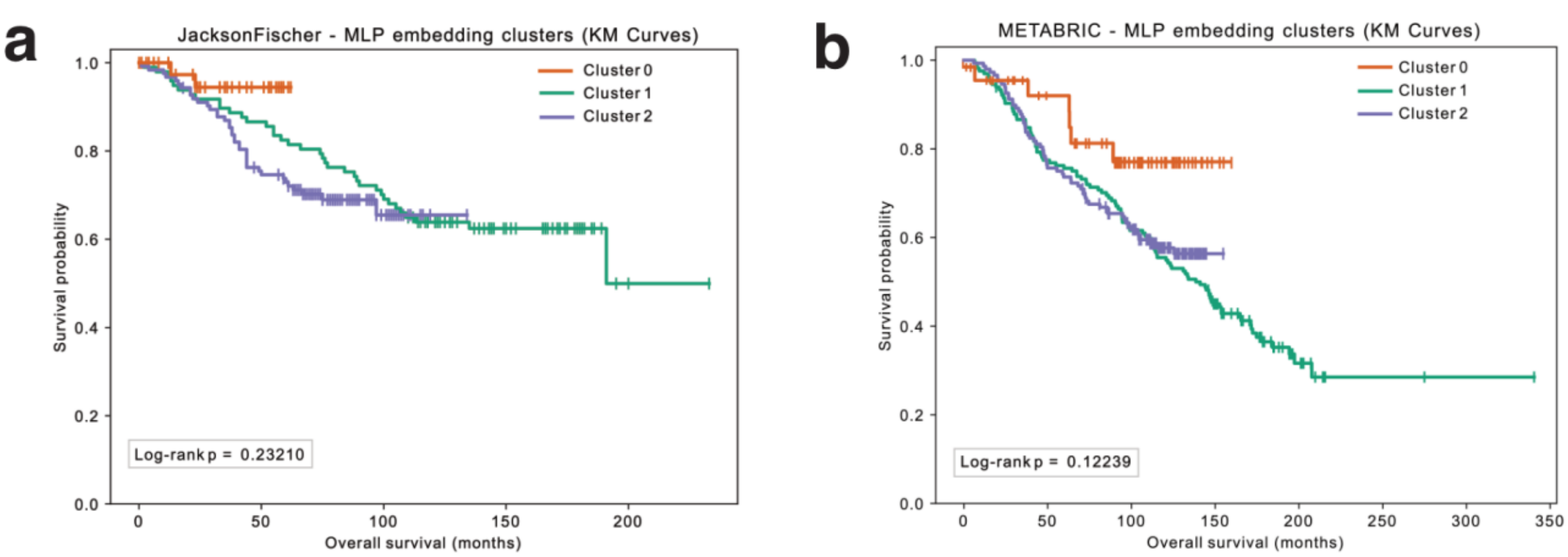

Supplementary Figure 6. Non-spatial MLP embedding-cluster Kaplan–Meier analysis. Kaplan–Meier curves showing patient-level survival across embedding clusters derived from a non-spatial supervised multilayer perceptron (MLP) baseline. **a)** Kaplan–Meier survival curves for the three embedding-derived clusters in the JacksonFischer breast cancer cohort. **b)** Kaplan–Meier survival curves for the three embedding-derived clusters in the METABRIC breast cancer cohort. Sample-level MLP embeddings were clustered into three groups and evaluated using patient-level Kaplan–Meier analysis with global log-rank tests. The non-spatial MLP baseline did not show significant global survival separation in JacksonFischer (global log-rank p = 0.23210) or METABRIC (global log-rank p = 0.12239).

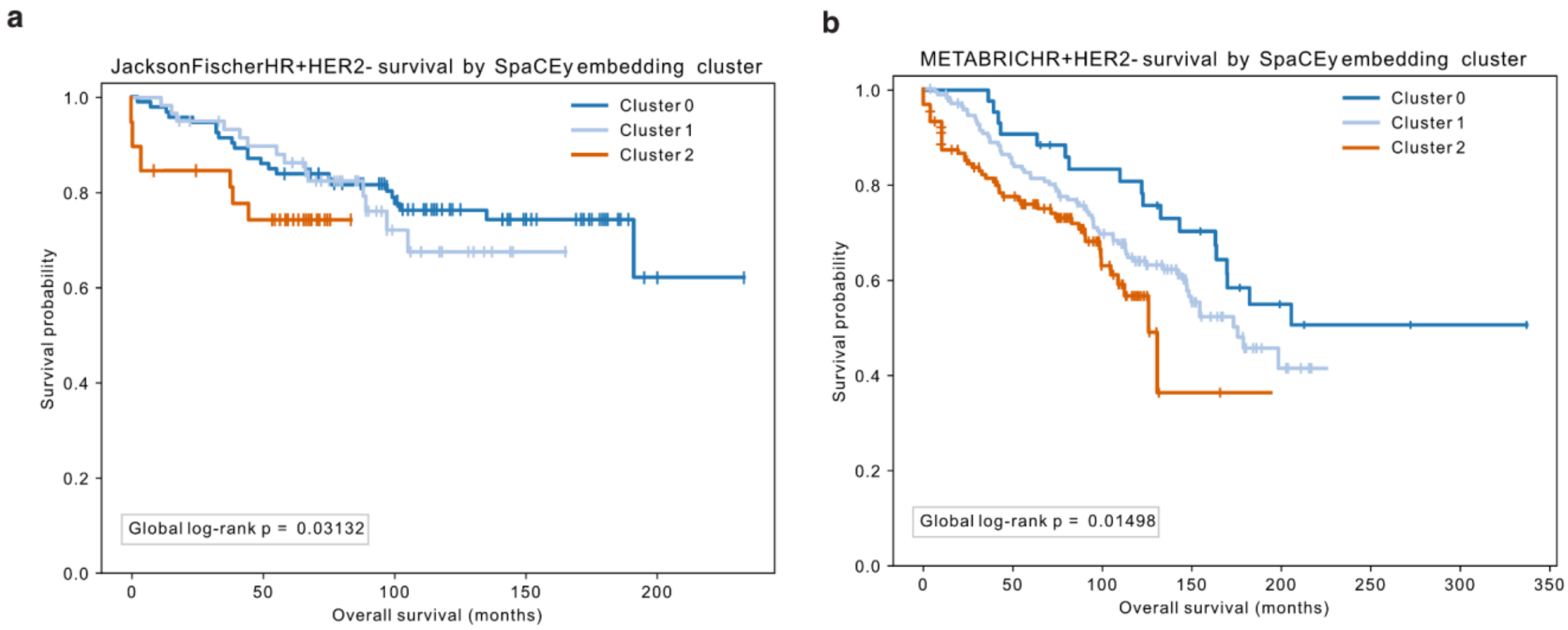

Supplementary Figure 7. SpaCEy embedding-cluster Kaplan–Meier analysis in HR+HER2- breast cancer. Kaplan–Meier curves showing patient-level overall survival across SpaCEy embedding clusters after restricting the breast cancer cohorts to HR+HER2- patients. **a)** Kaplan–Meier survival curves for the three SpaCEy embedding-derived clusters in the HR+HER2- subset of the JacksonFischer cohort. **b)** Kaplan–Meier survival curves for the three SpaCEy embedding-derived clusters in the HR+HER2- subset of the METABRIC cohort. Sample-level SpaCEy embeddings were clustered after model training, and survival differences across clusters were assessed using global log-rank tests.
