# Supplementary material for: SpaCEy links spatial tissue patterns to clinical outcomes using explainable graph neural networks
Source: Nat Commun. 2026 Sep 26;17:10232. doi: 10.1038/s41467-026-77924-z (PMC13615965; doi:10.1038/s41467-026-77924-z)
Supplement: Supplementary file 2 [file 41467_2026_77924_MOESM2_ESM.pdf]

Descriptions of Additional Supplementary Files

Supplementary Data 1. Baseline Hyperparameters

Hyperparameter search spaces used for the baseline models evaluated in the study. The file lists the models, corresponding hyperparameters, and the values or ranges considered during model optimization.

Supplementary Data 2. Scalability Analysis

Results of the computational scalability analysis across configurations varying in the number of molecular markers and samples. The file reports the number of markers and samples, average nodes per sample, average epoch time, total training time, peak GPU memory usage, and average GPU memory utilization.

Supplementary Data 3. HR+HER2- versus other-subtype marker abundance comparison among lower-survival JacksonFischer patients

Patient-level mean marker abundance was computed within important nodes. Lower-survival patients were defined using the full-cohort median overall survival split. Marker abundance was compared between HR+HER2- patients and patients from all remaining receptor-defined subtypes using two-sided Mann-Whitney U tests, with Benjamini-Hochberg FDR correction across markers. The table reports mean and median marker abundance, effect direction, raw p-values, and FDR-adjusted p-values.

Supplementary Data 4. Channels used in the JacksonFischer and METABRIC breast cancer datasets

The table lists all channels included in the spatial proteomics analyses and indicates whether each channel was present in the JacksonFischer dataset, the METABRIC dataset, or both. Channels are classified as shared, JacksonFischer-only, or METABRIC-only according to their availability across datasets.
