# Supplementary material for: SpaCEy links spatial tissue patterns to clinical outcomes using explainable graph neural networks
Source: Nat Commun. 2026 Sep 26;17:10232. doi: 10.1038/s41467-026-77924-z (PMC13615965; doi:10.1038/s41467-026-77924-z)
Supplement: Supplementary file 4 [file 41467_2026_77924_MOESM5_ESM.pdf]

nature portfolio

Peer Review File

SpaCEy links spatial tissue patterns to clinical outcomes using explainable graph neural networks

Corresponding Author: Dr Jovan Tanevski

This file contains all reviewer reports in order by version, followed by all author rebuttals in order by version.

Version 0:

Reviewer comments:

Reviewer #1

(Remarks to the Author)
In this manuscript, Rifaioglu et al. (the authors) have proposed a new graph neural network-based tool: SpaCEy to encode spatially-resoled single-cell omics data and use the model to predict clinical outcomes including tumor progression and overall prognosis. The authors have further demonstrated how this graph neural network can be coupled with a interpretability tool (GNNExplainer) to highlight "important" contributing regions to the predictions and showed how analysis of these important regions can reveal biologically meaningful insights. The overall motivation and design of the approach is well-presented, my main concern is that the biological interpretation results, which are highlighted as the main contributions in the introduction section, are a bit underwhelming as these insights are not really spatially-resolved and haven't fully utilized the potential of the input datasets. There are a few other points that can be improved or presented better. Please see below for a list of major and minor concerns:

1. There are many places where authors presented box plots of survival length (Fig2a, 3a, 3g, 4a, 4e), which I believe is not the standard approach. Due to the presence of right-censored data, these box plots could be misleading, please use KM curves instead.

2. From what I understand, the UMAP and clustering were performed using embeddings of the whole regions (ROIs). This is different from classic approach (e.g., Cellular Neighborhood) or SPACE-GM (i.e., k-hop subgraphs), as SpaCEy embeddings have aggregated information across all cells from the ROIs. However, selection of the ROIs happened before computational analysis and introduced human sampling bias. In other words, neighborhoods or k-hop subgraph approaches ensure full knowledge for each data point (since they focus on smaller sub-regions within the ROI), while SpaCEy embedding is subject to partial knowledge: areas outside the ROIs could contain more important information but are now excluded due to experimental sampling. My concern is how such sampling bias, or edge effect, could influence the embeddings and their interpretations. For example, if during inference we only use the center crops (or any crop, such as top left corner) of the input regions, how would results change?

3. Further to point #2, authors mentioned in the methods that "images with cell counts exceeding the median were divided into four spatial quadrants", which seems to me a very ad hoc solution. I recommend a more systematic way to define field-of-view for each data point in the embedding. Furthermore, it is increasingly common to have whole-slide tissue imaging data from spatial omics (CODEX, Xenium, MERFISH, etc.), how would SpaCEy handle that type of data as it could be 1000 times larger than the ones processed in this work?

4. This paper highlights SpaCEy's capabilities in identifying and summarizing key biological insights from modeling spatially-resolved data. However, most of the interpretation results are like: "In Cluster 0 (higher survival), we observed higher localized expression of PR while Cluster 2 (lower survival) showed an enrichment of fibronectin, β-catenin and CK19." These results are far from spatially-resolved (more examples in point #6 below). For example, fibronectin expression in CAF versus Cancer cells versus endothelial cells (intratumoral or extratumoral) could all have different indications. These current biological results are too broadly-defined and can hardly be translated into a verifiable hypothesis to allow further investigation.

5. Interpretation results of SpaCEy were based on "important" nodes, which by itself is a very vague and variable concept (according to Fig3e). What if tumoral areas are important in one region and stromal areas are important in another region?

Then during comparison stromal biomarkers (Vimentin, aSMA, fibronectin) will surely appear (and they did appear in Fig4c), but can you conclude that more stromal areas mean better/worse prognosis? It is unclear to me how much of that is due to sampling bias (point #2 above) and how much is actually biologically meaningful? In other words, it is hard to do apple-to-apple comparison in this setting, because we don't have a consistent understanding about what are the "important" regions. Comparing tumor against stroma from different patients, for example, is not typically an experiment that will yield valuable results.

6. Overall, the biological interpretations in this manuscript are very broad/vague and need further clarification or investigation:
- Fig2c shows more FoxP3, but are there more Tregs? Are are the Tregs located in tumor, or in stroma? Fig2d suggested more Tc and Th in "No progression" group, are they found in the tumor?
- In page 5, what are CD4+/CD8+ T cells? I don't think these are commonly recognized.
- Fig3c states that high CD3 is relevant to worse prognosis, which is counter-intuitive. Where are all the T cells located? Are they in the tumor or stroma? What can we know about these idle T cells?
- Ki67 appears in Fig3c, which is a hallmark proliferation marker. It will appear in both tumor cells and immune cells. Which ones are important nodes here?
- Authors stated: "Ki-67, a widely used proliferation marker, indicates aggressive tumour growth, and elevated Ki-67 levels are associated with poor survival in breast cancer". I believe certain therapies are also more effective for patients with stronger Ki-67, to say that Ki-67 level is unconditionally associated with poor survival in breast cancer could be factually incorrect.
- Fig4d suggests fibronectin, vimentin, bCatenin, SMA as differentially-expressed biomarkers, and myofibroblast and fibroblast as key cell type differences at the same time. Once again, is it because cluster 2 has more stromal areas labelled as important? If yes, do you think that the ratio of stromal area is a strong indicator of prognosis?
- Authors' interpretations are: "A high abundance of vimentin, a well-known marker of EMT, is associated with enhanced migratory and invasive behaviour in breast cancer cells, promoting metastasis." How would you know that the signal of Vimentin here is coming from cancer cells but not stromal cells (which commonly express Vimentin)?

7. The generalization experiment between JacksonFischer and METABRIC can be done by training on one dataset and testing on the other, to show both quantitative prediction performances and interpretation results.

8. This is not discussed in the manuscript, but most spatial omics experiments (proteomics and transcriptomics) suffer from batch effects. One key reason behind using cell types is because discretized version of the data are more stable and less subject to batch-specific signals. How will SpaCEy deal with batch effect when studying multiple batches of examples?

9. Since authors have already performed simulation studies, I think it makes sense simulating a toy dataset to first justify that GNNExplainer can correctly pick the important features that contribute to the task. For example, by predicting prognosis using a simulated TME dataset where number of tumor-infiltrating CD8 T cells (not including stromal CD8 T cells) is the only correlate of the task label, we can evaluate whether the intra-tumor CD8 T cells are being assigned higher importance scores than extra-tumoral CD8 T cells.

10. In Fig5, where authors compared SpaCEy against baseline methods, I think it is necessary to include a basic Cox regression model for the survival modeling task.

Minor:

11. Introduction: “highlights spatially contiguous nodes and edges” needs clarification, graphs are by design discrete.

12. Further to point #1, section 2.3 stated that "The objective was to predict overall survival (hereafter referred to as survival) values using this dataset", where Fig2ab only show survival lengths. This is later clarified in Methods section that the model is doing a proper survival modeling, but I would prefer this information to be revealed in the main text to avoid confusion.

13. Fig2a suggested that patients with progression have overall longer OS than patients with no progression, which is counter-intuitive. I believe it makes sense to show the full KM curves and justify how the labels were determined. In addition, are the model outputs (logits, probabilities, etc.) correlated with patient risk?

14. If I understand correctly, GNNExplainer only assigns unsigned importance scores. How would you acquire the directionality for these important nodes? Like can we know this important node has a positive/negative impact on prognosis predictions?

15. Fig6 discusses the scalability of SpaCEy, but there is no simulation for whole-slide samples with 1M+ cells.

16. RMSE is mentioned in the Methods section, where is it used? Which task is using RMSE for metrics?

(Remarks on code availability)

Reviewer #2

(Remarks to the Author)
In this manuscript, the authors propose an explainable graph neural network (GNN)-based method (SpCEy) that extracts characteristic tissue patterns of cell organization that are correlated with clinical outcomes, given a set of spatial omics (proteome) data with outcome information. Its performance was tested using several public datasets. The possibility of discovering important spatial patterns is interesting, and this kind of approach is increasingly important. However, this work suffers from the following points:
1. Although the authors write, "A key novelty of SpaCEy lies in its ability to generate interpretable spatially contiguous regions," only one such example is shown in the figure (Fig. 3f). Moreover, the generality and medical plausibility of this finding are not well investigated; how many of the positive/negative samples in the cohort contain the motif? Was it also observed in the other cohort? In this regard, it is recommended to perform a small "silico perturbation" experiment: if the model "removes" a specific identified spatial motif from the graph, does the predicted survival score change significantly?
2. Although the authors mention "The input to SpaCEy are samples ..., represented by measurements of spatially resolved omics and paired clinical observations," it is not clear whether/how the clinical observations, such as the breast cancer subtype information, were used in the learning step. On the contrary, it looks evident that clinical outcome information, such as survival values, was used in the training. Thus, it does not make sense to me that the authors compare the survival value of their unsupervised clustering results with that of the original subtypes (Figs. 3a and 3b).
3. In the discussion, they note "The current study focuses on protein abundance data" as a limitation. However, from the title, abstract, and introduction, it is unclear what type of spatially resolved data was used. This can be very misleading for readers. Moreover, in the introduction, the authors do not cite any related work on spatial transcriptome analyses using GNNs. Considering past methods in spatial transcriptomics, the novelty of this work could be less significant.
4. Because the spatial graph depends entirely on the initial cell segmentation, the authors must comprehensively test its effects on model performance. A suggested analysis is a sensitivity analysis showing how the model performs when nodes are intentionally "jittered" (slightly moved) or when a percentage of cells are randomly "dropped" to simulate segmentation errors or missed detections. This would demonstrate that the framework is robust enough for real-world clinical samples with varying imaging quality.
5. The authors should provide more comprehensive information and a comparison of the datasets. For instance, how similar or different are the two breast cancer cohorts in terms of their features? Could the authors perform a joint analysis, including downstream analyses, to compare patients across different cohorts simultaneously? Would SpaCEy minimize technical differences between cohorts, such as batch effects?
Minor:
6. The authors mention colocalization of cancer and endothelial cells; however, Figure 2d shows only proportions, with no actual objective measure of colocalization. If they want to claim colocalization, they should use an approach other than proportion and provide clear examples from image data.
7. The authors should add significance when presenting a comparison relevant to their results, e.g., the boxplot in Figure 3b.
8. When describing Figure 3c, the authors refer to it as a spatially contiguous expression; however, Figure 3c is a simple dot plot. Please include relevant Figures to support your assertions.
9. In the analysis of HR+HER2- patients (Figure 3g), the main markers largely overlap with those from the whole cohort. This could indicate a bias towards this cohort in the general analysis. It would be interesting to perform a similar analysis for the other patient classes and see whether the overlap persists across all subclasses. If so, what kind of conclusions can be derived from this? Does the model capture only global differences, rather than specific ones?
10. The sentence "Consistent with prior findings in JacksonFischer dataset, elevated fibronectin expression has been linked to poor prognosis" in Subsection 2.5 may be misleading, as in previous analyses, fibronectin was only found to be relevant when investigating specific patients in the cohort. Can this be more related to a certain enrichment of these patients in the METABRIC cohort?

(Remarks on code availability)

Reviewer #3

(Remarks to the Author)
I co-reviewed this manuscript with one of the reviewers who provided the listed reports. This is part of the Nature Communications initiative to facilitate training in peer review and to provide appropriate recognition for Early Career Researchers who co-review manuscripts.

(Remarks on code availability)

Version 1:

Reviewer comments:

Reviewer #1

(Remarks to the Author)
I appreciate all the changes and additional experiments/clarifications/discussion made by the authors. These revisions have substantially strengthened the manuscript. I have a few remaining questions/confusions related to the revised texts and

additional results, please find them below:

Follow-ups:
R1.1: Thank authors for updating the figure panels. One follow-up suggestion is for the cluster analysis in Figure 3b/3g/4b/4e: it would also be beneficial to include KM curves grouped by SpaCEy-inferred clusters.

R1.4: Thank authors for conducting the new experiments and including additional clarifications. I have a remaining confusion regarding the new "Compartment-resolved signals within important regions" results in Supp. Table 10. For example: high CD3 (T cell marker) is observed to have big difference in important stromal regions between worse- and better-survival patients. Should I interpret this as: (1) T cells in worse-prognosis patients are expressing more CD3? (2) Worse-prognosis patients had more stromal T cells? (3) Important stromal regions labeled (by SpaCEy) in worse-prognosis patients contain higher proportions of T cells? On top of this, would there be any therapeutic insight that we can acquire here, such as what kind of perturbations on T cells can potentially improve the survival for a worse-prognosis patient?

R1.6: Thank authors for all the additional discussion. There are a few remaining questions I want to clarify:
- Maybe I have missed it, the "FoxP3 marker signal corresponded to increased Treg abundance" part is not found in the revised manuscript;
- "49.9% of pooled important Tc cells (3,019 of 6,053 cells) and 40.9% of pooled important Th cells (4,416 of 10,803 cells) in the no-progression group were cancer-adjacent". On this note, how do these numbers compare to the global (or non-important Tc & Th) statistics?
- "Th cells, CD4+ T cells and CD8+ T cells". Just want to clarify here that CD4+ T cells and CD8+ T cells are part of the source cell type labels? They don't seem to appear in Fig. 2d.

R1.8/1.9: Thank authors for the additional experiments and ablation studies. Could you please also include some display items (supp figures, tables, etc.) to support the results? For example, I would love to see highlighted important intra-tumoral CD8 nodes in a real example for ablation study 3.

(Remarks on code availability)

Reviewer #2

(Remarks to the Author)
The co-reviewer and I thank the authors for their extensive efforts in revising the manuscript and for providing detailed responses in the rebuttal. The inclusion of Kaplan–Meier curves (Figs. 2a, 3a, 4a), the Cox proportional hazards baseline (Fig. 5), the in silico node-ablation experiments, and the cross-batch transfer analysis using the CODEX colorectal cancer cohort (Section 2.7) are acknowledged as substantial technical additions.
Despite these extensive updates, the core methodological and conceptual concerns regarding the "added value" of SpaCEy's spatial framework and the "circularity" of downstream patient stratification remain fundamentally unresolved. The current analytical logic still suffers from an "information leak" where the model is evaluated on its ability to reconstruct the very signal it was trained to optimize.
More specifically:

Major Concerns
1. Unresolved Circularity in Cluster-Based Stratification and Proof of "Added Value."
In the rebuttal, the authors emphasize that they replaced the original survival boxplots with Kaplan–Meier (KM) curves and log-rank tests (Figs. 3b/3g and 4b/4e) to demonstrate patient stratification. However, this does not resolve the circularity problem highlighted in the first review.
The Core Invalidation: SpaCEy is explicitly trained using survival/progression data as a supervised target. Therefore, the resulting sample-level embeddings are mathematically forced to separate patients based on clinical outcomes. Applying Leiden clustering to these survival-optimized embeddings to generate KM curves is essentially a "re-readout" of the training target. It is statistically guaranteed that these clusters will show a significant log-rank p-value.
Lack of Baseline Control: A trivial, non-spatial baseline model (e.g., a simple supervised PCA/PLS or a standard MLP trained purely on pseudobulk cell-type proportions, with the survival label shown during training) would produce the same qualitative KM separation. The authors have not yet proven that spatial topology or the GNN architecture adds any unique predictive power or biological insight to this stratification beyond what a non-spatial supervised baseline achieves.
Inconsistency in LUAD Dataset (Figure 2c): As noted previously, Figure 2c merely compares marker abundance within "important nodes" between predefined progression and non-progression labels. Since the model was explicitly trained to separate these two groups, recovering these differences is fully expected and could be achieved via a standard differential test on raw bulk/single-cell data.
Therefore, to validate that SpaCEy provides true "added value," the authors must perform at least one of the following:
(1) Run the exact same Leiden clustering and KM pipeline on a non-spatial supervised baseline to prove that SpaCEy's spatial embedding yields a significantly sharper or qualitatively distinct stratification.
(2) Test the learned stratification framework on an independent external validation cohort where survival data were completely withheld during the training phase.
(3) Apply unsupervised clustering to the LUAD embeddings to see if SpaCEy uncovers novel, clinically meaningful subgroups that go beyond the sub-optimal progression labels ($p = 0.0767$).

2. Contradiction Regarding the "Annotation-Free" Paradigm

The manuscript (including the Abstract and Introduction) continues to market SpaCEy as an "annotation-free" tool that models tissues "without requiring predefined cell types." Yet, looking at the revised downstream analyses, the biological narrative depends almost entirely on cell-type annotations.
The Contradiction: The cell-type-proportion analyses of important nodes (Figs. 2d, 3d, 4d), the new compartment-stratified marker analyses (tumor/stroma/immune/vessel; Supp. Tables 10–11), the cell-type overlays (Fig. 3f), and the cancer–endothelial adjacency discovery (Fig. 2e) all fundamentally require pre-existing cell-type labels. Furthermore, the preprocessing pipeline (Section 4.1) explicitly serializes cell-type annotations.
Therefore, the claim of being "annotation-free" is factually inconsistent with the actual workflow supporting the paper's biological conclusions. The authors should either soften this claim throughout the manuscript (framing it as "cell-type annotation-agnostic during embedding feature-extraction, but annotation-dependent for biological interpretation") or demonstrate that major clinical insights can be derived entirely without cell-type maps.

3. Missing Architectural Specifications (Graph-Level Readout)
While Section 4.2 has been updated to describe the message-passing layers and fully connected layers, it still completely omits the graph-level readout (pooling) operation.
The Issue: A spatial graph contains a variable number of nodes (cells) per tissue sample. To pass this into fully connected layers for sample-level survival prediction or sample-level Leiden clustering, the variable node embeddings must be aggregated into a fixed-length vector. Whether the authors used mean, sum, max, or attention-based pooling is a critical architectural choice that affects both performance and reproducibility.
Thus, the authors should explicitly state the mathematical formulation of the graph-level readout operation in Section 4.2 and state whether different pooling strategies were benchmarked.

4. Direct Visual Evidence for Cross-Batch Robustness
The addition of the CODEX colorectal cancer cohort (Section 2.7) for cross-batch transfer analysis is a step in the right direction. However, asserting that SpaCEy is "robust to batch effects" based purely on predictive accuracy metrics is insufficient.
Thus, the authors should provide a UMAP or t-SNE plot of the learned sample-level embeddings for the CODEX dataset, colored side-by-side by (1) technical batch identity and (2) clinical/immune phenotype. To claim technical robustness, the visualization must clearly demonstrate that samples are grouped by biological signals rather than clustering by the technical batch of origin. Additionally, the text should clarify why a colorectal dataset was used for cross-batch validation of a model largely showcased on breast and lung cancers, and note whether marker panel mismatches limited a breast-to-breast cross-batch transfer.

Minor Issues and Technical Corrections
1. Incomplete Sentence in Section 2.4: The text currently reads: "The elevated expression of fibronectin in the lower-survival HR+HER2- patients points to [44]." The sentence cuts off abruptly after the citation. The actual scientific claim or conclusion must be explicitly filled in.

2. Nomenclature Standardization: Technical terms and dataset names remain inconsistent. Please standardize "Space-GM" vs. "SPACE-GM" and "JacksonFischer" vs. "Jackson-Fischer" throughout the text.

3. Missing Units: In Supplementary Table 5, the column "Peak GPU Memory" lists numerical values (e.g., 235.18) without any units. Please specify "MB" or "GB" in the column header.

4. Figure Caption Clarification: For Figure 2f, the caption must explicitly define what the error bars represent (e.g., Standard Deviation or Standard Error of the Mean).

(Remarks on code availability)

Reviewer #3

(Remarks to the Author)
I co-reviewed this manuscript with one of the reviewers who provided the listed reports. This is part of the Nature Communications initiative to facilitate training in peer review and to provide appropriate recognition for Early Career Researchers who co-review manuscripts.

(Remarks on code availability)

Version 2:

Reviewer comments:

Reviewer #1

(Remarks to the Author)

Thank authors for the additional clarifications. I have no further questions or concerns.

(Remarks on code availability)
The code base seems well-organized with a clear README.

Reviewer #2

(Remarks to the Author)
Both the early-career co-reviewer and I checked the revised manuscript and confirmed that all the raised points are reasonably addressed.

(Remarks on code availability)

Reviewer #3

(Remarks to the Author)
I co-reviewed this manuscript with one of the reviewers who provided the listed reports. This is part of the Nature Communications initiative to facilitate training in peer review and to provide appropriate recognition for Early Career Researchers who co-review manuscripts.

(Remarks on code availability)

Open Access This Peer Review File is licensed under a Creative Commons Attribution 4.0 International License, which permits use, sharing, adaptation, distribution and reproduction in any medium or format, as long as you give appropriate credit to the original author(s) and the source, provide a link to the Creative Commons license, and indicate if changes were made.
In cases where reviewers are anonymous, credit should be given to 'Anonymous Referee' and the source.
The images or other third party material in this Peer Review File are included in the article's Creative Commons license, unless indicated otherwise in a credit line to the material. If material is not included in the article's Creative Commons license and your intended use is not permitted by statutory regulation or exceeds the permitted use, you will need to obtain permission directly from the copyright holder.
To view a copy of this license, visit https://creativecommons.org/licenses/by/4.0/

Reviewers' comments:

We thank the reviewers for their careful evaluation of our manuscript and for their constructive feedback. Below the response to reviewers' comments; their comments are in black, our response in blue, and new text is *"quoted in blue"* (and in blue in updated manuscript).

Reviewer #1:

In this manuscript, Rifaioglu et al. (the authors) have proposed a new graph neural network-based tool: SpaCEy to encode spatially-resoled single-cell omics data and use the model to predict clinical outcomes including tumor progression and overall prognosis. The authors have further demonstrated how this graph neural network can be coupled with a interpretability tool (GNNExplainer) to highlight "important" contributing regions to the predictions and showed how analysis of these important regions can reveal biologically meaningful insights. The overall motivation and design of the approach is well-presented; my main concern is that the biological interpretation results, which are highlighted as the main contributions in the introduction section, are a bit underwhelming as these insights are not really spatially-resolved and haven't fully utilized the potential of the input datasets. There are a few other points that can be improved or presented better.

We thank the reviewer for their comments. We agree that the biological interpretation should be broader and better explained. We now clarify how spatial graphs, cell-level marker abundances, and clinical labels are used in the model. We replaced survival boxplots with Kaplan–Meier analyses where appropriate. We added crop-sensitivity and scalability analyses to address ROI and field-of-view effects. We included CODEX-based analyses to assess performance across batches, and expanded the biological interpretation, compartment-stratified marker summaries, spatial overlays, and targeted ablation analyses.

R1.1) There are many places where authors presented box plots of survival length (Fig2a, 3a, 3g, 4a, 4e), which I believe is not the standard approach. Due to the presence of right-censored data, these box plots could be misleading, please use KM curves instead.

RESPONSE: We revised the affected survival visualizations to use Kaplan-Meier curves with censoring marks and assessed group differences using log-rank tests. In the revised manuscript, Fig. 2a, Fig. 3a and Fig. 4a were replaced accordingly, and we added a Methods description. We also clarified in the revised manuscript that our survival objective uses spatial graphs with cell-level marker values as node features, together with event or censoring time and an event indicator.

ACTION:

- We added the relevant survival plots to show Kaplan–Meier estimates with censoring marks and have reported the corresponding global log-rank tests in both the figure panels and captions. We have also updated the Results text for Fig.

2a, Fig. 3a, and Fig. 4a to clarify the survival comparisons by progression status or clinical subtype and to report the associated log-rank p-values.

Results text for Fig. 2a:

"Fig. 2a shows Kaplan–Meier estimates of overall survival stratified by progression status, with censoring marks indicated on each curve, revealing no significant difference in overall survival between the two progression groups (log-rank p = 0.0767). "

Caption text for Fig. 2a:

"Kaplan–Meier estimates of overall survival for patients stratified by progression status, with censoring marks shown on each curve and the global log-rank test reported in the panel"

Results text for Fig. 3a:

"For the JacksonFischer dataset, Kaplan–Meier curves (Fig. 3a) stratified by clinical subtype (HR+HER2+, HR+HER2-, HR-HER2+, and TripleNeg) showed significant differences among clinical subtypes in overall survival (log-rank p = 0.012)"

Caption text for Fig. 3a:

"Kaplan–Meier estimates of overall survival across clinical subtypes in the JacksonFischer cohort, with censoring marks shown on each curve and a global log-rank test"

Results text for Fig. 4a:

"For the METABRIC imaging cohort, Kaplan–Meier curves stratified by clinical subtype (HR+HER2+, HR+HER2-, HR-HER2+, and TripleNeg) also showed significant differences among clinical subtypes in overall survival (log-rank p = 0.0175)."

Caption text for Fig. 4a:

"Kaplan–Meier estimates of overall survival across clinical subtypes in the METABRIC imaging cohort, with censoring marks shown on each curve and a global log-rank test "

- In addition, under Section "4.3 Training, Validation and Test Settings", we added the following text to clarify the objective function used to optimise the survival prediction models and the use of clinical data:

"For classification tasks, we used the clinical outcome labels available for each dataset, such as binary progression status, whereas survival tasks used time-to-event outcomes together with censoring status. Other clinical metadata were retained for descriptive analyses and downstream interpretation but were not used as node features or direct model inputs."

- A new subsection titled "4.4 Survival Analysis" was added under the Methods section.

"4.4 Survival Analysis

For descriptive survival comparisons across clinical groups, we performed patient-level Kaplan–Meier analysis using the observed overall survival time and the event indicator provided in the clinical metadata. In datasets with repeated images or cores per patient, records were collapsed to a single patient-level entry before survival estimation to avoid duplicate patient contributions. Survival curves were estimated separately for each clinical group, and differences among groups were assessed using an omnibus log–rank test. This test compares, at each observed event time, the number of events observed in each group with the number expected under the null hypothesis that all groups share the same survival distribution; these deviations are then aggregated across event times to obtain a global test statistic and corresponding p-value. Censoring times were indicated directly on the Kaplan–Meier curves."

R1.2) From what I understand, the UMAP and clustering were performed using embeddings of the whole regions (ROIs). This is different from classic approach (e.g., Cellular Neighborhood) or SPACE-GM (i.e., k-hop subgraphs), as SpaCEy embeddings have aggregated information across all cells from the ROIs. However, selection of the ROIs happened before computational analysis and introduced human sampling bias. In other words, neighborhoods or k-hop subgraph approaches ensure full knowledge for each data point (since they focus on smaller sub-regions within the ROI), while SpaCEy embedding is subject to partial knowledge: areas outside the ROIs could contain more important information but are now excluded due to experimental sampling. My concern is how such sampling bias, or edge effect, could influence the embeddings and their interpretations. For example, if during inference we only use the center crops (or any crop, such as top left corner) of the input regions, how would results change?

RESPONSE: We performed an inference-only crop-sensitivity ablation analysis (2.9 Ablation and Sensitivity Analyses) on the JacksonFischer dataset using the trained survival model. Specifically, for each ROI, we compared the full graph against systematic 50% center and corner crops, a stringent perturbation chosen to approximate partial-field-of-view sampling while still preserving sufficient tissue context for inference, and quantified the stability of predicted risk, latent embeddings, cluster assignments, and explanation-derived important regions. We revised the manuscript to state that SpaCEy shows moderate robustness to partial field of view, but that ROI restriction remains a limitation that can influence prediction, embedding structure, and interpretation.

ACTION:

- We added a new subsection titled "Ablation and Sensitivity Analyses":

"2.9 Ablation and Sensitivity Analyses

To assess the contribution of individual SpaCEy components and evaluate the sensitivity and robustness of the model, we performed a series of ablation studies focusing on key architectural and input-design choices. To assess the sensitivity of SpaCEy to partial field of view, we performed an inference-only crop-sensitivity analysis on the JacksonFischer dataset using the trained survival model. Each full region of interests (ROIs) was compared against five fixed 50% crops defined from the ROI bounding box (center, upper-left, upper-right, lower-left, and lower-right). Across full ROIs and 1,578 valid crop comparisons, crop-based predictions remained highly concordant with full-ROI predictions (mean Spearman ρ = 0.943), while risk-tertile agreement remained 0.823 on

average. Here, risk-tertile agreement means that the crop and its parent full ROI were assigned to the same low/mid/high risk tertile using cutpoints defined from the full-ROI risk-score distribution. Crop embeddings also remained highly similar to the corresponding full-ROI embeddings (mean cosine similarity 0.920). We further assessed whether crop-derived embeddings preserved the latent-space cluster assignment of the corresponding full ROI. In this analysis, full-ROI embeddings were clustered globally with Leiden, and each crop embedding was assigned to the nearest full-ROI cluster centroid by cosine similarity with the aim of measuring whether the crop remains in the same full-ROI embedding cluster as its parent ROI. On a stratified explanation subset of full ROIs and valid crop explanations, the cell-type composition of important nodes was stable (mean Pearson correlation 0.83–0.97). The cell-type composition correlation was calculated by restricting the full-ROI explanation to cells present in the crop, converting the important nodes from the restricted full ROI and crop explanation into normalized cell-type proportion vectors and computing the Pearson correlation between the two vectors. These results indicate that SpaCEy is moderately robust to partial observation, but ROI restriction still remains an important limitation for both prediction and interpretation. The crop-level prediction, embedding, and explanation-stability summaries are shown in Supplementary Table 7."

Supplementary Table 7. Crop-sensitivity summary for the JacksonFischer dataset. Risk-tertile agreement denotes the fraction of crops assigned to the same low/mid/high risk tertile as their parent ROI. Embedding-cluster agreement denotes the fraction of crops assigned to the same full-ROI Leiden embedding cluster as their parent ROI.

Crop Type	*Number of valid samples*	*Spearman correlation*	*Risk-tertile agreement*	*Mean cosine similarity (embeddings)*	*Cluster Agreement*	*Mean cell-type composition correlation*
Center	*319*	*0.945*	*0.818*	*0.974*	*0.812*	*0.830*
Upper-left	*316*	*0.944*	*0.826*	*0.928*	*0.848*	*0.870*
Upper-right	*311*	*0.935*	*0.817*	*0.918*	*0.83*	*0.880*
Lower-left	*315*	*0.898*	*0.798*	*0.875*	*0.765*	*0.812*
Lower-right	*317*	*0.943*	*0.817*	*0.903*	*0.833*	*0.922*

- We also added below text to the "Discussion" section

"Our sensitivity analysis showed that SpaCEy predictions, embeddings, and cell type compositions were largely stable under partial field-of-view sampling, but also confirmed that ROI restriction

remains a relevant limitation because local crops may not fully capture the tissue context available in the complete ROI."

R1.3) Further to point #2, authors mentioned in the methods that "images with cell counts exceeding the median were divided into four spatial quadrants", which seems to me a very ad hoc solution. I recommend a more systematic way to define field-of-view for each data point in the embedding. Furthermore, it is increasingly common to have whole-slide tissue imaging data from spatial omics (CODEX, Xenium, MERFISH, etc.), how would SpaCEy handle that type of data as it could be 1000 times larger than the ones processed in this work?

RESPONSE: We would like to first clarify that, in the original pipeline, the quadrant split was used only as a practical data augmentation step for large ROIs, not as the conceptual unit of analysis. We next addressed the reviewer's concern along three complementary axes. First, we added a dedicated crop-sensitivity analysis (response R1.2) in the JacksonFischer cohort using crops defined from the ROI bounding box rather than the previous quadrant heuristic. This showed that SpaCEy is robust to partial field of view, but that partial observation can still alter risk stratification, cluster assignment, and explanation overlap in a subset of samples. Second, to demonstrate SpaCEy's performance on an independent multiplex imaging dataset generated with a different multiplex imaging technology (CODEX), we added a patient-stratified analysis, using a patient-level training-validation-test split without quadrant-based data augmentation. Finally, we also performed a scalability analysis based on a simulated dataset to show SpaCEy's applicability to whole-slide tissue imaging data.

ACTION:

- A new subsection has been added to the analysis:

"2.7 SpaCEy learns predictive spatial representations from CODEX data and retains signal across batches

To evaluate the robustness of SpaCEy representations under batch variation, we performed a batch-focused analysis using a colorectal cancer cohort, measured with CODEX, comprising 140 tissue regions from 35 patients [41]. This dataset provides a same-panel setting in which cross-batch transfer can be assessed directly, avoiding the marker-panel incompatibility that limits comparisons across some spatial proteomics datasets. We used this dataset for two related purposes: first, to test whether SpaCEy can learn predictive spatial representations from CODEX data, and second, to evaluate its sensitivity to batch effects under a controlled same-panel transfer setting.

The prediction task was formulated as a supervised binary classification problem using immune phenotype annotations, distinguishing Crohn's-like reaction (CLR) from diffuse inflammatory infiltration (DII). We used a patient-stratified 80/10/10 train/validation/test split, ensuring that no patient appeared in more than one split. After hyperparameter optimisation, we repeated the selected model configuration across five random seeds while keeping the patient-level split fixed. Across these five runs, SpaCEy achieved a held-out test AUPRC of 0.846 ± 0.026. These results indicate that SpaCEy can be trained on an independent CODEX multiplex imaging cohort while preserving patient-level separation between training, validation, and test sets.

We next assessed batch sensitivity using the matched tissue microarray batches A and B from the same CODEX cohort. Because each patient was represented in both batches, a naive batch holdout would leak patient information across splits. We therefore separated train/validation and test partitions at the patient level. Specifically, the model was trained and validated on batch A and evaluated on held-out samples from different patients in batch B. This A-to-B transfer setting is more stringent than the randomly patient-stratified analysis above, because the model is exposed during training only to the source batch and must generalize to samples acquired in a different batch.

SpaCEy retained strong predictive performance on held-out batch B in this cross-batch setting, achieving an AUPRC of 0.801 ± 0.016 across random seeds. As expected, performance on held-out batch was slightly lower than in the randomly patient-stratified analysis, consistent with the additional challenge imposed by batch transfer. These results suggest that SpaCEy preserves predictive signal across CODEX batches, while also showing the limited effect on the performance on transfer learning."

- We also extended the scalability analysis to simulate whole-slide image-scale datasets. The details of this additional analysis are provided in our response to R1.15.

R1.4) This paper highlights SpaCEy's capabilities in identifying and summarizing key biological insights from modeling spatially-resolved data. However, most of the interpretation results are like: "In Cluster 0 (higher survival), we observed higher localized expression of PR while Cluster 2 (lower survival) showed an enrichment of fibronectin, β-catenin and CK19." These results are far from spatially-resolved (more examples in point #6 below). For example, fibronectin expression in CAF versus Cancer cells versus endothelial cells (intratumoral or extratumoral) could all have different indications. These current biological results are too broadly-defined and can hardly be translated into a verifiable hypothesis to allow further investigation.

RESPONSE: We now added a context-specific analysis of important nodes in the breast cancer cohorts. In the JacksonFischer dataset, important nodes were stratified using the available broad class annotation: Tumor, Stroma, Immune, and Vessel. In the METABRIC dataset, the available cell type annotations were mapped to broad tumor, stromal, immune, vessel, and other compartments. The unit of analysis was the image/ROI-level mean marker abundance among important nodes, calculated separately within each broad cellular context. We therefore revised the manuscript to report the tissue compartments in which important nodes carrying these marker signals are localized. These analyses provide cellular-context-resolved summaries of marker signals in important regions. They do not, however, establish definitive intratumoral versus extratumoral localization in every dataset due to differing annotations and spatial compartment definitions across cohorts. These changes make the biological interpretation spatially and cellularly constrained, while avoiding overinterpretation beyond the resolution supported by the available annotations.

ACTION:

- In the JacksonFischer Results section, we added:

"To determine whether survival-associated marker signals were localized to specific tissue compartments rather than reflecting global tissue-wide changes, important nodes were first separated into broad cellular compartments, including tumor, stroma, immune, and vessel. Marker abundance was then summarized as the sample-level mean within each compartment (Supplementary Table 10). In the JacksonFischer cohort, Ki-67 had its strongest lower survival shift in tumor-context important nodes, whereas CD3, Slug, and c-Myc had their strongest lower survival shifts in stromal-context important nodes. For each marker, the reported compartment corresponds to the context with the largest positive standardized mean difference between lower- and higher-survival samples. We therefore interpret these markers as compartment-resolved signals within important regions rather than as global tissue-wide marker changes or direct evidence of cell-intrinsic mechanisms."

"Finally, we assessed the composition of important nodes across the JacksonFischer dataset and found that they were heterogeneous across tissue compartments, with mean proportions of 55.2% tumor, 29.3% stroma, 13.5% immune, and 2.0% vascular compartments. This indicates that identified regions by the explainer are not confined to a single compartment, but instead capture outcome-associated local tissue structures spanning mainly tumor and stromal contexts, with additional immune and vascular contributions."

Supplementary Table 10. Context-stratified marker shifts in JacksonFischer SpaCEy-important nodes. Cells were first grouped by broad cellular context, and marker abundance was summarized as the ROI-level mean among important nodes within each context. Positive lower-minus-higher survival differences indicate higher abundance in lower-survival ROIs. The strongest lower-survival-associated context was defined as the context with the largest positive standardized mean difference (SMD) for each marker.

Marker	*Strongest context*	*SMD*
Ki-67	*Tumor*	*0.325*
CD3	*Stroma*	*0.421*
Slug	*Stroma*	*0.462*
c-Myc	*Stroma*	*0.507*

- In the METABRIC Results section, we added:

" Across METABRIC samples, important nodes were also compositionally mixed, with mean important-node proportions of 58.0% tumor, 30.4% stroma, 9.0% immune, 1.6% vessel, and 1.0% unclassified regions."

R1.5) Interpretation results of SpaCEy were based on "important" nodes, which by itself is a very vague and variable concept (according to Fig3e). What if tumoral areas are important in one region and stromal areas are important in another region? Then during comparison stromal biomarkers (Vimentin, aSMA, fibronectin) will surely appear (and they did appear in Fig4c), but can you conclude that more stromal areas mean better/worse prognosis? It is unclear to me how much of that is due to sampling bias (point #2 above) and how much is actually biologically meaningful? In other words, it is hard to do apple-to-apple comparison in this setting, because we don't have a consistent understanding about what are the "important" regions. Comparing tumor against stroma from different patients, for example, is not typically an experiment that will yield valuable results.

RESPONSE: We agree that important nodes can vary across patients and tissue contexts, and that they should not be interpreted as belonging to a fixed biological compartment, as also discussed in R1.4. We therefore define important nodes operationally as tissue regions selected by the explainer as informative for the clinical outcome of interest. These regions are model-informative because they contain spatial or molecular features that help separate outcome groups, but they should not be treated as directly equivalent to predefined histological compartments or as evidence of causal mechanisms. To address this point, we quantified the cellular composition of important nodes at the ROI level. We also directly addressed the reviewer’s concern that stromal-associated marker signals could simply reflect a higher stromal fraction among important nodes by comparing compartment proportions between lower- and higher-survival groups. This analysis showed that lower-survival samples did not have a higher stromal fraction among important nodes. We therefore now describe these markers as context-dependent signals within heterogeneous important regions. Where annotations allow, we interpret marker shifts within broad cellular contexts rather than as global tissue-wide changes. We also revised the manuscript to clarify that statements such as “lower-survival patients show elevated marker X” refer to elevated marker abundance within important regions associated with lower-survival samples, not to a direct patient-level causal claim or to the entire tissue section.

ACTION:

- We added clarification text to the Results Section 2.2 to define what we mean by important nodes and how they were used in the downstream analyses.

“Here, important nodes refer to a group of cells selected by the explanation module as part of model-informative spatial regions.”

- In addition, under section 2.3, we added the following clarification text to also indicate the selected regions are heterogeneous.

“These regions are not restricted to a single biological compartment, but may include tumor, stromal, immune, and vascular cells. Next, we identified spatially contiguous protein expression patterns that distinguished high- and low-survival clusters in the overall dataset (Fig. 3c).”

- We added the JacksonFischer important-node composition summary to clarify that important regions are compositionally heterogeneous and are not equivalent to a fixed tissue compartment.

"Finally, we assessed the composition of important nodes across the JacksonFischer dataset and found that they were heterogeneous across tissue compartments, with mean proportions of 55.2% tumor, 29.3% stroma, 13.5% immune, and 2.0% vascular compartments. This indicates that identified regions by the explainer are not confined to a single compartment, but instead capture outcome-associated local tissue structures spanning mainly tumor and stromal contexts, with additional immune and vascular contributions. Given this mixed composition, we focused on the stromal compartment by quantifying the cellular composition of important nodes at the sample level. To directly assess whether stromal-associated marker signals were driven by a higher stromal fraction among important nodes, we compared proportions of important nodes per compartment between lower and higher-survival groups. In JacksonFischer, lower-survival samples showed a lower stromal fraction of important nodes than higher-survival samples (27.7\% vs. 30.9\%; Mann--Whitney \(p = 0.018\)), indicating that stromal marker enrichment should not be interpreted as evidence that a larger stromal area alone explains the lower-survival phenotype. Rather, these markers represent marker-level signals within heterogeneous important regions, which we further interpret using context-stratified marker summaries where annotations are available."

- In the METABRIC Results section, we added the corresponding METABRIC composition summary:

"Across METABRIC samples, important nodes were also compositionally mixed, with mean important-node proportions of 58.0% tumor, 30.4% stroma, 9.0% immune, 1.6% vessel, and 1.0% unclassified regions. Similarly, in METABRIC, stromal important-node fractions were comparable between lower- and higher-survival ROIs (30.0% vs 30.8%; p = 0.263)."

R1.6) Overall, the biological interpretations in this manuscript are very broad/vague and need further clarification or investigation:

- Fig2c shows more FoxP3, but are there more Tregs? Are are the Tregs located in tumor, or in stroma? Fig2d suggested more Tc and Th in "No progression" group, are they found in the tumor?

- In page 5, what are CD4+/CD8+ T cells? I don't think these are commonly recognized.

- Fig3c states that high CD3 is relevant to worse prognosis, which is counter-intuitive. Where are all the T cells located? Are they in the tumor or stroma? What can we know about these idle T cells?

- Ki67 appears in Fig3c, which is a hallmark proliferation marker. It will appear in both tumor cells and immune cells. Which ones are important nodes here?

- Authors stated: "Ki-67, a widely used proliferation marker, indicates aggressive tumor growth, and elevated Ki-67 levels are associated with poor survival in breast cancer". I believe certain therapies are also more effective for patients with stronger Ki-67, to say that Ki-67 level is unconditionally associated with poor survival in breast cancer could be factually incorrect.

- Fig4d suggests fibronectin, vimentin, bCatenin, SMA as differentially-expressed biomarkers, and myofibroblast and fibroblast as key cell type differences at the same time. Once again, is it because cluster 2 has more stromal areas labelled as important? If yes, do you think that the ratio of stromal area is a strong indicator of prognosis?

- Authors' interpretations are: "A high abundance of vimentin, a well-known marker of EMT, is associated with enhanced migratory and invasive behaviour in breast cancer cells, promoting metastasis." How would you know that the signal of Vimentin here is coming from cancer cells but not stromal cells (which commonly express Vimentin)?

RESPONSE: We updated the biological statements as a result of more detailed interpretation. First, for the LUAD-specific Fig. 2 question, we checked whether the FoxP3 marker signal corresponded to increased Treg abundance and whether important Tc/Th cells were located near tumor cells. We used the explanations together with the LUAD cell-type labels and graph edges. Because the LUAD annotations do not define explicit stromal-region boundaries, we used direct adjacency to cancer cells as a conservative proxy for tumor localization and described the remaining cells as nontumor-adjacent rather than stromal. For the no-progression-associated T-cell signal, direct tumor adjacency was defined as a graph edge connecting an important immune cell to at least one cancer cell. Using this definition, 49.9% of pooled important Tc cells (3,019 of 6,053 cells) and 40.9% of pooled important Th cells (4,416 of 10,803 cells) in the no-progression group were cancer-adjacent, indicating that the signal is partly tumor-localized. We also revised the breast-cohort interpretations so that marker claims are tied to the cellular contexts where the signals are observed. We also changed the Ki-67 statement to "generally associated with worse survival" and avoided assigning vimentin/fibronectin signals to cancer-cell EMT unless the context-specific analysis supports that localization.

ACTION:

- We added the below text to clarify where the Tc and Th cells are localised in non-progressor samples

"To assess whether this immune-cell signal was located in direct proximity to tumor cells, we defined tumor adjacency as a direct graph edge between an important immune cell and at least one cancer cell. Using this definition, 49.9\% of pooled important Tc cells (3,019 of 6,053 cells) and 40.9\% of pooled important Th cells (4,416 of 10,803 cells) in the no-progression group were cancer-adjacent, indicating that this no-progression-associated T-cell signal is partly tumor-localized."

- We corrected the wording of T cells in the mentioned sentence.

"Th cells, CD4+ T cells and CD8+ T cells play key roles in anti-tumor immunity, whereas B cells have previously been associated with improved overall survival in LUAD."

- We revised the Ki-67 sentence under section 2.3 to: "elevated Ki-67 levels are generally associated with worse survival in breast cancer."

"To determine whether survival-associated marker signals were localized to specific tissue compartments rather than reflecting global tissue-wide changes, important nodes were first separated into broad cellular compartments, including tumor, stroma, immune, and vessel. Marker abundance was then summarized as the sample-level mean within each compartment (Supplementary Table 10). In the JacksonFischer cohort, Ki-67 had its strongest lower survival shift in tumor-context important nodes, whereas CD3, Slug, and c-Myc had their strongest lower survival shifts in stromal-context important nodes. For each marker, the reported compartment corresponds to the context with the largest positive standardized mean difference between lower- and higher-survival samples. We therefore interpret these markers as compartment-resolved signals within important regions rather than as global tissue-wide marker changes or direct evidence of cell-intrinsic mechanisms."

"Ki-67, a widely used proliferation marker, indicates aggressive tumor growth, and elevated Ki-67 levels are generally associated with worse survival in breast cancer [45, 34]."

- We also revised the CD3 interpretation to:

"Notably, detecting CD3 in lower survival patients in the overall dataset suggests heterogeneity in immune cell infiltration, with interpretation depending on whether CD3-positive cells are located in tumor or stromal important regions."

- For the METABRIC dataset, we revised the relevant text to avoid interpreting vimentin as an unqualified EMT marker. Instead, we now describe fibronectin, vimentin, beta-catenin, and SMA as important-region marker signals and explicitly state that their biological interpretation depends on the cellular context in which they are detected.

R1.7) The generalization experiment between JacksonFischer and METABRIC can be done by training on one dataset and testing on the other, to show both quantitative prediction performances and interpretation results.

RESPONSE: A fully joint JacksonFischer/METABRIC analysis is not straightforward, because the two cohorts do not share the same marker panel. Direct pooling or cross-cohort transfer would require reducing both datasets to a shared marker subset and redefining the input space. To address the reviwer's comment, we performed a separate batch-transfer analysis on a CRC CODEX dataset as a surrogate. In the

batch-transfer analysis, training and validation were performed on one microarray batch, while the other batch was held out as the test set. We discuss this analysis and action points in more detail in the response to the comment R1.8 below.

R1.8) This is not discussed in the manuscript, but most spatial omics experiments (proteomics and transcriptomics) suffer from batch effects. One key reason behind using cell types is because discretized version of the data are more stable and less subject to batch-specific signals. How will SpaCEy deal with batch effect when studying multiple batches of examples?

RESPONSE: We agree that batch effects are an important consideration for spatial omics studies. We now added a controlled batch-transfer analysis using the CRC CODEX cohort, which contains matched TMA batches A and B measured with the same marker panel. We trained and validated SpaCEy on batch A, and evaluated it on held-out samples from batch B. This A-to-B setting is more stringent than within-batch evaluation because the model is trained and validated only on the source batch and tested on the other batch. Compared with the patient-stratified random-splitting analysis, we showed that performance was slightly lower but remained robust across random seeds.

ACTION:

- We performed a new analysis to investigate the batch-effect performance on a CODEX cohort. The text below as added under the new subsection titled: "SpaCEy learns predictive spatial representations from CODEX data and retains signal across batches"

"2.7 SpaCEy learns predictive spatial representations from CODEX data and retains signal across batches

To evaluate the robustness of SpaCEy representations under batch variation, we performed a batch-focused analysis using a colorectal cancer cohort, measured with CODEX, comprising 140 tissue regions from 35 patients [41]. This dataset provides a same-panel setting in which cross-batch transfer can be assessed directly, avoiding the marker-panel incompatibility that limits comparisons across some spatial proteomics datasets. We used this dataset for two related purposes: first, to test whether SpaCEy can learn predictive spatial representations from CODEX data, and second, to evaluate its sensitivity to batch effects under a controlled same-panel transfer setting.

The prediction task was formulated as a supervised binary classification problem using immune phenotype annotations, distinguishing Crohn's-like reaction (CLR) from diffuse inflammatory infiltration (DII). We used a patient-stratified 80/10/10 train/validation/test split, ensuring that no patient appeared in more than one split. After hyperparameter optimisation, we repeated the selected model configuration across five random seeds while keeping the patient-level split fixed. Across these five runs, SpaCEy achieved a held-out test AUPRC of 0.846 ± 0.026. These results indicate that SpaCEy can be trained on an independent CODEX multiplex imaging cohort while preserving patient-level separation between training, validation, and test sets.

We next assessed batch sensitivity using the matched tissue microarray batches A and B from the same CODEX cohort. Because each patient was represented in both batches, a naive batch holdout

would leak patient information across splits. We therefore separated train/validation and test partitions at the patient level. Specifically, the model was trained and validated on batch A and evaluated on held-out samples from different patients in batch B. This A-to-B transfer setting is more stringent than the randomly patient-stratified analysis above, because the model is exposed during training only to the source batch and must generalize to samples acquired in a different batch.

SpaCEy retained strong predictive performance on held-out batch B in this cross-batch setting, achieving an AUPRC of 0.801 ± 0.016 across random seeds. As expected, performance on held-out batch was slightly lower than in the randomly patient-stratified analysis, consistent with the additional challenge imposed by batch transfer. These results suggest that SpaCEy preserves predictive signal across CODEX batches, while also showing the limited effect on the performance on transfer learning."

R1.9) Since authors have already performed simulation studies, I think it makes sense simulating a toy dataset to first justify that GNNExplainer can correctly pick the important features that contribute to the task. For example, by predicting prognosis using a simulated TME dataset where number of tumor-infiltrating CD8 T cells (not including stromal CD8 T cells) is the only correlate of the task label, we can evaluate whether the intra-tumor CD8 T cells are being assigned higher importance scores than extra-tumoral CD8 T cells.

RESPONSE: We performed a standalone synthetic tumor-stroma analysis in which intratumoral and stromal/extratumoral CD8 counts were kept similar, while the graph label depended on whether intratumoral CD8 cells formed a compact spatial motif. This revised design controls for CD8-cell counts and tests whether the explainer preferentially highlights the intended intratumoral CD8 spatial signal rather than extratumoral CD8 cells. We showed that the explainer model consistently identifies the intratumoral CD8 cells as important cells in the spatial graphs.

ACTION:

- To address the reviewer's comment, the below text was added under "Ablation and Sensitivity Analyses" subsection based on the analysis suggested by the reviewer.

"The aim of the third ablation study was to validate the explainer model in a setting with a known ground-truth spatial signal. For this, we generated a synthetic, standalone toy tumor–stroma dataset in which positive samples contained high intratumoral CD8 abundance, whereas negative samples had matched CD8 abundance shifted to the stromal compartment. The model received only synthetic marker-channel features and spatial graphs as input; the synthetic marker channels represented tumor, CD8, stromal, immune-activation, proliferation, fibroblast/SMA-like, and noise-control signals. Simulated cell type labels (tumor cells, CD8 T cells, fibroblasts, and macrophage-like cells) and compartment labels were used only for ground-truth evaluation and post hoc interpretation, not for model prediction. The final synthetic dataset comprised 300 graphs with varying numbers of cells per graph, and a model was trained to predict the corresponding graph-level labels. When explainer model was applied to held-out test graphs, intra-tumoral CD8 nodes received higher mean importance than stromal CD8 nodes in explained graphs, with mean

group importance 0.0467 for intra-tumoral CD8 versus 0.0162 for stromal CD8 (paired one-sided Wilcoxon signed-rank p = 8.29 × 10−14), supporting that the explainer selectively highlights the intended intra-tumoral CD8 spatial motif rather than extra-tumoral CD8 cells."

R1.10) In Fig5, where authors compared SpaCEy against baseline methods, I think it is necessary to include a basic Cox regression model for the survival modeling task.

RESPONSE: We now added CoxPH baselines for both survival cohorts using the same predefined cohort splits already used for the existing baselines, rather than regenerating new splits. We evaluated CoxPH in our baseline framework using the same patient-stratified setting.

ACTION:

- Section 2.6 and Figure 5 were updated based on the CoxPH results.and a relevant citation was also added to the relevant section:

"To this end, we compared our results with four related methods: Fast Survival SVM [37], Random Survival Forest [23], Gradient Boosting Survival Analysis [36], and a Cox proportional hazards (CoxPH) model [42]."

- We also added the following text under Results based on the CoxPH results:

On the Jackson-Fischer dataset, SpaCEy achieved a mean performance of 0.720 ± 0.061, outperforming all 24 non-spatial baseline configurations evaluated through the same fixed 10-fold protocol.

For the Cox proportional hazards (CoxPH) baseline, the best-performing variant used pseudobulk marker summaries with sum aggregation and achieved a mean test C-index of 0.600 ± 0.095.

For the CoxPH baseline, the best-performing variant again used pseudobulk marker summaries with sum aggregation and achieved a mean test C-index of 0.562 ± 0.048.

R1.11. Introduction: "highlights spatially contiguous nodes and edges" needs clarification, graphs are by design discrete.

RESPONSE: The model operates on a discrete spatial graph rather than on a continuous field. In our setting, contiguity refers to graph connectivity among spatially adjacent cells after graph construction, not to continuity in Euclidean image space. We clarify this in the revised manuscript.

ACTION:

- We revised the relevant sentences in the Introduction and Results for clarification. The text now defines spatial contiguity as graph connectivity among spatially adjacent cells after graph construction. Specifically, we describe the explanations as connected sets of adjacent nodes and edges, or compact subgraphs, identified on the constructed spatial graph. The following clarification and updates were done under Introduction section:

"We present SpaCEy, an explainable method for discovering spatially important regions on the tissues associated with clinical outcomes using GNNs.

To overcome limitations of current approaches, which often produce predictive embeddings without revealing the structural or molecular features driving their predictions, SpaCEy integrates an explainer model that highlights connected sets of spatially adjacent nodes and edges responsible for its outputs by identifying compact subgraphs [57]. "

R1.12. Further to point #1, section 2.3 stated that "The objective was to predict overall survival (hereafter referred to as survival) values using this dataset", where Fig2ab only show survival lengths. This is later clarified in Methods section that the model is doing a proper survival modeling, but I would prefer this information to be revealed in the main text to avoid confusion.

RESPONSE: In SpaCEy, the survival objective is based on the regularized negative log partial likelihood, where each sample is represented by baseline features, event or censoring time, and an event indicator specifying whether the event was observed. The loss is computed only over observed events while using the corresponding risk sets, thereby explicitly accounting for censoring in survival prediction.

ACTION:

- To clarify how we model the survival, we updated the as below to Section 2.3, "SpaCEy stratifies breast cancer patients by subtype-agnostic localised spatial patterns."

"Here, our objective was to model overall survival (hereafter referred to as survival) in a censoring-aware time-to-event setting."

- We revised the Methods section and added below text for clarification:

"For classification tasks, we used the clinical outcome labels available for each dataset, such as binary progression status, whereas survival tasks used time-to-event outcomes together with censoring status."

- Model Architecture subsection was also revised

"Node importance values are computed by aggregating edge-mask values within the k-hop neighbourhood of each node, enabling the identification of compact connected subgraphs that define important spatial regions in the tissue."

R1.13. Fig2a suggested that patients with progression have overall longer OS than patients with no progression, which is counter-intuitive. I believe it makes sense to show the full KM curves and justify how the labels were determined. In addition, are the model outputs (logits, probabilities, etc.) correlated with patient risk?

RESPONSE: We replaced this panel with a Kaplan-Meier curve to resolve the presentation issue.In the censoring-aware analysis, the progression and no-progression

groups do not differ significantly in overall survival (log-rank p = 0.0767). We agree that the task definition should be stated more clearly. In the LUAD setting, SpaCEy predicts the cohort-provided progression label as a supervised classification target; the Kaplan--Meier panel is shown only as a descriptive survival reference and should not be interpreted as the model's optimization target.

ACTION:

- We clarified the task for the LUAD dataset and added the text below under subsection 2.2:

" In the LUAD cohort, we modeled progression as a supervised binary classification task using the progression annotations provided with the dataset. "

- We added the following text to the subsection "SpaCEy identifies spatially organized protein and cellular patterns predictive of tumor progression":

"Fig. 2a shows Kaplan–Meier estimates of overall survival stratified by progression status, with censoring marks indicated on each curve, revealing no significant difference in overall survival between the two progression groups (log-rank p = 0.0767). "

R1.14. If I understand correctly, GNNExplainer only assigns unsigned importance scores. How would you acquire the directionality for these important nodes? Like can we know this important node has a positive/negative impact on prognosis predictions?

RESPONSE: In the current framework, the explainer model identifies which graph components are most influential for the prediction, but does not itself assign a signed contribution to these components. Directionality is therefore inferred indirectly, for example by comparing explanations between higher-risk and lower-risk groups, or by relating marker abundance in important regions to the corresponding model outputs at the sample or group level.

ACTION:

- Below text was added the "2.1 Model Architecture and Analysis Pipeline" subsection

"Here, node and edge importance scores produced by the explainer quantify unsigned relevance magnitudes. Thus, they identify the graph components that are most influential for the model prediction, but they do not by themselves encode the direction of effect, i.e., whether a given node, edge, or spatial motif shifts the prediction toward or away from a clinical outcome of interest."

R1.15. Fig6 discusses the scalability of SpaCEy, but there is no simulation for whole-slide samples with 1M+ cells.

RESPONSE: The original Fig. 6 simulation primarily evaluated cohort-scale training across different numbers of samples and marker dimensions, rather than explicitly varying the number of cells/nodes per spatial sample. We have now introduced a tiling

mechanism to our method to handle this type of analysis for large samples, including samples with up to 1,000,000 cells. We updated Figure 6 and the results text accordingly.

ACTION:

- To further evaluate SpaCEy's scalability for large spatial samples, we performed an additional analysis, updated the corresponding figure, and added the following text to the "Scalability Analysis Results" subsection.

"To address whole-slide-scale settings directly, we updated the scalability benchmark to vary both the number of cells per sample and the number of samples. One synthetic sample was represented as a spatial point cloud (cells) with 1,500, 10,000, 50,000, 100,000, 250,000, 500,000, or 1,000,000 cells and 50 marker channels (Fig. 6d-e). Samples exceeding a specified cell-count threshold were partitioned into cell tiles and processed; the learned features from these tiles were then aggregated to generate the final sample-level representation. The calculated per-sample processing time increased from 0.014 s for 1,500-cell samples to 7.42 s for 1,000,000-cell samples. Scaling these measured per-sample times to epochs containing 50, 100, 200, 500, 1000, or 1500 samples gave estimated epoch times from 0.69s for 50 samples with 1,500 cells each to approximately 3 hours for 1500 samples with 1,000,000 cells each. The largest setting, therefore, corresponds to 1.5 billion cells per epoch. Peak allocated GPU memory remained bounded by the tile size, reaching approximately 1.06 GB, while peak resident CPU memory was approximately 1.45 GB."

R1.16. RMSE is mentioned in the Methods section, where is it used? Which task is using RMSE for metrics?

RESPONSE: Although RMSE is implemented in SpaCEy and could, in principle, be applied to continuous regression outcomes, it is correct that no such setting was included in the present study in favor of more appropriate metrics. We removed RMSE from the manuscript to avoid confusion.

ACTION:
RMSE definition was removed under the Evaluation Metrics subsection, as we do not present evaluation results based on RMSE.

Reviewer #2 (Remarks to the Author):

In this manuscript, the authors propose an explainable graph neural network (GNN)-based method (SpCEy) that extracts characteristic tissue patterns of cell organization that are correlated with clinical outcomes, given a set of spatial omics (proteome) data with outcome information. Its performance was tested using several public datasets. The possibility of discovering important spatial patterns is interesting, and this kind of approach is increasingly important. However, this work suffers from the following points:

We thank the reviewer for their comments. We have now addressed all points as outlined below. We revised the manuscript to clarify SpaCEy's scope, objective and spatial proteomics focus. We expanded the related work, provided spatial examples, replaced survival boxplots with Kaplan–Meier analyses. We performed additional clinical subtype and CODEX batch-transfer analyses. We also introduced *in silico* ablations and a synthetic ground-truth experiment to better support the functional relevance and interpretability of SpaCEy explanations.

R2.1) Although the authors write, "A key novelty of SpaCEy lies in its ability to generate interpretable spatially contiguous regions," only one such example is shown in the figure (Fig. 3f). Moreover, the generality and medical plausibility of this finding are not well investigated; how many of the positive/negative samples in the cohort contain the motif? Was it also observed in the other cohort? In this regard, it is recommended to perform a small "silico perturbation" experiment: if the model "removes" a specific identified spatial motif from the graph, does the predicted survival score change significantly?

RESPONSE: We have revised the manuscript to provide additional evidence for the spatial interpretability of SpaCEy. Specifically, we added sample graphs to the Results figures, following the reviewer's suggestion, and added a supplementary table summarizing marker overlap between the JacksonFischer and METABRIC cohorts. The important nodes identified for each sample, together with the corresponding visualizations, are also included in the deposited data.

Further, we agree that explicit perturbation evidence strengthens the interpretation of important regions. We first note that the explainer used in SpaCEy is itself perturbation-based: it learns node and edge masks by identifying graph components whose retention best preserves the model output. To address the reviewer's request more directly, we additionally performed an in silico ablation experiment on the JacksonFischer cohort. In this experiment, we progressively removed explainer-identified important nodes together with their incident edges and measured the resulting change in model prediction. As a matched control, we removed the same number of non-important nodes. This analysis was added to evaluate how SpaCEy predictions change when the model is given only a partial view of the original tissue graph. We also added a synthetic tumor-stroma validation experiment with a known ground-truth spatial signal. In this controlled setting, positive samples were defined by intratumoral CD8 abundance, whereas negative samples contained matched CD8 abundance shifted to the stromal compartment. The explainer assigned significantly

higher importance to intratumoral CD8 nodes than to stromal CD8 nodes, indicating that it recovered the intended spatial signal rather than simply highlighting CD8 abundance irrespective of localization.

Finally, we clarified the wording around "motifs." SpaCEy does not currently define discrete, named graph motifs that can be counted as present or absent in each sample. Instead, the explainer identifies important nodes and edges within each tissue graph, and the downstream analyses summarize broader trends within these important regions, including enriched tissue compartments, marker expression patterns, and associations with clinical groups. Therefore, we cannot directly quantify how many positive or negative samples contain a specific motif in the sense of a recurrent, discrete graph pattern. To avoid overstatement, we revised the manuscript to describe these findings as trends observed across important regions rather than as individually enumerated motifs. We also added context specific summaries of important nodes to better assess whether the identified signals are consistent across clinical groups and cohorts.

ACTION:

- We now added a sample to visualize the spatially contiguous regions on representative images to support the claims,in Figure 2,3 and 4 based on the results.

"To illustrate these marker level findings at the tissue level, we visualized one representative lower-survival sample from Cluster 2. SpaCEy highlighted spatially localized important regions within the tissue section, and zoomed views of these regions showed locally elevated Slug and c-Myc abundance within the model-selected subgraphs (Fig. 3c, bottom) which indicates that the associated marker signals are not only detected at the aggregate level, but are also spatially organized within important regions of individual tissue samples."

- We added a new section 2.9. Ablation and Sensitivity Analyses' to examine how SpaCEy predictions change under in silico perturbations. In addition, using a synthetic dataset with known ground-truth regions, we show that SpaCEy successfully identifies the important regions within samples.

"In a second ablation study, we asked whether explicit removal of explainer-identified important regions alters SpaCEy predictions and survival-ranking performance more strongly than removal of matched non-important regions. In the JacksonFischer cohort, we progressively removed 1%, 5%, 10%, 20%, 50%, and 80% of important nodes, together with their incident edges, and compared the performance of the trained model. Across all ablation levels, removal of important nodes produced consistently larger decreases in model performance than removal of non-important nodes and led to a stronger degradation in concordance index, indicating that identified regions had a greater effect on both individual risk estimates and survival-ranking performance. Specifically, mean decreases in model output were 0.0094 versus 0.0071 at 1%, 0.0509 versus 0.0179 at 5%, 0.0818 versus 0.0281 at 10%, 0.1034 versus 0.0455 at 20%, 0.1553 versus 0.0913 at 50%, and 0.2175 versus 0.1289 at 80% for important-node versus matched non-important-node removal, respectively. This consistent monotonic ablation pattern indicates that explainer-identified regions are more important for SpaCEy predictions than matched non-important regions, supporting the functional relevance of the highlighted nodes.

The aim of the third ablation study was to validate the explainer model in a setting with a known ground-truth spatial signal. For this, we generated a synthetic, standalone toy tumor–stroma dataset in which positive samples contained high intratumoral CD8 abundance, whereas negative samples had matched CD8 abundance shifted to the stromal compartment. The model received only synthetic marker-channel features and spatial graphs as input; the synthetic marker channels represented tumor, CD8, stromal, immune-activation, proliferation, fibroblast/SMA-like, and noise-control signals. Simulated cell type labels (tumor cells, CD8 T cells, fibroblasts, and macrophage-like cells) and compartment labels were used only for ground-truth evaluation and post hoc interpretation, not for model prediction. The final synthetic dataset comprised 300 graphs with varying numbers of cells per graph, and a model was trained to predict the corresponding graph-level labels. When explainer model was applied to held-out test graphs, intra-tumoral CD8 nodes received higher mean importance than stromal CD8 nodes in explained graphs, with mean group importance 0.0467 for intra-tumoral CD8 versus 0.0162 for stromal CD8 (paired one-sided Wilcoxon signed-rank $p = 8.29 \times 10{-}14$), supporting that the explainer selectively highlights the intended intra-tumoral CD8 spatial motif rather than extra-tumoral CD8 cells."

R2.2) Although the authors mention "The input to SpaCEy are samples ..., represented by measurements of spatially resolved omics and paired clinical observations," it is not clear whether/how the clinical observations, such as the breast cancer subtype information, were used in the learning step. On the contrary, it looks evident that clinical outcome information, such as survival values, was used in the training. Thus, it does not make sense to me that the authors compare the survival value of their unsupervised clustering results with that of the original subtypes (Figs. 3a and 3b).

RESPONSE: We now clearly state what clinical covariates are inputs versus what is used only for supervision, stratification, or post hoc interpretation. SpaCEy learns from cell-level marker-abundance features and graph topology only. Depending on the dataset, clinical outcome information was only used as a label not as the input of the model. For example, progression labels were used for the classification tasks, and time-to-event outcomes with censoring status were used for the survival predictions. Receptor-defined subtype labels and other clinical variables are retained for descriptive and downstream interpretation only. We revised the text to clarify that the Kaplan--Meier curves across clinical subtypes are descriptive patient-level cohort references, whereas the cluster-level analyses are derived from SpaCEy embeddings learned under the survival objective.

ACTION:

- We clarified the model inputs and the role of clinical variables in the SpaCEy framework:
-

"The model input consists of protein-abundance measurements at single-cell level represented as spatial graphs; clinical outcomes are used as supervised labels, while clinical annotations are only retained for descriptive and post hoc interpretation (Methods, Data preprocessing and Training settings) (Fig.1a)."

- Clarification for the subtype comparison under Section 2.3:

"We then performed unsupervised clustering after model training by applying the Leiden algorithm to the learned sample-level embeddings. Clinical subtype composition was summarized only post hoc to contextualize these embedding-derived groups:"

- We also clarified classification and survival task definitions in the "Training, Validation and Test Settings" subsection of the Methods,

"For classification tasks, we used the clinical outcome labels available for each dataset, such as binary progression status, whereas survival tasks used time-to-event outcomes together with censoring status. Other clinical metadata were retained for descriptive analyses and downstream interpretation but were not used as node features or direct model inputs."

R2.3) In the discussion, they note "The current study focuses on protein abundance data" as a limitation. However, from the title, abstract, and introduction, it is unclear what type of spatially resolved data was used. This can be very misleading for readers. Moreover, in the introduction, the authors do not cite any related work on spatial transcriptome analyses using GNNs. Considering past methods in spatial transcriptomics, the novelty of this work could be less significant.

RESPONSE: We revised the abstract, Introduction and Discussion sections to clarify that SpaCEy is applied to single-cell spatial proteomics datasets generated by multiplex imaging assays, where graph node features correspond to protein-marker abundance measurements. We also expanded the related work in the introduction and cited graph-based methods developed for spatial transcriptomics.

ACTION:

- The sentence in Abstract was updated to clarify that SpaCEy is evaluated here in the context of spatial proteomics datasets:

"We present SpaCEy (Spatial Clinical Explainability), an explainable graph neural network that uncovers organizational tissue patterns predictive of clinical outcomes in spatial proteomics datasets."

- We added this statement to the Introduction to better acknowledge related graph-based neural methods in spatial transcriptomics.

"Graph-based neural approaches have also been developed extensively for spatial transcriptomics, including methods for spatial-domain detection, representation learning, integration, and deconvolution such as SpaGCN [21], STAGATE [11], GraphST [32], DeepST [53], SEDR [54] and SpaceFlow [39]. However, these methods have largely focused on characterising tissue organisation, while important limitations remain in connecting spatial patterns to clinical outcomes and translating model predictions into biologically interpretable tissue level insights."

- We also added the below clarification text to the Discussion:

"The current study focuses on multiplex imaging-based single-cell spatial proteomics, in which node features are protein-marker abundance measurements, and therefore provides only a partial view of cellular states and interactions."

R2.4) Because the spatial graph depends entirely on the initial cell segmentation, the authors must comprehensively test its effects on model performance. A suggested analysis is a sensitivity analysis showing how the model performs when nodes are intentionally "jittered" (slightly moved) or when a percentage of cells are randomly "dropped" to simulate segmentation errors or missed detections. This would demonstrate that the framework is robust enough for real-world clinical samples with varying imaging quality.

RESPONSE: In addition to the ablation study described in response to R2.1, we performed a node-removal ablation on the JacksonFischer cohort. In this analysis, we progressively removed cells together with their incident edges and measured the resulting change in model output and concordance index. To distinguish general sensitivity to missing cells from the functional relevance of explainer-highlighted regions, we compared removal of important nodes with removal of a matched number of nonimportant nodes. Removing important nodes caused a consistently larger change in predicted risk and a larger degradation in concordance index than removing matched non-important nodes, supporting that the highlighted regions have greater functional influence on SpaCEy predictions. We added this analysis to a dedicated subsection titled "2.9 Ablation and Sensitivity Analyses."

ACTION:

- We added a new section titled "2.9. Ablation and Sensitivity Analyses"

"In a second ablation study, we asked whether explicit removal of explainer-identified important regions alters SpaCEy predictions and survival-ranking performance more strongly than removal of matched non-important regions. In the JacksonFischer cohort, we progressively removed 1%, 5%, 10%, 20%, 50%, and 80% of important nodes, together with their incident edges, and compared the performance of the trained model. Across all ablation levels, removal of important nodes produced consistently larger decreases in model performance than removal of non-important nodes and led to a stronger degradation in concordance index, indicating that identified regions had a greater effect on both individual risk estimates and survival-ranking performance. Specifically, mean decreases in model output were 0.0094 versus 0.0071 at 1%, 0.0509 versus 0.0179 at 5%, 0.0818 versus 0.0281 at 10%, 0.1034 versus 0.0455 at 20%, 0.1553 versus 0.0913 at 50%, and 0.2175 versus 0.1289 at 80% for important-node versus matched non-important-node removal, respectively. This consistent monotonic ablation pattern indicates that explainer-identified regions are more important for SpaCEy predictions than matched non-important regions, supporting the functional relevance of the highlighted nodes."

R2.5. The authors should provide more comprehensive information and a comparison of the datasets. For instance, how similar or different are the two breast cancer cohorts in terms of their features? Could the authors perform a joint analysis, including downstream analyses, to compare patients across different cohorts simultaneously? Would SpaCEy minimize technical differences between cohorts, such as batch effects?

RESPONSE: We added a supplementary table 8 to compare the markers used in METABRIC and JacksonFischer datasets. We also want to clarify that a fully joint JacksonFischer/METABRIC analysis is not straightforward because the two cohorts do not share the same marker panel, so direct pooling or cross-cohort transfer would require reducing both datasets to a shared marker subset and redefining the input space. To address the reviewer's concern, we performed a controlled batch-transfer analysis using the CRC CODEX cohort, which includes matched TMA batches A and B profiled with the same marker panel. SpaCEy was trained and validated on batch A and then tested on held-out samples from batch B. This A-to-B transfer setup provides a more stringent evaluation than within-batch testing, because the model is optimized only on the source batch and evaluated on an independent batch. Compared with the patient-stratified random-splitting analysis, performance was slightly reduced but remained robust across random seeds.

ACTION:

- We added the following text to the new subsection "2.7 SpaCEy learns predictive spatial representations from CODEX data and retains signal across batches":

"2.7 SpaCEy learns predictive spatial representations from CODEX data and retains signal across batches

To evaluate the robustness of SpaCEy representations under batch variation, we performed a batch-focused analysis using a colorectal cancer cohort, measure with CODEX, comprising 140 tissue regions from 35 patients [41]. This dataset provides a same-panel setting in which cross-batch transfer can be assessed directly, avoiding the marker-panel incompatibility that limits comparisons across some spatial proteomics datasets. We used this dataset for two related purposes: first, to test whether SpaCEy can learn predictive spatial representations from CODEX data, and second, to evaluate its sensitivity to batch effects under a controlled same-panel transfer setting.

The prediction task was formulated as a supervised binary classification problem using immune phenotype annotations, distinguishing Crohn's-like reaction (CLR) from diffuse inflammatory infiltration (DII). We used a patient-stratified 80/10/10 train/validation/test split, ensuring that no patient appeared in more than one split. After hyperparameter optimisation, we repeated the selected model configuration across five random seeds while keeping the patient-level split fixed. Across these five runs, SpaCEy achieved a held-out test AUPRC of 0.846 ± 0.026. These results indicate that SpaCEy can be trained on an independent CODEX multiplex imaging cohort while preserving patient-level separation between training, validation, and test sets.

We next assessed batch sensitivity using the matched tissue microarray batches A and B from the same CODEX cohort. Because each patient was represented in both batches, a naive batch holdout would leak patient information across splits. We therefore separated train/validation and test partitions at the patient level. Specifically, the model was trained and validated on batch A and

evaluated on held-out samples from different patients in batch B. This A-to-B transfer setting is more stringent than the randomly patient-stratified analysis above, because the model is exposed during training only to the source batch and must generalize to samples acquired in a different batch.

SpaCEy retained strong predictive performance on held-out batch B in this cross-batch setting, achieving an AUPRC of 0.801 ± 0.016 across random seeds. As expected, performance on held-out batch was slightly lower than in the randomly patient-stratified analysis, consistent with the additional challenge imposed by batch transfer. These results suggest that SpaCEy preserves predictive signal across CODEX batches, while also showing the limited effect on the performance on transfer learning."

R2.6. The authors mention colocalization of cancer and endothelial cells; however, Figure 2d shows only proportions, with no actual objective measure of colocalization. If they want to claim colocalization, they should use an approach other than proportion and provide clear examples from image data.

RESPONSE: We selected additional informative LUAD regions using a direct graph contact criterion: edges in spatial graphs connecting explainer-important cancer and endothelial cell nodes. We also updated the results section and figure caption accordingly.

ACTION:

- A panel was added to Figure 2e showing an example image that illustrates the colocalization of cancer and endothelial cells.
- The following text was added under Section "2.2 SpaCEy identifies spatially organized protein and cellular patterns predictive of tumor progression."

"To provide an image-level example, we selected a representative progressor LUAD sample and visualized direct graph contacts between explainer-important cancer and endothelial cell nodes, highlighted with bold edges (Fig.2e). This example illustrates local cancer–endothelial adjacency within an explainer-highlighted tissue region."

- Figure 2e caption was added

"e) Representative progressor LUAD sample showing important cancer and endothelial cell nodes. The zoomed region highlights direct edges in the input spatial graph between important cancer and endothelial cells "

R2.7. The authors should add significance when presenting a comparison relevant to their results, e.g., the boxplot in Figure 3b.

RESPONSE: For the survival panels that were previously shown as boxplots, we replaced those visualizations with Kaplan--Meier curves and reported log-rank test results directly on the plots. We also clarified the statistical testing used in the corresponding

captions and text. In addition, we clarified that the survival objective used to optimize the models incorporates the event or censoring time.

ACTION:

- We modified the figure legends as follows:

Results text for Fig. 2a:

"Fig. 2a shows Kaplan–Meier estimates of overall survival stratified by progression status, with censoring marks indicated on each curve, revealing no significant difference in overall survival between the two progression groups (log-rank p = 0.0767). "

Caption text for Fig. 2a:

"Kaplan–Meier estimates of overall survival for patients stratified by progression status, with censoring marks shown on each curve and the global log-rank test reported in the panel"

Results text for Fig. 3a:

"For the JacksonFischer dataset, Kaplan–Meier curves (Fig. 3a) stratified by clinical subtype (HR+HER2+, HR+HER2-, HR-HER2+, and TripleNeg) showed significant differences among clinical subtypes in overall survival (log-rank p = 0.012)"

Caption text for Fig. 3a:

"Kaplan–Meier estimates of overall survival across clinical subtypes in the JacksonFischer cohort, with censoring marks shown on each curve and a global log-rank test"

Results text for Fig. 4a:

"For the METABRIC imaging cohort, Kaplan–Meier curves stratified by clinical subtype (HR+HER2+, HR+HER2-, HR-HER2+, and TripleNeg) also showed significant differences among clinical subtypes in overall survival (log-rank p = 0.0175)."

Caption text for Fig. 4a:

"Kaplan–Meier estimates of overall survival across clinical subtypes in the METABRIC imaging cohort, with censoring marks shown on each curve and a global log-rank test "

R2.8) When describing Figure 3c, the authors refer to it as a spatially contiguous expression; however, Figure 3c is a simple dot plot. Please include relevant Figures to support your assertions.

RESPONSE: We updated Figures 3 and 4 to include representative spatial overlays with zoomed important regions, alongside the cohort-level quantitative summaries. These additions provide image-level support for the spatial-organization claims made from the marker-level analyses.

ACTION:

- We added the following text to the Results section describing Fig. 3c:

"To illustrate these marker level findings at the tissue level, we visualized one representative lower-survival sample from Cluster 2. SpaCEy highlighted spatially localized important regions within the tissue section, and zoomed views of these regions showed locally elevated Slug and c-Myc abundance within the model-selected subgraphs (Fig. 3c, bottom) which indicates that the associated marker signals are not only detected at the aggregate level, but are also spatially organized within important regions of individual tissue samples."

R2.9) In the analysis of HR+HER2- patients (Figure 3g), the main markers largely overlap with those from the whole cohort. This could indicate a bias towards this cohort in the general analysis. It would be interesting to perform a similar analysis for the other patient classes and see whether the overlap persists across all subclasses. If so, what kind of conclusions can be derived from this? Does the model capture only global differences, rather than specific ones?

RESPONSE: We performed a targeted subtype analysis restricted to the reported lower-survival markers: Slug, c-Myc, CD3, and Fibronectin. For each analysis group (all patients, HR+HER2-, TripleNeg, HR+HER2+, HR-HER2+, and the combined non-HR+HER2- group), patients were split into lower and higher-survival groups using the group-specific median overall survival. Marker abundance was summarized as patient-level mean abundance among important nodes. In the full cohort, all four reported markers showed positive lower-survival shifts and remained FDR-supported within this reported marker set. In HR+HER2- patients, all four markers also shifted in the lower-survival direction, with Fibronectin FDR-supported. Importantly, in the combined non-HR+HER2- group, all four reported markers again shifted in the lower-survival direction, with Slug, c-Myc, and CD3 FDR-supported, while Fibronectin was not significant. Individual non-HR+HER2- subtypes showed partial concordance (3/4 or 4/4 markers in the same direction), but these subtype-specific analyses were limited by small sample size and are interpreted descriptively. These results indicate that the overlap between the whole-cohort and HR+HER2- analyses is not solely explained by HR+HER2- dominance. Instead, SpaCEy captures a shared lower-survival-associated marker component across receptor-defined subtypes, while individual subtypes retain subtype-specific variation.

ACTION:

- In the JacksonFischer HR+HER2- subsection, we added:

"To test whether the overlap between HR+HER2- and the full cohort was driven by the prevalence of HR+HER2- samples, we performed a targeted subtype analysis restricted to the identified markers (Slug, c-Myc, CD3, and Fibronectin) associated with lower-survival group. For each clinical subtype, patients were split by the group-specific median overall survival. Marker abundance was then summarized as the patient-level mean abundance among important nodes (Supplementary Table 11). All four reported markers showed positive lower-survival-associated shifts in both the full cohort and the HR+HER2- subgroup. Importantly, the same lower-survival-associated trend was also observed in the other groups. In this group, Slug, c-Myc,

and CD3 were significantly enriched in lower-survival samples ($p < 0.05$), while Fibronectin showed the same direction of change but was not statistically significant. These results indicate that the marker overlap is not solely explained by HR+HER2- dominance. Rather, SpaCEy captures a shared lower-survival- associated component across receptor-defined subtypes while retaining subtype-specific variation"

Supplementary Table 11. Summary of targeted subtype analysis for reported lower-survival markers. Marker abundance was summarized as patient-level mean abundance among hard-mask SpaCEy-important nodes. Lower- and higher-survival groups were defined using group-specific median overall survival.

Analysis group	Markers higher in lower-survival group	FDR-supported markers	Mean SMD
Full cohort	4/4: Slug; c-Myc; CD3; Fibronectin	Slug; c-Myc; CD3; Fibronectin	0.39
HR+HER2-	4/4: Slug; c-Myc; CD3; Fibronectin	Fibronectin	0.29
Combined non-HR+HER2-	4/4: Slug; c-Myc; CD3; Fibronectin	Slug; c-Myc; CD3	0.47
TripleNeg	4/4: Slug; c-Myc; CD3; Fibronectin	None	0.39
HR+HER2+	3/4: Slug; CD3; Fibronectin	None	0.13
HR-HER2+	3/4: Slug; c-Myc; Fibronectin	None	0.24

SMD = standardized mean difference for lower- versus higher-survival patient groups, averaged across the four reported markers.

R2.10) The sentence "Consistent with prior findings in JacksonFischer dataset, elevated fibronectin expression has been linked to poor prognosis" in Subsection 2.5 may be misleading, as in previous analyses, fibronectin was only found to be relevant when investigating specific patients in the cohort. Can this be more related to a certain enrichment of these patients in the METABRIC cohort?

RESPONSE: Although METABRIC contains a higher fraction of HR+HER2- patients than JacksonFischer, fibronectin abundance in METABRIC important nodes was not higher in HR+HER2- patients than in other subtypes. Thus, the METABRIC fibronectin signal cannot be explained simply by enrichment of HR+HER2- patients. We therefore revised the manuscript to describe fibronectin as an important-region feature associated

with the lower-survival METABRIC cluster, while avoiding the stronger claim that it represents a standalone prognostic biomarker conserved across cohorts.

ACTION:

- Based on the analysis results, we update the previous fibronectin wording with:

“Because METABRIC contains a higher fraction of HR+HER2- patients than JacksonFischer, we performed a descriptive subtype composition analysis. HR+HER2- patients accounted for 71% of METABRIC patients versus 63% of JacksonFischer patients. Within METABRIC, fibronectin abundance in important nodes was not enriched in HR+HER2- patients relative to other subtypes (mean 5.62 vs. 6.34; Mann–Whitney U test, p = 0.84). We therefore interpret fibronectin as an important feature associated with the lower- survival group in METABRIC dataset, rather than as a standalone prognostic biomarker uniformly conserved across cohorts.”

Reviewer #3 (Remarks to the Author):

I co-reviewed this manuscript with one of the reviewers who provided the listed reports. This is part of the Nature Communications initiative to facilitate training in peer review and to provide appropriate recognition for Early Career Researchers who co-review manuscripts.

Response to Reviewers

Reviewer #1

Remarks to the Author:

I appreciate all the changes and additional experiments/clarifications/discussion made by the authors. These revisions have substantially strengthened the manuscript. I have a few remaining questions/confusions related to the revised texts and additional results, please find them below:

R1.1) Thank authors for updating the figure panels. One follow-up suggestion is for the cluster analysis in Figure 3b/3g/4b/4e: it would also be beneficial to include KM curves grouped by SpaCEy-inferred clusters.

RESPONSE:

We added Kaplan-Meier curves for the clusters derived from SpaCEy embeddings in both the JacksonFischer and METABRIC breast cancer analyses and reported the global log-rank test across clusters. We also added KM curves for the clusters in the HR+HER2-group.

ACTION:

We added supplementary Kaplan-Meier curves for the clusters from the JacksonFischer and the METABRIC cohorts (Supplementary Figure 5 and 7) and updated the Results text to report the log-rank test across clusters:

- Following text were added to JacksonFischer results section:

"Patient groups obtained by clustering of the sample embeddings showed significant global survival separation in JacksonFischer dataset (global log-rank \(p = 0.00253\); Supplementary Fig. 5a)."

"Kaplan-Meier curves for the clusters in HR+HER2- clinical subtype (global log-rank \(p = 0.03132\)) are shown in Supplementary Figure 7a."

- Following text were added to METABRIC results section:

"Patient groups obtained by clustering of the sample embeddings showed significant global survival separation in METABRIC dataset (global log-rank \(p = 0.00371\); Supplementary Fig. 5b)."

"Kaplan-Meier curves for the clusters in HR+HER2- clinical subtype (global log-rank \(p = 0.01498\)) are shown in Supplementary Figure 7b."

R1.4) Thank authors for conducting the new experiments and including additional clarifications. I have a remaining confusion regarding the new "Compartment-resolved

signals within important regions” results in Supp. Table 10. For example: high CD3 (T cell marker) is observed to have big difference in important stromal regions between worse- and better-survival patients. Should I interpret this as: (1) T cells in worse-prognosis patients are expressing more CD3? (2) Worse-prognosis patients had more stromal T cells? (3) Important stromal regions labeled (by SpaCEy) in worse-prognosis patients contain higher proportions of T cells? On top of this, would there be any therapeutic insight that we can acquire here, such as what kind of perturbations on T cells can potentially improve the survival for a worse-prognosis patient?

RESPONSE:

Supplementary Table 10 shows a marker abundance summary within important nodes after grouping those nodes by broad cellular context. Thus, the CD3 result should be interpreted as lower-survival ROIs show higher CD3 marker abundance within important nodes assigned to the stromal context. Regarding the reviewer’s comment on therapeutic insight, SpaCEy can prioritize spatial patterns associated with outcomes, and these patterns may offer potential therapeutic insights by motivating hypotheses related to stromal remodelling, T-cell infiltration, or vascular normalization, as discussed in the cited study below. However, SpaCEy cannot identify a specific therapeutic intervention or determine whether perturbing CD3+ cells would improve survival without causal perturbation experiments or external mechanistic validation. Therefore, these summaries should be considered observational and hypothesis-generating.

ACTION:

- We added an explicit CD3 interpretation note to the JacksonFischer Results section:

"For example, a higher CD3 signal in stromal-context important nodes means that the ROI-level mean CD3 abundance among important nodes assigned to the stromal context is higher in lower-survival ROIs. This suggests the presence of stromal regions with a high abundance of CD3-enriched cells in patients with poorer survival, whereas such regions are largely absent in the better-survival group. Given the immune-excluded phenotype, therapeutic strategies aimed at stromal remodelling or reprogramming, such as combination therapy with anti-TGF-β agents, or at normalization of the tumour vasculature may promote T-cell infiltration into the tumour and potentially improve survival in patients with poor prognosis [32]."

- Added a clarification to the Supplementary Table 10 caption.

"A context-specific marker shift should be interpreted as marker abundance within important nodes assigned to that broad context."

R1.6) Thank authors for all the additional discussion. There are a few remaining questions I want to clarify:

- Maybe I have missed it, the “FoxP3 marker signal corresponded to increased Treg abundance” part is not found in the revised manuscript;

- “49.9% of pooled important Tc cells (3,019 of 6,053 cells) and 40.9% of pooled important Th cells (4,416 of 10,803 cells) in the no-progression group were cancer-adjacent”. On this note, how do these numbers compare to the global (or non-important Tc & Th) statistics?
- “Th cells, CD4+ T cells and CD8+ T cells”. Just want to clarify here that CD4+ T cells and CD8+ T cells are part of the source cell type labels? They don’t seem to appear in Fig. 2d.

RESPONSE:

In the revised manuscript, we now describe Treg cells as T cells enriched for FoxP3 marker based on the source annotation in the original study. We also added the requested context analysis for cancer adjacency. In no-progression samples, 49.9% of important Tc cells were cancer-adjacent compared with 46.8% of all Tc cells, and 40.9% of important Th cells were cancer-adjacent compared with 38.6% of all Th cells. Thus, the important Tc/Th signal is partly tumour-localized, but only modestly enriched relative to the global Tc/Th populations.

Finally, we thank the reviewer for pointing out this ambiguity in the T-cell nomenclature, and it should be clearly defined in the manuscript. We added an explanatory sentence to the Results section to clarify how the T-cell annotations in Fig. 2d relate to the original Sorin \textit{et al.} source annotations based on the marker enrichment.

ACTION:

We revised the LUAD Results section to FoxP3 and Treg cells, added global Tc/Th cancer-adjacency comparisons, and added the general wording of the T-cell label. Specifically, we included the following explanations in the manuscript under the relevant section:

- We added the relation

"... Treg refers to FOXP3-enriched regulatory T cells ....”

- We added a comparative analysis based on the context of Tc and Th cells

"For comparison, 46.8\% of all Tc cells (38,074 of 81,330 cells) and 38.6\% of all Th cells (48,467 of 125,557 cells) in the no-progression group were cancer-adjacent. Thus, important Tc and Th cells were only modestly more tumour-adjacent than the corresponding global Tc/Th populations.”

- Added a clarification sentence regarding the T-cell nomenclature used in the original manuscript. The following sentence updated accordingly based on the standardised nomenclature.

“The T-cell labels follow the source annotations from Sorin et al.: Th cells refers to CD4-enriched helper T cells, Tc refers to CD8a-enriched cytotoxic T cells, Treg refers to FOXP3-enriched regulatory T cells, and T other denotes T cells not assigned to these specific subsets.”

- Updated the results text based on the standardised nomenclature:

"In contrast, non-progressing tumours exhibited areas with elevated numbers of Th cells, Tc cells, and B cells."

R1.8/1.9) Thank authors for the additional experiments and ablation studies. Could you please also include some display items (supp figures, tables, etc.) to support the results? For example, I would love to see highlighted important intra-tumoral CD8 nodes in a real example for ablation study 3.

RESPONSE:

We have now included the requested figure for the synthetic CD8 explainer-validation analysis (Supplementary Fig. S3). These representative samples show that high-importance regions localise to intra-tumoural CD8 cells in positive synthetic graphs, consistent with the quantitative comparison indicating higher importance for intra-tumoural CD8 nodes than for stromal CD8 nodes. We also uploaded the remaining synthetic data and source tables to the data repository.

ACTION:

We added Supplementary Fig. S3 for the synthetic explainer-validation analysis. We also uploaded the remaining synthetic data and source tables to the data repository. We also revised the Results text as follows:

"Representative synthetic samples further showed that high-importance regions localized to intra-tumoral CD8 cells in positive graphs, while stromal CD8 cells outside the tumour compartment received lower importance scores (Supplementary Fig. S3)."

Reviewer #2

Remarks to the Author:

The co-reviewer and I thank the authors for their extensive efforts in revising the manuscript and for providing detailed responses in the rebuttal. The inclusion of Kaplan-Meier curves (Figs. 2a, 3a, 4a), the Cox proportional hazards baseline (Fig. 5), the in silico node-ablation experiments, and the cross-batch transfer analysis using the CODEX colorectal cancer cohort (Section 2.7) are acknowledged as substantial technical additions.

Despite these extensive updates, the core methodological and conceptual concerns regarding the "added value" of SpaCEy's spatial framework and the "circularity" of downstream patient stratification remain fundamentally unresolved. The current analytical logic still suffers from an "information leak" where the model is evaluated on its ability to reconstruct the very signal it was trained to optimize.

More specifically:

Major Concerns

R2.1) Unresolved Circularity in Cluster-Based Stratification and Proof of "Added Value."

In the rebuttal, the authors emphasize that they replaced the original survival boxplots with Kaplan-Meier (KM) curves and log-rank tests (Figs. 3b/3g and 4b/4e) to demonstrate patient stratification. However, this does not resolve the circularity problem highlighted in the first review.

The Core Invalidation: SpaCEy is explicitly trained using survival/progression data as a supervised target. Therefore, the resulting sample-level embeddings are mathematically forced to separate patients based on clinical outcomes. Applying Leiden clustering to these survival-optimized embeddings to generate KM curves is essentially a "re-readout" of the training target. It is statistically guaranteed that these clusters will show a significant log-rank p-value.

Lack of Baseline Control: A trivial, non-spatial baseline model (e.g., a simple supervised PCA/PLS or a standard MLP trained purely on pseudobulk cell-type proportions, with the survival label shown during training) would produce the same qualitative KM separation. The authors have not yet proven that spatial topology or the GNN architecture adds any unique predictive power or biological insight to this stratification beyond what a non-spatial supervised baseline achieves.

Inconsistency in LUAD Dataset (Figure 2c): As noted previously, Figure 2c merely compares marker abundance within "important nodes" between predefined progression and non-progression labels. Since the model was explicitly trained to separate these two groups, recovering these differences is fully expected and could be achieved via a standard differential test on raw bulk/single-cell data.

Therefore, to validate that SpaCEy provides true "added value," the authors must perform at least one of the following:

(1) Run the exact same Leiden clustering and KM pipeline on a non-spatial supervised baseline to prove that SpaCEy's spatial embedding yields a significantly sharper or qualitatively distinct stratification.

(2) Test the learned stratification framework on an independent external validation cohort where survival data were completely withheld during the training phase.

(3) Apply unsupervised clustering to the LUAD embeddings to see if SpaCEy uncovers novel, clinically meaningful subgroups that go beyond the sub-optimal progression labels ($p = 0.0767$).

RESPONSE: We would like to clarify that the purpose of the Kaplan-Meier curves in the first panel of these figures is descriptive. They summarize how observed patient survival is distributed across the relevant patient groups. These analyses were not used for model training, model selection, or independent validation of SpaCEy's predictive performance. Therefore, there is no circularity between those analyses.

To address the requested baseline control as a supporting analysis, we trained a non-spatial supervised multilayer perceptron baseline (MLP) on pseudobulk marker

features from the same samples, without spatial graph topology. The MLP was trained using the same censoring-aware Cox loss as SpaCEy. We then extracted sample-level MLP embeddings, clustered them into three groups, and applied the corresponding patient-level Kaplan-Meier and global log-rank workflow.

We applied the analysis for SpaCEy, the global log-rank p-values across all clusters were 0.00253 for JacksonFischer and 0.00371 for METABRIC (Supplementary Figure 5). For the non-spatial MLP sample-embedding baseline, the corresponding global p-values were 0.23210 for JacksonFischer and 0.12239 for METABRIC (Supplementary Figure 6). The non-spatial MLP baseline did not reproduce significant global cluster-level survival separation in either dataset. Nevertheless, because both SpaCEy and the MLP baseline are trained with survival supervision, we do not present cluster-level Kaplan-Meier separation alone as independent validation of predictive performance. Instead, these curves are used as descriptive embedding-level summaries, while SpaCEy's added value is evaluated together with its spatial graph formulation and spatially localized explanatory analyses.

ACTION:

We added a non-spatial supervised MLP baseline trained with the same censoring-aware Cox loss as SpaCEy and generated patient-level Kaplan-Meier curves with global log-rank tests for both outcome-aligned SpaCEy clusters and MLP sample-embedding clusters. We revised the Results text as follows:

"To compare SpaCEy with a supervised non-spatial representation in terms of embedding-level stratification, we trained a non-spatial multilayer perceptron (MLP) baseline using pseudobulk marker features from the same samples and the same censoring-aware Cox loss used for SpaCEy. We extracted sample-level MLP embeddings, clustered them into three groups, and evaluated patient-level survival using Kaplan--Meier curves and a global log-rank test across all clusters. The non-spatial MLP sample-embedding baseline did not show significant global separation in JacksonFischer (global log-rank \(p = 0.23210\)) or METABRIC (global log-rank \(p = 0.12239\); Supplementary Fig. 6). We therefore use these cluster-level survival analyses as descriptive summaries of supervised representations, not as independent validation of predictive performance."

R2.2) Contradiction Regarding the “Annotation-Free” Paradigm

The manuscript (including the Abstract and Introduction) continues to market SpaCEy as an “annotation-free” tool that models tissues “without requiring predefined cell types.” Yet, looking at the revised downstream analyses, the biological narrative depends almost entirely on cell-type annotations.

The Contradiction: The cell-type-proportion analyses of important nodes (Figs. 2d, 3d, 4d), the new compartment-stratified marker analyses (tumor/stroma/immune/vessel; Supp. Tables 10-11), the cell-type overlays (Fig. 3f), and the cancer-endothelial adjacency discovery (Fig. 2e) all fundamentally require pre-existing cell-type labels. Furthermore, the preprocessing pipeline (Section 4.1) explicitly serializes cell-type annotations.

Therefore, the claim of being "annotation-free" is factually inconsistent with the actual workflow supporting the paper's biological conclusions. The authors should either soften this claim throughout the manuscript (framing it as "cell-type annotation-agnostic during embedding feature-extraction, but annotation-dependent for biological interpretation") or demonstrate that major clinical insights can be derived entirely without cell-type maps.

RESPONSE:

In the first revision, we already clarified that SpaCEy does not use cell-type annotations during model training, embedding generation, or explainer mask optimisation. Cell-type labels are used only in downstream post hoc analyses to aid biological interpretation of the regions identified by the model. To address the reviewer's remaining concern, we further revised the manuscript to describe SpaCEy as annotation-agnostic during model input, training, prediction, and explanation generation, while making clear that cell-type and compartment annotations are used only to assist post hoc biological interpretation. We also clarified the statement in Section 4.1 regarding serialization of cell-type annotations and other meta. Here, "serialization" refers only to saving available metadata fields in the processed data object for later downstream analysis and visualization. It does not indicate that these metadata fields are used to preprocess the model inputs, construct the graphs, or train the model.

ACTION:

- We softened the annotation-free wording in the Abstract and Introduction, clarified the analysis-pipeline description, and revised the preprocessing section to distinguish model inputs from metadata used for interpretation.

"SpaCEy learns directly from molecular marker expression by modelling tissues as spatial graphs of cells and their interactions, without using predefined cell-type labels or anatomical regions as model inputs."

"This workflow is annotation-agnostic during model input, training, prediction, and explanation generation. Annotations are only used to assist post-hoc analysis to contextualize the interpretation to particular groups of samples of interest."

"Cell-type and compartment annotations are not used as SpaCEy node features, but are retained as metadata for descriptive and post hoc biological interpretation."

- We also added the below clarification sentence in Section 4.1.

"Additional metadata, including cell-type and compartment annotations when available, were retained only for downstream post hoc interpretation and visualization, and were not used for graph construction, model training, prediction, or explanation generation.

R2.3) Missing Architectural Specifications (Graph-Level Readout)

While Section 4.2 has been updated to describe the message-passing layers and fully connected layers, it still completely omits the graph-level readout (pooling) operation.

The Issue: A spatial graph contains a variable number of nodes (cells) per tissue sample. To pass this into fully connected layers for sample-level survival prediction or sample-level Leiden clustering, the variable node embeddings must be aggregated into a fixed-length vector. Whether the authors used mean, sum, max, or attention-based pooling is a critical architectural choice that affects both performance and reproducibility.

Thus, the authors should explicitly state the mathematical formulation of the graph-level readout operation in Section 4.2 and state whether different pooling strategies were benchmarked.

RESPONSE:

We have now explicitly described the graph-level readout operation in Section 4.2. In SpaCEy, after the final message-passing layer, node-level embeddings are aggregated into a fixed-length sample-level graph embedding using global mean pooling. The dimensionality of the graph-level embedding was also included as a hyperparameter during optimization. We also clarify that different neighbourhood aggregation strategies were optimised depending on the selected GNN layer architecture. The hyperparameter ranges used for model training are listed in Supplementary Table 6, and the final selected configurations are provided in the deposited repository. We also added the objective function used in the explainer model.

ACTION:

We added a mathematical description of the global mean-pooling readout to Section 4.2 and clarified that the final selected hyperparameter configurations are available in the deposited data repository.

"To obtain a fixed-length sample-level representation from graphs with variable numbers of cells, SpaCEy applies global mean pooling to the final-layer node embeddings. For a graph G = (V, E), the graph-level embedding is computed as

$$h_G = \frac{1}{|V|} \sum_{i \in V} h_i^{(L)},$$

where hi(L) denotes the final message-passing-layer embedding of node i. This pooled graph embedding is then passed to the fully connected layers for survival or classification prediction and is also used as the sample-level representation for UMAP visualization and Leiden clustering. The model-training hyperparameter ranges are listed in Supplementary Table 6."

R2.4) Direct Visual Evidence for Cross-Batch Robustness

The addition of the CODEX colorectal cancer cohort (Section 2.7) for cross-batch transfer analysis is a step in the right direction. However, asserting that SpaCEy is “robust to batch effects” based purely on predictive accuracy metrics is insufficient.

Thus, the authors should provide a UMAP or t-SNE plot of the learned sample-level embeddings for the CODEX dataset, colored side-by-side by (1) technical batch identity

and (2) clinical/immune phenotype. To claim technical robustness, the visualization must clearly demonstrate that samples are grouped by biological signals rather than clustering by the technical batch of origin. Additionally, the text should clarify why a colorectal dataset was used for cross-batch validation of a model largely showcased on breast and lung cancers, and note whether marker panel mismatches limited a breast-to-breast cross-batch transfer.

RESPONSE:

Based on the reviewer's suggestion, we added a UMAP visualization of learned CODEX sample-level embeddings from the A-to-B cross-batch model, with matched panels colored by technical batch and by immune phenotype (Supplementary Fig. S4). This visualization provides a direct qualitative check of whether the learned representation is dominated by technical batch identity in the transfer setting used for the manuscript analysis. In addition, as a quantitative measure, we calculated a technical-batch silhouette score and showed that the embeddings did not exhibit strong separation by technical batch, yielding a score of 0.0009. This indicates that samples were not clearly partitioned according to batch identity and supports the interpretation that the learned representation was not dominated by technical batch effects.

We also clarified why the CODEX colorectal cohort was used. The aim was to test cross-batch transfer in a paired same-marker-panel setting. A direct breast-to-breast cross-batch transfer between JacksonFischer and METABRIC is limited by marker-panel mismatch.

ACTION:

- We added a CODEX A-to-B cross-batch embedding UMAP as Supplementary Fig. S4, revised the CODEX Results section and clarified why a same-panel colorectal CODEX cohort was used instead of a marker-panel-mismatched breast-to-breast transfer analysis. We revised the Results text as follows:

"To provide direct visual evidence for this A-to-B transfer setting, we visualized sample-level embeddings from the cross-batch model using UMAP, with the same samples colored by technical batch and by CLR/DII immune phenotype (Supplementary Fig. S4). The embeddings did not show strong separation by technical batch alone, with a technical-batch silhouette score was calculated as 0.0009, supporting the conclusion that the representation was not dominated solely by batch identity. We therefore interpret this analysis as evidence that SpaCEy retains predictive signal under controlled same-panel cross-batch transfer, rather than as evidence of complete batch-effect invariance."

- We also clarified why CODEX cohort selected for the batch transfer analysis in the main text:

"We selected the CODEX colorectal cohort because matched batch samples share the same marker panel, enabling a controlled same-panel cross-batch test. A direct JacksonFischer-to-METABRIC breast cancer transfer was not used for this analysis because the panels contain different markers."

Minor Issues and Technical Corrections

R2.5) Incomplete Sentence in Section 2.4: The text currently reads: "The elevated expression of fibronectin in the lower-survival HR+HER2- patients points to [44]." The sentence cuts off abruptly after the citation. The actual scientific claim or conclusion must be explicitly filled in.

RESPONSE:

We completed the sentence and revised the wording to match the cited paper more precisely. The revised sentence describes the fibronectin finding as an important-region signal in lower-survival HR+HER2- patients that is consistent with prior METABRIC-based evidence linking high fibronectin expression to decreased patient survival, while also noting that fibronectin's functional role in epithelial-mesenchymal state and metastatic potential is context-dependent.

ACTION:

We replaced the incomplete sentence in Section 2.4 and corrected the citation key.

"The elevated fibronectin signal in important regions from lower-survival HR+HER2- patients is consistent with prior METABRIC-based evidence linking high fibronectin expression in primary breast tumours to decreased patient survival, while also pointing to context-dependent variation in epithelial-mesenchymal state and metastatic potential [45]."

R2.6) Nomenclature Standardization: Technical terms and dataset names remain inconsistent. Please standardize "Space-GM" vs. "SPACE-GM" and "JacksonFischer" vs. "Jackson-Fischer" throughout the text.

RESPONSE:

We standardized the nomenclature throughout the manuscript. The baseline method is now consistently written as "SPACE-GM", and the breast cancer cohort is consistently written as "JacksonFischer".

ACTION:

We corrected the inconsistent nomenclature in the manuscript. Specifically,

- In the LUAD baseline comparison paragraph, all instances of "Space-GM" were changed to "SPACE-GM".

"We evaluated SpaCEy against two state-of-the-art baselines, Ali et al. [4] and SPACE-GM [52], using five-fold cross-validation across all performance metrics (accuracy, F1-score, and AUC) Fig. 2f."

"In terms of accuracy, SpaCEy reached a mean of 0.68, outperforming both Ali et al. (0.61) and SPACE-GM (0.55)."

"Similar trends were observed for F1-score, where SpaCEy got 0.68 on average, whereas the method from Ali et al. and SPACE-GM achieved 0.59 and 0.52, respectively."

"SpaCEy also demonstrated higher discriminative capability, yielding the highest mean AUC (0.62), compared with 0.57 for Ali et al. and 0.52 for SPACE-GM."

- In the baseline comparison section, "JacksonFisher" and "Jackson-Fischer" were changed to "JacksonFischer".

"The results for the JacksonFischer and METABRIC datasets are presented in Fig. 5b and Fig. 5c, respectively."

"On the JacksonFischer dataset, SpaCEy achieved a mean performance of 0.720 ± 0.061, outperforming all 24 non-spatial baseline configurations evaluated through the same fixed 10-fold protocol."

R2.7) Missing Units: In Supplementary Table 5, the column "Peak GPU Memory" lists numerical values (e.g., 235.18) without any units. Please specify "MB" or "GB" in the column header.

RESPONSE:

We now also included units in the Supplementary Table 5 column headers: peak GPU memory is reported in megabytes and average epoch time is reported in seconds.

ACTION:

We revised the Supplementary Table 5 columns in the supplementary material as follows:

"Peak GPU Memory (MB)" "Average Epoch Time (s)"

R2.8) Figure Caption Clarification: For Figure 2f, the caption must explicitly define what the error bars represent (e.g., Standard Deviation or Standard Error of the Mean).

RESPONSE:

We revised the Figure 2f caption to explicitly define the error bars. The caption now states that the error bars indicate the standard deviation across five cross-validation folds, consistent with the five-fold cross-validation used for the baseline comparison.

ACTION:

We updated the Figure 2 caption for panel f.

"f) Comparison of SpaCEy with the state-of-the-art methods. Error bars indicate the standard deviation across five cross-validation folds."

Reviewer #3

Remarks to the Author:

I co-reviewed this manuscript with one of the reviewers who provided the listed reports. This is part of the Nature Communications initiative to facilitate training in peer review and to provide appropriate recognition for Early Career Researchers who co-review manuscripts.

RESPONSE:

We thank Reviewer #3 for co-reviewing the manuscript and for contributing to the evaluation of our work.
